# Pembrolizumab and olaparib in homologous-recombination-deficient metastatic pancreatic cancer: the phase 2 POLAR trial

**DOI:** 10.1038/s41591-026-04299-5

**Published:** 2026-03-25

**Authors:** Wungki Park, Catherine A. O’Connor, Joanne F. Chou, Marc Hilmi, Zeynep Tarcan, Carly Schwartz, Mary Larsen, Ramzi Homsi, Karthigayini Sivaprakasam, Shigeaki Umeda, Maria A. Perry, Anna M. Varghese, Kenneth H. Yu, Fiyinfolu Balogun, Alice Zervoudakis, Seth S. Katz, Tae-Hyung Kim, Ken Zhao, Allison L. Richards, Nicolas Lecomte, Daniel Martin Muldoon, Elias Karnoub, Walid Chatila, Jessica Yang, Imane El-Dika, Devika Rao, Smita Joshi, Michael B. Foote, Ryan Sugarman, James J. Harding, Andrew S. Epstein, David Kelsen, Sree Chalassani, Fergus Keane, Joshua D. Schoenfeld, Anupriya Singhal, Erin Diguglielmo, Chaitanya Bandlamudi, Junmin Song, Hulya Sahin Ozkan, Junguei Hong, Haochen Zhang, Agustin III Cardenas, Maria Lao, Jerry Melchor, Ronak Shah, Wenfei Kang, Francesca Mazzoni, Kevin Soares, Mark TA Donoghue, Ernesto Santos, Vineet Rolston, Marsha Reyngold, Alice Chia-chi Wei, Murray Tipping, Olca Basturk, Michael Berger, Richard Kihn Do, Mark Schattner, William R. Jarnagin, Nadeem Riaz, Vinod Balachandran, Dana Pe’er, Marinela Capanu, Christine Iacobuzio-Donahue, Eileen M. O’Reilly

**Affiliations:** 1 Gastrointestinal Oncology Service, Department of Medicine, Memorial Sloan Kettering Cancer Center, New York, NY USA; 2 David M. Rubenstein Center for Pancreas Cancer Research, Memorial Sloan Kettering Cancer Center, New York, NY USA; 3 Weill Cornell Medical College, New York, NY USA; 4 Parker Institute for Cancer Immunotherapy, San Francisco, CA USA; 5 Harvard Medical School, Boston, MA USA; 6 Department of Epidemiology and Biostatistics, Memorial Sloan Kettering Cancer Center, New York, NY USA; 7 Human Oncology and Pathogenesis Program, Sloan Kettering Institute, Memorial Sloan Kettering Cancer Center, New York, NY USA; 8 Institut Curie, Paris, France; 9 Marie-Josée and Henry R. Kravis Center for Molecular Oncology, New York, NY, USA; 10 Computational Oncology, Department of Epidemiology and Biostatistics, New York, NY USA; 11 Department of Pathology and Laboratory Medicine, Memorial Sloan Kettering Cancer Center, New York, NY USA; 12 Department of Radiology, Memorial Sloan Kettering Cancer Center, New York, NY USA; 13 Immuno-Oncology Program, Memorial Sloan Kettering Cancer Center, New York, NY USA; 14 Molecular Cytology Core Facility, Memorial Sloan Kettering Cancer Center, New York, NY USA; 15 Hepatopancreatobiliary Service, Department of Surgery, Memorial Sloan Kettering Cancer Center, New York, NY USA; 16 The Olayan Center for Cancer Vaccines, Memorial Sloan Kettering Cancer Center, New York, NY USA; 17 Gastroenterology, Hepatology, and Nutrition Service, Department of Medicine, Memorial Sloan Kettering Cancer Center, New York, NY USA; 18 Department of Radiation Oncology, Memorial Sloan Kettering Cancer Center, New York, NY USA; 19 Howard Hughes Medical Institute, Chevy Chase, MD USA; 20 Computational and Systems Biology Program, Sloan Kettering Institute, Memorial Sloan Kettering Cancer Center, New York, NY USA

**Keywords:** Translational research, Pancreatic cancer, Cancer genetics, Tumour immunology, Cancer immunotherapy

## Abstract

Homologous recombination deficiency (HRD) arising from *BRCA1*or *BRCA2* or *PALB2* mutations confers sensitivity to platinum chemotherapy and PARP inhibition in pancreatic cancer (PC) and may enable prolonged disease control with immune checkpoint blockade (ICB). The phase 2 POLAR trial evaluated maintenance pembrolizumab plus olaparib following platinum-based chemotherapy in biomarker-stratified metastatic PC. Sixty-three participants were enrolled into three cohorts: cohort A (*BRCA1*/*BRCA2*-mutated or *PALB2*-mutated HRD, *n* = 33), cohort B (non-core HRD, *n* = 15) and cohort C (platinum sensitive, HRD-wild type, *n* = 15). Cohort A used a two-stage design with co-primary endpoints of at least 43% Response Evaluation Criteria in Solid Tumors (RECIST) objective response rate (ORR) and at least 77% 6-month progression-free survival (PFS) rate. Among RECIST-evaluable participants in cohort A (*n* = 20), ORR was 35% (95% confidence interval (CI): 15−59%), whereas 6-month PFS rate in the full cohort (*n* = 33) was 64% (95% CI: 49−82%), not meeting the primary endpoint. At a median follow-up of 37 months (95% CI: 27−47), median PFS and overall survival (OS) for cohort A were 8.3 (95% CI: 5.3−not reached (NR)) and 28 (95% CI: 12−NR) months, with 2-year and 3-year OS rates of 56% (95% CI: 41−76%) and 44% (95% CI: 28−69%), respectively. In cohorts B and C, ORR was 8% (95% CI: 0−38%) and 14% (95% CI: 2%-43%); median PFS was 4.8 (95% CI: 4.0−12) and 3.3 (95% CI: 1.9−4.8) months; and median OS was 18 (95% CI: 13−NR) and 10 (95% CI: 8.9−24) months, respectively. Preplanned translational analyses showed that circulating tumor DNA response, increased tumor-infiltrating lymphocytes and enrichment of frameshift indel neoantigens were associated with durable clinical benefit. These data suggest that a subset of HRD PC may derive prolonged benefit from PARP-ICB maintenance and support further development of biomarker-guided precision immunotherapy strategies in PC. ClinicalTrials.gov identifier: NCT04666740.

## Abstract (web summary)

Results of the phase 2 POLAR trial show that biomarker-guided treatment in patients with metastatic pancreatic cancer based on homologous repair deficiency leads to encouraging clinical response rates in immune cell-infiltrated tumors.

## Main

PC is projected to become the second leading cause of cancer-related deaths by 2030 (refs. ^1,2^). Despite therapeutic advancements, only modest survival improvements have been achieved with multi-agent chemotherapy regimens, and genomically unselected PC demonstrates near-universal resistance to ICB. In light of the challenges with long-term chemotherapy tolerance, deescalation and maintenance strategies have gained traction as a means to improve quality of life while maintaining disease control^3,4^. Notably, olaparib, a poly-(ADP-ribose) polymerase inhibitor (PARPi), became the first targeted maintenance therapy to demonstrate clinical benefit in genetically selected patients with HRD PC as defined by germline *BRCA1* or *BRCA2* mutations^5,6^.

Precision cancer therapy in biomarker-selected subsets is feasible in PC and has improved survival outcomes when participants receive matched targeted therapies^7,8^. A rare (1%) subset with mismatch repair-deficient (dMMR) PC, defined by a hypermutable phenotype with a higher mutation-derived neoantigen burden, benefits from ICB targeting programmed death 1 (PD-1)^9,10^. Similarly, a significant clinical benefit from platinum-based therapy has been demonstrated in patients with PC harboring a core HRD mutation (<9%), which includes germline or somatic *BRCA1*, *BRCA2* and *PALB2* mutations. PARPi, as monotherapy and in combination with chemotherapy, has been investigated for core as well as non-core HRD (ncHRD) (harboring other candidate HRD gene mutations)^5,11,12^. In parallel, early-phase studies targeting *KRAS* mutations, present in more than 90% of PC, have reported encouraging safety profiles and early signs of clinical activity^13,14^. Finally, in a select subset of resected PC, neoantigen-targeted vaccines against personalized or shared neoantigens have demonstrated encouraging safety and early signs of clinical activity, further highlighting the potential of immune-mediated disease control in genomically defined subsets of PC^15–17^.

Over the past decade, large-scale sequencing efforts have revealed that genomic instability in HRD tumors is associated with characteristic genomic signatures, including the COSMIC signature 3, and large-scale structural changes^18–20^. These structural changes include large-scale state transition (LST), loss of heterozygosity (LOH) and telomeric allelic imbalance (TAI), and tumors with elevated numbers of these events have been shown to be more sensitive to platinum-based chemotherapy and PARPi^19,21,22^. Interestingly, some of these mutational patterns, such as increased frameshift indels and specific genomic signatures, particularly in tumors with *BRCA2* mutations, may contribute to increased immunogenicity^19,23–26^. Lastly, emerging data suggest that mutations in ncHRD genes (for example, *RAD51B*, *RAD51C*, *RAD51D*, *ATM*, *CHEK2*, *BAP1*, *BARD1* and *BRIP1*) may confer HRD-like biology and warrant further clinical and mechanistic evaluation^19,22,27^.

The POLAR trial is a phase 2, single-center, non-randomized, biomarker-selected basket study evaluating the combination of pembrolizumab (anti-PD-1) and olaparib (PARP) inhibitors as maintenance therapy in participants with metastatic PC who achieved disease control after platinum-based chemotherapy (NCT04666740)^28^. Given the known clinical benefit of PARPi in core HRD tumors and the investigational predictive value of ncHRD mutations or platinum sensitivity alone, participants were prospectively stratified into three cohorts: (A) HRD by *BRCA1, BRCA2* and *PALB2* mutations, (B) ncHRD gene mutations and (C) platinum-sensitivity without HRD mutations. Here we report clinical outcomes across all three cohorts, along with translational analyses, including mutational signatures, neoantigen and immune correlates, to identify biological features associated with therapeutic response.

## Results

### Study design

**Fig. 1:**
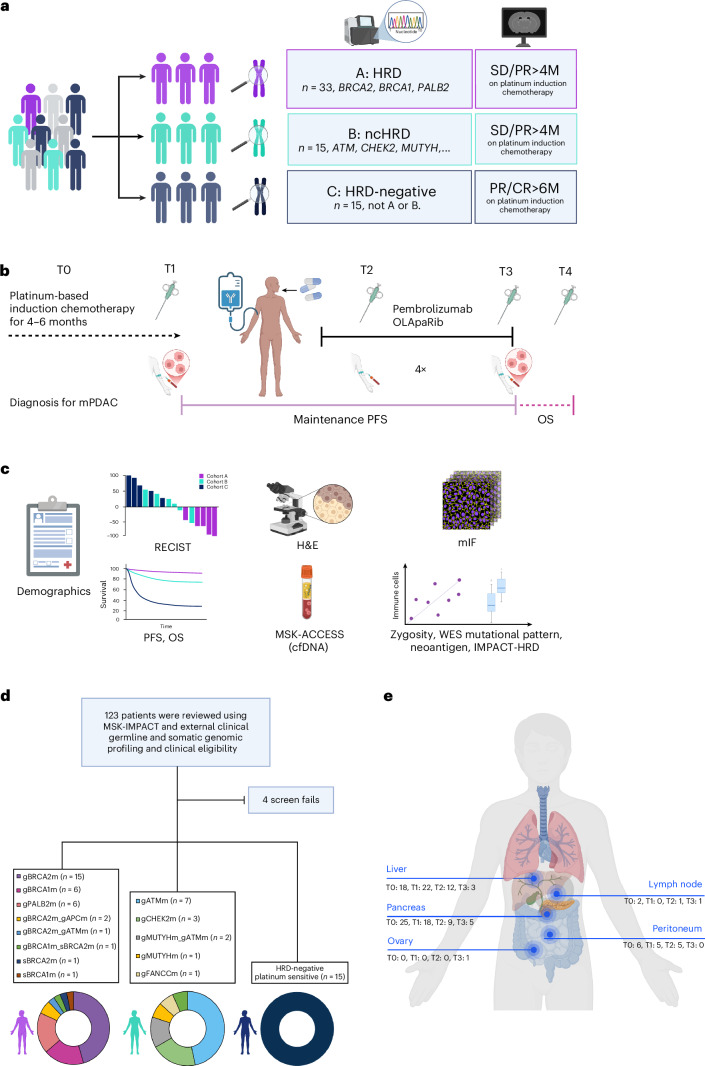
Schema of the POLAR trial: precision immunotherapy for genetically and phenotypically defined subsets of metastatic PC. **a**, Cohort stratification. Participants with mPC were prospectively assigned to one of three cohorts based on germline or somatic mutation profiling and clinical response to platinum-based chemotherapy: cohort A (HRD, *n* = 33): core HRR gene mutations (*BRCA1*, *BRCA2* and *PALB2*); cohort B (ncHRD, *n* = 15): non-core HRR mutations (for example, *ATM*, *CHEK2* and *MUTYH*); and cohort C (HRD negative, *n* = 15): no HRD-associated mutations but ≥6 months of platinum sensitivity. **b**, Trial timeline. Schematic overview of treatment course: platinum-based induction chemotherapy (T0–T1), maintenance with pembrolizumab and olaparib, with PFS (T1–T3), on-treatment biopsy (T2) and OS tracked throughout. **c**, Correlative endpoints. Integrated clinical and translational analyses included demographics, RECIST version 1.1 response, PFS/OS, ctDNA profiling (MSK-ACCESS), WES for mutational patterns, zygosity, neoantigens and TME assessment by H&E and mIF. **d**, Screening and cohort allocation. Among 123 patients screened using MSK-IMPACT and external germline/somatic testing, 63 were enrolled and assigned to each cohort. Bottom, genomic breakdown by gene for each cohort. **e**, Biospecimen collection sites across timepoints. Anatomical distribution of metastatic biopsies collected longitudinally (T0–T3), with most samples from liver, pancreas and peritoneum. cfDNA, cell-free DNA; CR, complete response; M, months; mPC, metastatic pancreatic cancer; PR, partial response; SD, stable disease.

The POLAR trial enrolled *n* = 63 participants into three cohorts based on molecular classification and their PC response to platinum-based chemotherapy. Cohort A included participants with pathogenic germline or somatic mutations in HRD genes (*BRCA2*, *BRCA1* and *PALB2*), without progressive disease after more than 4 months of platinum-based chemotherapy; cohort B (ncHRD) included participants with pathogenic germline or somatic mutations in ncHRD genes (*ATM*, *BAP1*, *BARD1*, *BLM*, *BRIP1*, *CHEK2*, *FAM175A*, *FANCA*, *FANCC*, *NBN*, *RAD50*, *RAD51*, *RTEL1* and *MUTYH*), also without progressive disease over 4 months of platinum-based chemotherapy; and cohort C included participants with platinum sensitivity for at least 6 months (12 cycles of modified FOLFIRINOX) but without HRD or ncHRD gene mutations (Fig. 1a).

Participant enrollment occurred between 11 January 2021 and 7 March 2024. All participants received maintenance olaparib 300 mg orally twice a day daily and pembrolizumab intravenously every 3 weeks for 6 months and then every 6 weeks until disease progression or unacceptable toxicity. Select participants were allowed to continue beyond radiographic progression if deriving clinical benefit.

Serial tumor tissue (baseline, on-treatment and after progression when feasible) and matched peripheral blood samples were collected (Fig. 1b−d). These were used to investigate genomic instability, tumor immune microenvironment and neoantigen landscape features associated with therapeutic response and resistance. Specifically, whole-exome sequencing (WES), cell-free DNA and multiplex immunofluorescence (mIF) were employed from available biospecimens collected from different sites at different timepoints (Fig. 1c,e) to evaluate mutational signatures, neoantigens and immune infiltration across responders and non-responders (Methods). This translational framework enabled broad exploratory analyses of biological correlates of response to POLAR.

### Baseline characteristics

Baseline clinical characteristics are summarized in Table 1. The median age at diagnosis was numerically similar across cohorts (median age, 62−65 years), with balanced sex distribution. Most participants presented with de novo stage IV disease, and all had an Eastern Cooperative Oncology Group (ECOG) performance status of 0 or 1. Tumor histology was predominantly adenocarcinoma, with a small number of acinar (*n* = 3) and adenosquamous (*n* = 1) carcinomas in cohort A. Baseline carbohydrate antigen 19-9 (CA19-9) and carcinoembryonic antigen (CEA) values at enrollment were similar between cohorts A and B but higher in cohort C, consistent with greater disease burden. Molecular characteristics at enrollment of the 63 participants are summarized in Fig. 1d and Table 2. Among cohort A, *BRCA2* mutations were the most common (*n* = 18, 53%), followed by *BRCA1* (*n* = 10) and *PALB2* (*n* = 6). In cohort B, *ATM* mutations were the most common (*n* = 8, 53%), followed by *CHEK2* mutations in *n* = 3 (20%) and *BLM* (*n* = 1), *FANCC* (*n* = 1), *MUTYH* (*n* = 1) and *MUTYH*_+*ATM* (*n* = 1) co-mutation. Cohort C had no detectable HRD or ncHRD mutations by design.

**Table 1:** Demographic and baseline clinical characteristics of POLAR trial participants by biomarker-defined cohort

Characteristica	Overall (*n* = 63)	A (*n* = 33)	B (*n* = 15)	C (*n* = 15)
Age, years				
Median (Q1, Q3)	62 (53, 70)	62 (51, 70)	62 (59, 69)	65 (58, 70)
Sex				
Female	33 (52%)	18 (55%)	7 (47%)	8 (53%)
Male	30 (48%)	15 (45%)	8 (53%)	7 (47%)
Race				
Asian	6 (9.5%)	2 (6.1%)	1 (6.7%)	3 (20%)
Black	2 (3.2%)	0 (0%)	1 (6.7%)	1 (6.7%)
Unknown	2 (3.2%)	2 (6.1%)	0 (0%)	0 (0%)
White	53 (84%)	29 (88%)	13 (87%)	11 (73%)
ECOG performance status				
0	36 (57%)	21 (64%)	6 (40%)	9 (60%)
1	27 (43%)	12 (36%)	9 (60%)	6 (40%)
Stage at diagnosis				
II	6 (9.5%)	4 (12%)	2 (13%)	0 (0%)
III	4 (6.3%)	4 (12%)	0 (0%)	0 (0%)
IV	53 (84%)	25 (76%)	13 (87%)	15 (100%)
Tumor location				
Body	13 (21%)	7 (21%)	2 (13%)	4 (27%)
Head	30 (48%)	16 (48%)	7 (47%)	7 (47%)
Tail	20 (32%)	10 (30%)	6 (40%)	4 (27%)
Histology				
Acinar	3 (4.8%)	3 (9.1%)	0 (0%)	0 (0%)
Adenocarcinoma	59 (94%)	29 (88%)	15 (100%)	15 (100%)
Adenosquamous	1 (1.6%)	1 (3.0%)	0 (0%)	0 (0%)
Prior surgery	7 (11%)	5 (15%)	2 (13%)	0 (0%)
Platinum type				
Cisplatin	7 (11%)	7 (21%)	0 (0%)	0 (0%)
Oxaliplatin	56 (89%)	26 (79%)	15 (100%)	15 (100%)
CA 19-9 at baseline (T1)				
Median (Q1, Q3)	52 (20, 344)	55 (19, 187)	49 (24, 383)	144 (27, 985)
Unknown	5	3	2	0
CEA at baseline (T1)				
Median (Q1, Q3)	3 (2, 7)	3 (2, 5)	4 (3, 6)	8 (2, 10)

Baseline clinical characteristics are presented for all 63 participants enrolled in the POLAR trial, stratified by biomarker-defined cohorts: cohort A (core HRD; *n* = 33), cohort B (ncHRD; *n* = 15) and cohort C (HRD negative, platinum sensitivity; *n* = 15). Variables include age, sex, race, initial stage at diagnosis, histologic subtype, prior surgery and platinum agent received during induction therapy. ECOG performance status at trial entry is also shown.

^a^Data shown as *n* (%) unless indicated otherwise.

**Table 2:** Clinical and molecular characteristics of POLAR trial participants stratified by biomarker-defined cohort

Characteristic^a^	Overall (*n* = 63)	A (*n* = 33)	B (*n* = 15)	C (*n* = 15)
Family history of PC	6 (9.5%)	3 (9.1%)	1 (6.7%)	2 (13%)
POLAR BOR (*n* = 46)				
CR	1 (2.2%)	0 (0%)	0 (0%)	1 (7.1%)
PR	9 (20%)	7 (35%)	1 (8.3%)	1 (7.1%)
SD	22 (48%)	9 (45%)	8 (67%)	5 (36%)
PD	14 (30%)	4 (20%)	3 (25%)	7 (50%)
Undefined	17	13	3	1
TMB (*n* = 42)				
Median (Q1, Q3)	4.10 (2.53, 5.20)	4.90 (3.30, 6.40)	2.90 (1.60, 4.53)	4.10 (2.60, 5.00)
Min, max	0.80, 14.80	1.60, 14.00	0.80, 14.80	1.60, 8.20
Unknown	21	15	3	3
IMPACT-HRD (*n* = 34)				
Median (Q1, Q3)	31 (14, 47)	47 (24, 59)	29 (15, 44)	24 (14, 35)
Min, max	1, 67	4, 67	7, 67	1, 47
Unknown	29	19	5	5
Neoantigen burden (*n* = 35)				
Median (Q1, Q3)	155 (54, 355)	218 (131, 379)	137 (63, 344)	123 (24, 191)
Min, max	8, 1733	8, 856	29, 1733	9, 470
Unknown	28	17	5	6
TIL density (*n* = 38)				
High	22 (58%)	14 (78%)	4 (50%)	4 (33%)
Low	16 (42%)	4 (22%)	4 (50%)	8 (67%)
Unknown	25	15	7	3
NLR at baseline (T1) (*n* = 63)				
Median (Q1, Q3)	3.3 (1.9, 6.1)	3.1 (1.9, 5.3)	2.6 (1.8, 5.8)	3.4 (2.6, 6.3)
Min, max	0.4, 37.3	0.4, 12.4	0.7, 37.3	0.5, 32.5
Mean VAF of ctDNA at baseline (T1) (*n* = 30)				
Detected	13 (43%)	8 (57%)	3 (38%)	2 (25%)
QC fail	3 (10%)	1 (7.1%)	0 (0%)	2 (25%)
Undetected	14 (47%)	5 (36%)	5 (63%)	4 (50%)
Unknown	33	19	7	7

BOR, best overall response (RECIST version 1.1); CR, complete response; PD, progressive disease; PR, partial response; QC, quality check; SD, stable disease.

This table summarizes key clinical responses, genomic biomarkers and immune features across the three POLAR cohorts: (A) HRD, (B) ncHRD and (C) HRD negative platinum sensitive (HRP). Parameters include family history of PC and BOR by RECIST (CR, PR, SD and PD). Median TMB, genomic instability score (GIS), predicted neoantigen load and TIL density at baseline (T01) are reported. CA19-9, CEA and NLR at T1 are also shown.

^a^Data shown as *n* (%) unless indicated otherwise.

### Primary outcomes

**Fig. 2:**
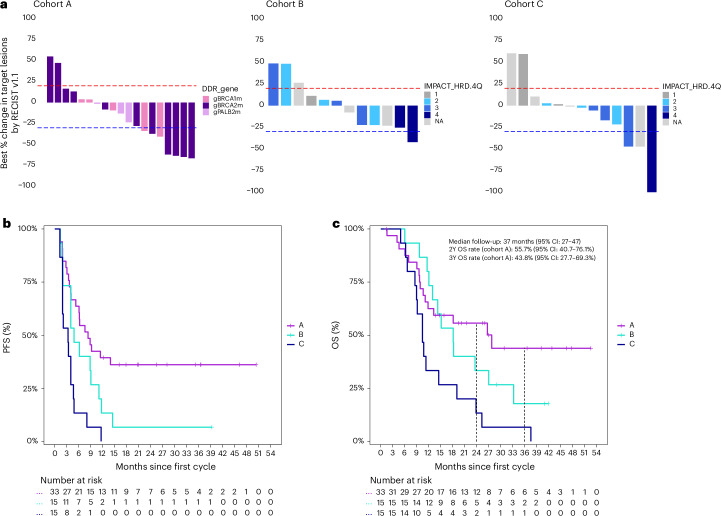
Clinical and molecular outcomes in the POLAR trial. **a**, Waterfall plots show the best percent change in target lesion size per RECIST version 1.1 in cohorts A, B and C. In cohort A, bars are color coded by the gene harboring a core HRD mutation: *BRCA1*, *BRCA2* and *PALB2*. In cohorts B and C, bars are shaded by IMPACT-HRD quartile, a genomic scar score derived from MSK-IMPACT targeted sequencing. Higher quartiles (3−4, darker blue) reflect greater levels of genomic instability associated with HRD and were used to stratify ncHRD (B) and HRD-negative (C) tumors. **b**, Kaplan–Meier curves for PFS by cohort. Median PFS was 8.3 months in cohort A, 4.8 months in cohort B and 3.3 months in cohort C. **c**, Kaplan–Meier curves for OS by cohort. Median follow-up was 37 months (95% CI: 27−47). In cohort A, the 2-year OS rate was 56% (95% CI: 41−76%), and the 3-year OS rate was 44% (95% CI: 28–69%). Dashed lines indicate 24-month and 36-month landmarks. Number at risk is shown below each plot. Y, year; NA, not available. Source data

Among 63 participants enrolled in the POLAR trial, 46 (73%) had RECIST-evaluable disease at initiation of maintenance therapy, as assessed by independent radiology review. Objective responses were observed across all three cohorts, although the number of responses varied by genomic context (Fig. 2a). In cohort A, the ORR was 35% (7/20; 95% CI: 15−59%), which did not meet the prespecified co-primary endpoint. In cohorts B and C, the ORR was 8% (1/12; 95% CI: 0−38%) and 14% (2/14; 95% CI: 2−43%), respectively.

Notably, 13 of 33 patients (39%) in cohort A had non-measurable ‘undefined’ disease at baseline due to profound radiographic responses to prior platinum-based chemotherapy, a prerequisite for POLAR eligibility; all had baseline measurable disease before platinum exposure. Among these participants, 10 remained progression free on POLAR maintenance for more than 4 months (two with PFS > 6 months and eight with PFS > 12 months)—a time window commonly used to confirm radiographic response on repeat imaging—and all had a minimum follow-up of 16 months. These findings are consistent with favorable underlying HRD biology. In a post hoc exploratory analysis that included these participants with durable PFS > 4 months, the exploratory response rate for cohort A increased to 52% (95% CI: 34−69%; 17/33), suggesting enrichment for HRD-associated disease and the inherent platinum sensitivity.

The PFS for each of the three cohorts is presented in Fig. 2b. At the time of data cutoff (25 June 2025), the 6-month PFS rate was highest in cohort A at 64% (95% CI: 49−82%); however, it did not meet the co-primary endpoint of 6-month PFS rate of 77% (the protocol is attached as supplementary data). This was followed by cohort B at 47% (95% CI: 27−80%) and cohort C at 13% (95% CI: 3.7−48%). Median PFS was 8.3 months (95% CI: 5.3−NR), 4.8 months (95% CI: 4−12) and 3.3 months (95% CI: 1.9−4.8) for cohorts A, B and C, respectively. Stratified multivariable Cox analysis showed that baseline CA 19-9 was significantly associated with PFS (hazard ratio = 1.02, 95% CI: 1.02−1.03) independently of other known risk factors, including age, tumor location, duration of prior platinum therapy, gender, ECOG performance status and initial stage.

### Secondary outcomes

Disease control was achieved in the majority of participants, with disease control rate (DCR) of 80% in cohort A (16/20; 95% CI: 56–94%), 75% in cohort B (9/12; 95% CI: 43–95%) and 50% in cohort C (7/14; 95% CI: 23–77%). These findings mirrored the trend observed across PFS and OS, supporting the clinical relevance of genomic stratification. Among cohort A evaluable responders (complete response/partial response, *n* = 7), the median duration of response (DoR) was 6.8 months (95% CI: 3.4–NR). When including patients with non-measurable (labeled as ‘undefined’) disease at baseline (*n* = 13), the median duration of disease control was 32 months (95% CI: 6.1–NR). Median follow-up was 37 months (95% CI: 27–47), and median OS for cohort A was 28 months (95% CI: 12–NR), 18 months (95% CI: 13–NR) for cohort B, followed by 10 months (95% CI: 8.9–24) for cohort C (Fig. 2). In cohort A (Fig. 2c), the 2-year OS rate was 56% (95% CI: 41–76%), and the 3-year OS rate was 44% (95% CI: 28–69%). Stratified multivariable Cox analysis showed that baseline CA 19-9 was significantly associated with OS after adjusting for known baseline risk factors (hazard ratio = 1.02, 95% CI: 1.02–1.03) (Supplementary Table 1). Additional prespecified secondary endpoints not reported in this paper include best overall response assessed by immune RECIST (iRECIST), biomarker response endpoints (CEA and CA 19-9 kinetics) and RECIST/iRECIST-concordant PFS analyses, which will be reported separately.

### Subgroup analysis by *BRCA2*, *PALB2*, *BRCA1* and ncHRD gene mutations

Subgroup analysis by mutation type within cohort A (HRD) and cohort B (ncHRD) revealed heterogeneity in clinical outcomes. Participants with *BRCA2* (*n* = 18) and *PALB2* (*n* = 6) mutations demonstrated numerically similar PFS and OS but longer than participants with *BRCA1* mutations (*n* = 9) (Supplementary Fig. 2). The median PFS was 9.9 months (95% CI: 3.6−NR), 12.0 months (95% CI: 6.2−NR) and 6.1 months (95% CI: 4.1−NR) for *BRCA2, PALB2* and *BRCA1*, respectively The median OS was 28.0 months (95% CI: 9.9−NR), 27.0 months (95% CI: 11−NR) and 18.0 months (95% CI: 12−NR), respectively. At 24 months, OS rates were 59% (95% CI: 40−88%) for *BRCA2*, 67% (95% CI: 38−100%) for *PALB2* and 42% (95% CI: 18−94%) for *BRCA1*. From cohort A (*n* = 33), 15 participants had both germline and somatic sequencing data available, and the rest (*n* = 18) had only circulating tumor DNA (ctDNA) available. For zygosity status: 10 biallelic loss (seven *BRCA2*, two *PALB2* and one *BRCA1*), three monoallelic loss (two *BRCA2* and one *PALB2)*and two indeterminate (one *BRCA2* and one *BRCA1*) (Supplementary Table 1). In cohort B, *ATM* mutations were the most common type (*n* = 9, 60%). Participants with *ATM* and non*-ATM* ncHRD gene mutations (*CHEK2*, *FANCC* and *MUTYH*) had similar median PFS to other ncHRD mutations (4.8 months (95% CI: 2−NR) versus 6.5 months (95% CI: 4−NR)) (Supplementary Fig. 3). Participants with *ATM* mutation had numerically longer OS (18 months (95% CI: 15−NR) versus 14 months (95% CI: 12−NR)).

### Safety

Treatment was generally well tolerated in line with the known adverse event profiles of both drugs, and no new safety signals were observed. No grade 4 or 5 treatment-related adverse events (TRAE) occurred. Grade 3 TRAEs included anemia (*n* = 10, 15%) and abdominal infection (*n* = 1, 1.6%). Grade 2 immune-related adverse events (irAEs) included colitis (*n* = 1), hyperglycemia (*n* = 1), pneumonitis (*n* = 2), pancreatitis (*n* = 1) and hyperthyroidism (*n* = 1). Grade 3 irAEs occurred in *n* = 4 participants and included pneumonitis and colitis (Table 3).

**Table 3:** TRAEs and irAEs

Olaparib-related adverse events					
		Total (*n* = 63)	A (*n* = 33)	B (*n* = 15)	C (*n* = 15)
Grade 3	Anemia	13* (20.6%)	9 (27.2%)	2 (13.3%)	2 (13.3%)
	Pneumonitis	1 (1.6%)	1 (3%)	0	0
	Hyperglycemia	1 (1.6%)	0	0	1 (6.7%)
Grade 2	Anemia	7 (11%)	6 (18.1%)	1 (6.7%)	0
	Fatigue	5 (7.9%)	4 (12.1%)	0	1 (6.7%)
	Nausea	3 (4.8%)	2 (6.1%)	0	0
	Neutropenia	2 (3.2%)	0	1 (6.7%)	0
	Gout	1 (1.6%)	1 (3%)	0	0
	Hypotension	1 (1.6%)	1 (3%)	0	0
* 29 events across 13 patients			** 25 events across seven patients		
**Pembrolizumab-related adverse events**					
		**Total** (***n*** = **63)**	**A** (***n*** = **33)**	**B** (***n*** = **15)**	**C** (***n*** = **15)**
Grade 3	Diarrhea	2 (3.2%)	0	1 (6.7%)	1 (6.7%)
	Pneumonitis	1 (1.6%)	1 (3%)	0	0
	Hyperglycemia	1 (1.6%)	0	0	1 (6.7%)
Grade 2	Pneumonitis	2 (3.2%)	2 (6.1%)	0	0
	Hyperglycemia	1 (1.6%)	1 (3%)	0	0
	Hyperthyroidism	1 (1.6%)	1 (3%)	0	0
	Colitis	1 (1.6%)	1 (3%)	0	0
	Diarrhea	1 (1.6%)	1 (3%)	0	0

Grade 2 and grade 3 adverse events attributed to olaparib (top) and pembrolizumab (bottom) are shown. For olaparib, grade 3 anemia occurred in 10 patients (100% of grade 3 events), and grade 2 events were reported in 15 patients, with anemia, fatigue and nausea as the most common. For pembrolizumab, grade 3 events occurred in three patients, including diarrhea, pneumonitis and hyperglycemia. irAEs attributed to pembrolizumab included pneumonitis, colitis, hyperthyroidism and hyperglycemia. Counts reflect the number of events and the proportion of affected patients. No grade 4 or grade 5 treatment-related events were observed.

### Exploratory integrated biomarker analyses

Exploratory analyses reported in this paper were prespecified in the trial protocol and were conducted to investigate molecular and immunologic correlates of clinical benefit. These include analyses of ctDNA dynamics, tumor genomic features and mutational patterns, immune infiltration and associations with clinical outcomes. Specific analytic implementations, subgroup definitions and correlation analyses were exploratory and intended to generate hypotheses rather than to formally test prespecified endpoints. In addition, selected post hoc analyses were performed to further explore emerging signals observed in the data and are clearly identified as such in the paper.

### ctDNA dynamics and clinical benefit

**Extended Data Fig. 1:**
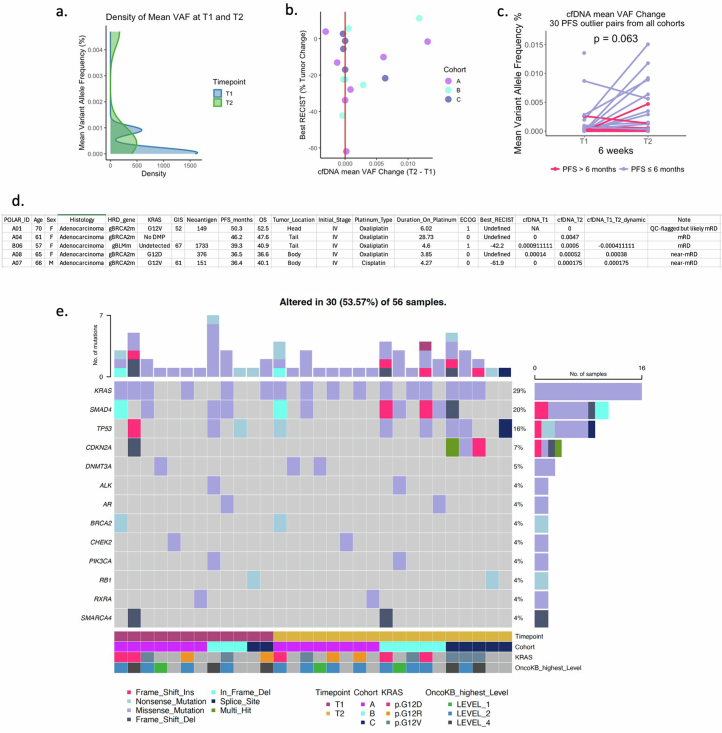
Plasma-based mutational profiling using MSK-ACCESS emphasizes molecular minimal disease dynamics during POLAR maintenance. (**a**) Density plot showing the distribution of mean variant allele frequency (VAF) at baseline (T1) and on-treatment (T2) timepoints. Most samples exhibited very low VAF at T1, consistent with molecular residual disease (mRD) at T1. (**b**) Scatterplot of ctDNA VAF change (T2 – T1) versus best RECIST (% tumor change). A red vertical line marks the radiographic response (RECIST v1.1) at 0%. (**c**) Paired line plot showing ctDNA mean VAF dynamics over 6 weeks in 30 representative plasma pairs across cohorts A–C, stratified by PFS ≤ 6 months (purple) and > 6 months (red). A trend toward lower VAF change was observed in long-term responders (p = 0.063). (**d**) Summary table of select participants with durable PFS ( > 36 months), highlighting their HRD status, neoantigens, PFS, treatment details, and ctDNA metrics. Four of the five patients had undetectable or near-undetectable ctDNA at both timepoints, suggesting a link between mRD negativity and durable benefit. (**e**) MSK-ACCESS Oncoprint summarizing somatic alterations detected by MSK-ACCESS in 56 evaluable samples. Only genes altered in ≥2 samples are shown. Mutation type, timepoint (T1 vs T2), OncoKB level, and gender are annotated. Abbreviations: ctDNA, circulating-tumor DNA; VAF, variant allele frequency; PFS, progression-free survival; mRD, molecular residual disease; TMB, tumor mutational burden; T1, baseline; T2, 6-week on-treatment; OncoKB, Onco Knowledge Base. Source data

To explore the association between early ctDNA dynamics and clinical outcomes, we analyzed 30 representative participants’ plasma pairs (*n* = 60) from baseline (T1) and 6-week (T2) plasma across the three cohorts (A: *n* = 14 pairs, B: *n* = 8 pairs, C: *n* = 8 pairs) using MSK-ACCESS. Of these, *n* = 56 samples (93%) passed quality control, and only *n* = 32 (57%) had detectable somatic mutations. Notably, most samples at T1 had low mean variant allele frequency (VAF) less than 0.004 (Extended Data Fig. 1a), indicative of molecular residual disease (mRD). Change in mean VAF between T1 and T2 were generally minimal (range, −0.0031 to 0.013), and a trend toward higher VAF increases was observed among participants with PFS ≤ 6 months compared to those with PFS > 6 months (*P* = 0.063; Extended Data Fig. 1b,c). At data cutoff, five participants (7.9%) achieved durable clinical benefit with PFS exceeding 36 months. Four of these had undetectable or near-undetectable mean VAF (0.00014 and 0.00009) at both T1 and T2 timepoints; the T1 sample of the fifth participant did not pass quality control, but T2 mean VAF was 0 (Extended Data Fig. 1d). Despite unavailability of direct tumor tissue genomics in many participants, using *n* = 56 MSK-ACCESS ctDNA samples, we were able to recreate the OncoPrint summarizing their somatic alterations (Extended Data Fig. 1e).

### HRD genotype and irAEs in relation to durable benefit

**Fig. 3:**
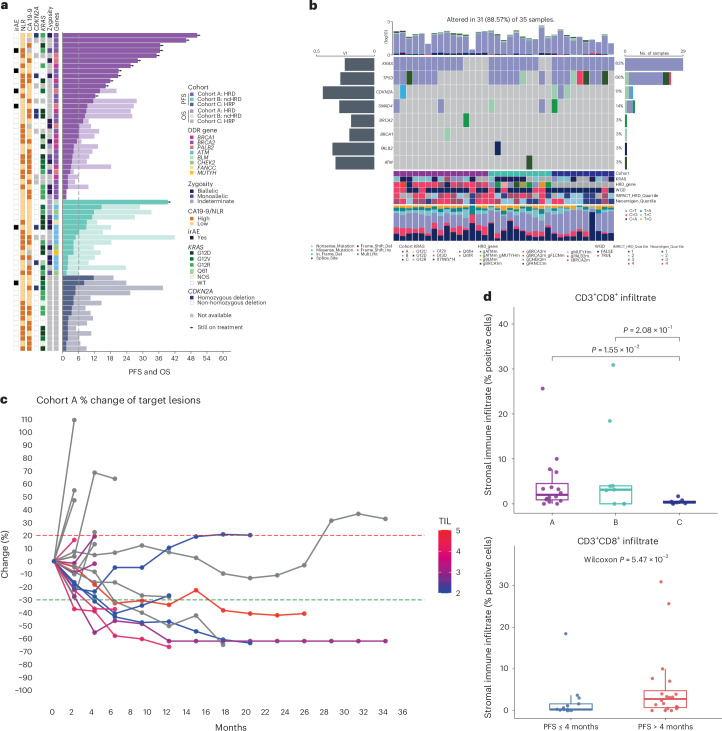
Clinical, genomic and immunologic parameters associated with response to pembrolizumab and olaparib in the POLAR trial. **a**, Swimmer plot showing PFS (solid bar) and OS (shaded bar) annotated with clinical and genomic features for all POLAR participants, stratified by each cohort. Bars are annotated by enrollment DDR gene (for example, *BRCA1*, *BRCA2*, *PALB2*, *ATM* and *CHEK2*), zygosity, baseline CA19-9 level, NLR, irAE status, *KRAS* allele and *CDKN2A* homozygous deletion status. **b**, OncoPrint with WES showing genomic alterations across POLAR patients (*n* = 35) with annotation tracks for cohort, *KRAS* allele, HRD gene class, WGD, IMPACT-HRD scores and neoantigen quartile. **c**, Spaghetti plot: longitudinal changes of tumor response in patients with different levels of TIL in tumors (H&E) of the participants from cohort A. Lines are colored by TIL density score. TIL-high tumors were associated with longer PFS. **d**, mIF CD8^+^ T cell analysis: from tumors at baseline with mIF available (*n* = 33). Cohort A had higher CD3^+^CD8^+^ T cell infiltration (*P* < 0.05), and participants with longer PFS (>4 months) had higher TIL density (*P* = 0.055). Source data

To explore the relationship between clinical and molecular features associated with clinical benefit, we generated participant-level swimmer plots (Fig. 3a) integrating PFS, genomic alterations (mutations in DNA damage response (DDR), HRD and ncHRD genes and *KRAS* and homozygous deletion of *CDKN2A*), baseline biomarkers (CA19-9 and neutrophil-to-lymphocyte ratio (NLR)) and irAEs. Eleven durable responses were predominantly observed in cohort A, including exceptionally long-term responders (PFS > 18 months) with *BRCA2* (*n* = 8), *PALB2* (*n* = 2) and *BRCA1* (*n* = 1) germline mutations. One participant in cohort B with a germline *BLM* mutation has remained on POLAR for more than 36 months with a durable partial response (−42.2% by RECIST version 1.1). All irAEs (*n* = 6) occurred among patients who had PFS > 6 months across three cohorts. Other variables such as *KRAS* mutation allele, CDKN2A loss and baseline NLR did not separate groups with different PFS outcome.

### Cohort A (HRD) tumors are enriched for immunogenic mutational patterns and immune infiltration

**Extended Data Fig. 2:**
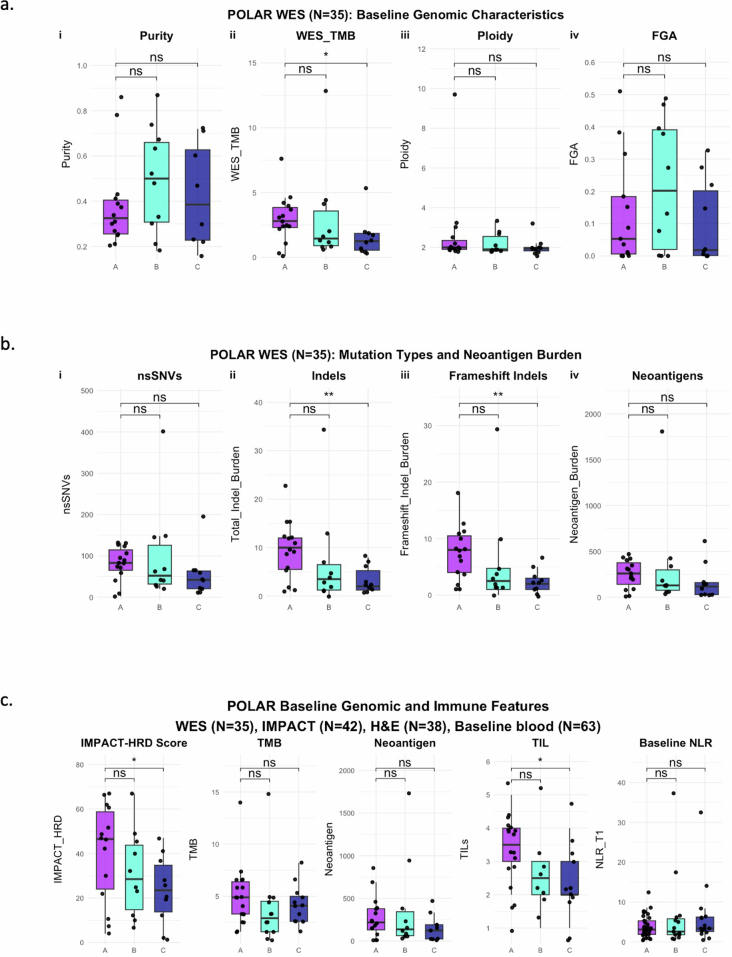
Immunogenomic context of tumors from POLAR. **a**) **Baseline genomic characteristics from WES (N** = **35) stratified by POLAR cohort (A: HRD, B: ncHRD, C: HRP)**: (i) Tumor purity, (ii) WES-derived tumor mutational burden (WES_TMB) was significantly higher in Cohort A (HRD) *p* < 0.05, (iii) ploidy, and (iv) fraction genome altered (FGA). No statistically significant differences were observed across cohorts. (**b**) **Mutation types and neoantigen burden:** (i) Number of nsSNVs, (ii) total indel burden, (iii) frameshift indel burden, and (iv) predicted neoantigen burden. Indel and frameshift indel burden were significantly higher in Cohort A (HRD). Statistical comparisons were performed using Wilcoxon rank-sum tests. Significance is indicated by *p* values (*p* < 0.01). (**c**) **Baseline genomic and immune features across platforms:** IMPACT HRD score (N = 35), IMPACT TMB (N = 42), neoantigen burden from WES (N = 35), TIL density from H&E (N = 38), and baseline neutrophil-to-lymphocyte ratio (NLR) from blood (N = 63). No significant differences were observed between cohorts in these parameters. Each dot represents an individual patient. Boxes indicate median and interquartile range; whiskers represent 1.5× IQR. Cohorts: A (purple), B (teal), C (navy). Statistical significance was assessed by two-sided Wilcoxon test and annotated as: ns (not significant), *p* < 0.05, *p* < 0.01. **Abbreviations:** WES, whole exome sequencing; HRD, homologous recombination deficiency; ncHRD, non-core homologous recombination deficiency; nsSNVs, nonsynonymous single-nucelotide variants; OS, overall survival; PFS, progression-free survival. Source data

WES of 35 baseline tumors (*n* = 15, *n* = 10 and *n* = 10 in cohorts A, B and C, respectively) revealed that cohort A (HRD) exhibited a distinct and significantly more immunogenic mutational landscape compared to cohort C (platinum sensitive without HRD) (Fig. 3b and Extended Data Fig. 2). Specifically, both total indel burden (median of 10 (interquartile range (IQR): 5−12)) and frameshift indel burden (median: 8 (IQR: 4−11)) were significantly higher in cohort A than in cohort C (median of 2 for both parameters) (*P* < 0.01), indicating active error-prone repair processes that preferentially generate mutation-derived neoantigens (Extended Data Fig. 2b). Although non-synonymous single-nucleotide variant (SNV) burden and total predicted neoantigen burden did not differ significantly across cohorts, this enrichment of frameshift indel mutations in HRD tumors likely contributes to enhanced neoantigen quality^23,25,26^.

**Extended Data Fig. 3:**
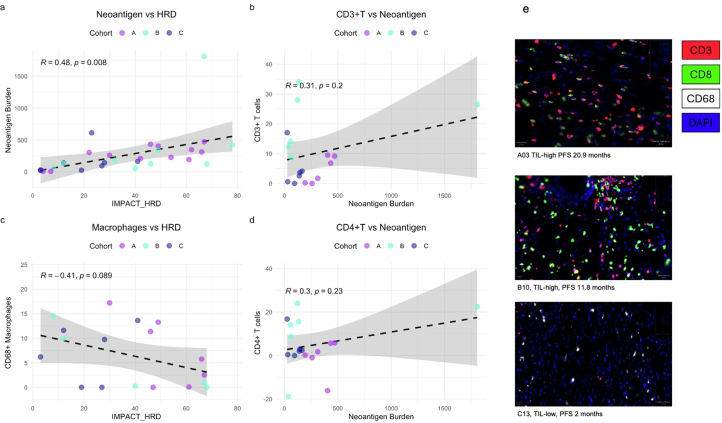
Correlation of immune cell infiltration with IMPACT-HRD score and neoantigen burden in the POLAR cohort (N = 27). Scatter plots display Pearson correlation coefficients (R) and associated p-values between genomic features (IMPACT-HRD score or neoantigen burden) and immune cell infiltration metrics, assessed via multiplex immunofluorescence (mIF). Linear regression lines are shown with 95% confidence intervals (gray shading). Each dot represents a patient sample, color-palette by Cohort A (HRD, purple), B (ncHRD, teal), and C (HRP, navy). (**a**) Neoantigen burden significantly correlates with IMPACT-HRD score (R = 0.48, *p* = 0.008). (**b**) CD3⁺ T cell infiltration shows a modest positive trend with neoantigen burden (R = 0.31, *p* = 0.2). (**c**) CD68⁺ macrophage infiltration trends inversely with IMPACT-HRD score (R = -0.41, *p* = 0.089). (**d**) CD4⁺ T cell infiltration, inferred as CD3⁺CD8⁻, shows a modest positive trend with neoantigen burden (R = 0.30, *p* = 0.23). CD4⁺ T cells were not directly stained and were approximated as CD3⁺CD8⁻ cells. All correlations were evaluated using Pearson’s method. (**e**) Representative multiplex immunofluorescence images from tumors with high (B10) and low (C13) T-cell infiltration. Tumors were stained for CD3 (red), CD8 (green), CD68 (white), and DAPI (blue). **Abbreviations:** WES, whole exome sequencing; HRD, homologous recombination deficiency; ncHRD, non-core HRD; mIF, multiplex immunofluorescence; TMB, tumor mutational burden; OS, overall survival; PFS, progression-free survival. Source data

In parallel, WES-derived tumor mutational burden (TMB) was significantly higher in cohort A compared to cohort C (median: 2.8 mutations per megabase (IQR: 2.2−4.0) versus 1.25 (IQR: 0.50−1.90), *P* = 0.035) (Fig. 3b and Extended Data Fig. 2a). IMPACT-HRD scores were also significantly higher in cohort A compared to cohort C (median: 47 (IQR: 22−61) versus 24 (IQR: 12−37)) (Extended Data Fig. 2a,c). These genomic features were accompanied by greater tumor-infiltrating lymphocyte (TIL) density on hematoxylin and eosin (H&E) (*n* = 38) in cohort A than in cohort C (median: 3.5 (IQR: 3−4) versus 2 (IQR: 2−3), *P* = 0.035) (Extended Data Fig. 2c). Tumors of participants with longer PFS demonstrated more abundance of TILs (Fig. 3c). Across all samples, IMPACT-HRD score positively correlated with predicted neoantigen burden (*R* = 0.48, *P* = 0.008; Extended Data Fig. 3a).

### Neoantigen burden correlates with HRD but not with CD8^+^ T cell infiltration

Using mIF, we quantified more specific immune cells infiltrated across tumors with available neoantigen and IMPACT-HRD score. Among the mIF-available baseline tumors (*n* = 33), CD3^+^CD8^+^ TILs were more abundant in cohort A versus cohort C (*P* < 0.05) and in participants with PFS > 4 months versus PFS 4 months (*P* < 0.05) (Fig. 3d). Although inferred CD4^+^ T cell (CD3^+^CD8^−^) infiltration showed modest positive trends with neoantigen burden (*R* = 0.31, *P* = 0.2 and *R* = 0.30, *P* = 0.23, respectively), CD8^+^ T cells showed no correlation (*P* = 0.95). CD68^+^ macrophage infiltration trended inversely with IMPACT-HRD score (*R* = −0.41, *P* = 0.089). Programmed death ligand 1 (PD-L1) expression and T-cell-to-macrophage ratios did not significantly correlate with IMPACT-HRD score or neoantigen burdens (Extended Data Fig. 3).

## Discussion

PC remains one of the most challenging malignancies to treat, with limited efficacy from ICB in part due to poor immunogenicity and a highly immunosuppressive tumor microenvironment (TME)^29,30^. The POLAR phase 2 trial was designed to evaluate whether combining a PARP inhibitor with PD-1 blockade during a chemotherapy-free maintenance window could yield continued durable clinical benefit in patients with metastatic PC after response to platinum-based chemotherapy. Although the POLAR trial’s prespecified co-primary endpoints, defined by hypothesized alternative rates of 43% for ORR and 77% for 6-month PFS, were not achieved in *BRCA1/BRCA2/PALB2*-mutant cohort A, these thresholds were intentionally set high to guide feasibility and signal strength in a rare population. In retrospect, the design was overly ambitious for a non-registrational study. Nonetheless, a clinically meaningful signal was observed, with durable responses and prolonged survival in a subset of patients, supporting further investigation of this therapeutic approach.

Although the olaparib arm in the Pancreas Olaparib Ongoing (POLO) trial demonstrated a PFS benefit, no OS improvement was observed (hazard ratio = 0.83 (95% CI: 0.56−1.22), *P* = 0.3487), leading to ongoing debate about the role of olaparib monotherapy in PC maintenance^5,6,31^. In POLAR cohort A, the observed ORR was 35% (95% CI: 15−59%) with a 6-month PFS rate of 64% (95% CI: 49−82), median OS of 28 months (95% CI: 12−NR) and 2-year and 3-year OS rates of 56% (95% CI: 41−76%) and 44% (95% CI: 28−69%), respectively, comparing favorably to the historical olaparib monotherapy in the POLO trial (ORR: 23%, 6-month PFS rate: 53%, 2-year OS rate: 37%, 3-year OS rate: 33.9%)^31^.

POLAR was designed to test a new therapeutic paradigm by combining pembrolizumab, an anti-PD-1 ICB, with PARPi—a strategy supported by retrospective clinical data and preclinical evidence. DNA damage repair (DDR) deficiency has been shown to activate innate immune sensing pathways such as cGAS−STING, and emerging evidence suggests that PARP inhibitors can generate CD8^+^ central memory T cell transcriptional and metabolic reprogramming, enhancing the tumor susceptibility to immunotherapy^32–34^. Notably, 42% (*n* = 13/33) of participants in cohort A had non-measurable so-called ‘undefined’ disease at study entry, reflecting deep responses to previous platinum-based chemotherapy. Inclusion of these patients in a post hoc exploratory analysis increased the ORR to 52% (95% CI: 34−69%) (17/33), which could have met the prespecified decision rule (12/33). This further supports the underlying hypothesis that platinum sensitivity is a clinical surrogate of HRD biology—a principle that forms the foundation for both the POLO trial design and the FDA-approved label for olaparib in PC.

Across various cancers, PARP inhibitors combined with immune check blockade (PARPi-ICB) have demonstrated variable activity. In the tumor-agnostic, phase 2 KEYLYNK-007 trial (*n* = 322), durable responses to pembrolizumab plus olaparib were reported among patients with homologous recombination repair (HRR) mutations (for example, *BRCA1/BRCA2*, *PALB2*, *ATM* and *RAD51D*)^35^. Among 15 participants with PC and *BRCA1* and *BRCA2* mutations, four achieved partial responses, which was notable given the inclusion of heavily pretreated and platinum-refractory cases. By contrast, participants in the POLAR trial were enrolled with platinum-sensitive disease, a so-called ‘molecular’ residual disease (mRD) state, a context increasingly recognized as optimal for immune engagement in PC and as necessitated in adjuvant cancer vaccine trials^15–17^.

Additional PARPi-ICB trials in ovarian, breast, endometrial and lung cancers reported modest activity, particularly in HRD-enriched tumors^36–38^. However, optimal selection criteria, including mutational types, timing, TME context and PARP1-selective inhibition, remain unresolved. Cross-trial evidence suggests that the greatest benefit is achieved in core HRD (biallelic *BRCA1, BRCA2* and *PALB2* loss) tumors or HRD-signature-high setting (for example, COSMIC SBS3, GIS, HRDetect and IMPACT-HRD) in immune-permissive contexts^19,20,22,39,40^.

In the present study, the term ‘homologous recombination deficiency (HRD)’ refers to the presence of pathogenic mutations in core HRR genes—specifically, *BRCA1*, *BRCA2* and *PALB2*—rather than functional confirmation via zygosity. Although more precise nomenclature, such as ‘HRR-mutated’, may reflect the underlying genotype, the term ‘HRD’ remains widely used in clinical trials, including POLAR, and regulatory approvals to indicate enrichment for patients with germline *BRCA1, BRCA2* or *PALB2* pathogenic mutations. In PC, validated HRD functional assays remain limited, and tumor tissue constraints often preclude zygosity determination. In the POLAR trial, 18 of 33 participants in cohort A were eligible by germline core HRD gene mutations without available tissue; therefore, only ctDNA (MSK-ACCESS) was available, precluding zygosity assessment. Among the remaining 15 tissue samples, we were able to evaluate zygosity in 13 cases—10 of which showed biallelic loss, consistent with a ‘true’ HRD-high profile. The two indeterminate cases had tumor purity less than 10%. Overall, we were able to confidently determine zygosity in 45% (15/33) of cohort A and in 80% (12/15) of cohort B (Supplementary Table 1). This limitation reflects the marked platinum-responsive nature—hence, low cellularity in cohort A HRD tumors at the time of enrollment.

The biomarker-integrated design of POLAR enabled comprehensive translational analyses. HRD tumors (cohort A) demonstrated significantly higher WES-derived TMB, IMPACT-HRD scores, indel burden and frameshift indel burden compared to homologous recombination proficient (HRP) tumors (cohort C), consistent with greater genomic instability (Extended Data Fig. 2a−c). These molecular features were associated with a trend toward increased predicted neoantigen burden and CD3^+^ T cell infiltration, suggesting enhanced immunogenicity. However, the relationship between antigenicity and immune infiltration remains incompletely understood and is under active investigation. Notably, some HRD tumors with high neoantigen burden displayed limited T cell infiltration, implying that additional barriers, such as impaired antigen presentation or immune exclusion, may restrict immune engagement despite underlying genomic instability (Extended Data Fig. 3b,d).

Immune exclusion in PC is multifactorial. Our findings, consistent with previous reports, indicate that neoantigen presence alone does not guarantee CD8^+^ T cell infiltration^41^. Mechanisms such as cancer-associated fibroblast (CAF)-mediated FoxP3^+^ regulatory T (T_reg_) cell recruitment and structured exclusionary niches^42^, as well as oncogenic *KRAS*^G12D^-driven CD11b^+^ myeloid cell enrichment^43,44^, likely suppress effector immune cell entry. In the present study, HRD tumors had increased CD3^+^ TILs, although no clear correlation was observed between neoantigen burden and CD8^+^ T cell infiltration, reinforcing the role of additional suppressive axes beyond antigen presentation. These results suggest that, although neoantigen recognition is necessary, it is not sufficient for full and robust immune engagement in PC.

Fifteen participants (*n* = 13 from cohort A and *n* = 2 from cohort B) remained on therapy beyond 1 year (PFS > 1 year), whereas only a few in cohort C achieved sustained disease control. This disparate outcome suggests that individual HRD or ncHRD mutations may influence therapeutic sensitivity and underscores the importance of molecular stratification. Moreover, exploratory analysis of ctDNA dynamics suggested that mRD negativity, defined by undetectable or near-undetectable mean VAF, may be associated with durable benefit, highlighting a possible role of mRD as a clinical indicator for future immunotherapy trials.

Our study has limitations. The single-center, multicohort parallel cohort, non-randomized, single-arm design and modest sample size limit generalizability. High rate of sample quality control fails, although expected in PC, limited complete multiomic profiling. Strengths include complete participant follow-up, prospective biospecimen collection and comprehensive integrated genomic and immunologic analyses, providing notable insights for the clinical observations.

Looking ahead, rational combination strategies in biomarker-selected subsets are essential to overcome barriers to effective immune engagement in PC. Beyond PARP, the DDR therapeutic landscape is rapidly evolving with next-generation targets such as PARP1-selective, *POLQ*, *ATR*, *WEE1*, *CHK1*, PARG and tankyrase inhibitors now in clinical development^45^. Integration of DDR-targeted agents with TME-modulating therapies, such as RAS/KRAS inhibitors, myeloid/CD11b modulators or CAF-directed strategies, may enhance neoantigen-cognate T cell infiltration and improve durable responses, particularly in replication stress-high contexts where precision immunotherapy can be most effective. Spatial profiling, T cell receptor tracking, mutational signature analyses associated with neoantigens and functional characterization of these interactions are ongoing within the POLAR trial. Together, these efforts position POLAR as a model for biomarker-integrated precision immunotherapy studies and highlight the central role of HRD, neoantigen landscape and immune contexture in shaping therapeutic outcomes in PC.

## Conclusion

In conclusion, although not meeting the prespecified primary endpoints, the POLAR trial demonstrates that a biomarker-guided maintenance approach combining PARP inhibition with anti-PD-1 ICB is feasible and yields durable clinical benefit in a notable subset of patients with PC. In cohort A, which comprised patients with core HRD (*BRCA1, BRCA2* and *PALB2* mutations), we observed high tumor response rates, durable disease control and encouraging long-term survival. These findings support a model in which DDR-driven genomic instability promotes tumor immunogenicity, acknowledging that immune engagement is ultimately shaped by additional suppressive features within the TME. These results provide a rationale for ongoing randomized studies (for example, SWOG S2001 (NCT04548752)) and future precision immunotherapy trials that integrate DDR status, neoantigen quality and TME remodeling to improve outcomes in patients with PC.

## Methods

### Study design and participants

This was a single-institution, open-label, non-randomized phase 2 trial conducted at Memorial Sloan Kettering Cancer Center (NCT04666740). Participants at least 18 years of age with metastatic PC (including adenosquamous or acinar subtypes) and an ECOG performance status of 0–1 were enrolled into three biomarker-defined cohorts: cohort A, *BRCA1/BRCA2*-mutated or *PALB2*-mutated tumors (core HRD); cohort B, tumors harboring non-core HRR gene mutations; and cohort C, HRP tumors with durable platinum sensitivity. Eligibility required completion of platinum-based induction therapy without radiographic progression prior to enrollment. Key exclusion criteria included more than two previous systemic therapies, previous PARPi or ICB or progression before trial entry.

Participants were enrolled between 11 January 2021 and 7 March 2024; 63 participants were treated. Sex was recorded by self-report for all participants; gender identity beyond sex assigned at birth was not systematically collected. The median age at enrollment was 62 years (range, 53–70). Sex was considered a descriptive variable; the study was not powered to detect sex-specific or gender-specific differences, and no such analyses were prespecified. All participants provided written informed consent. The study was approved by the institutional review board at Memorial Sloan Kettering Cancer Center and was conducted in accordance with the Declaration of Helsinki and Good Clinical Practice guidelines. Participants did not receive financial compensation beyond standard-of-care coverage.

### Treatment and follow-up

All participants received maintenance olaparib (300 mg twice daily) and pembrolizumab administered every 3 weeks for 6 months and then every 6 weeks until disease progression or unacceptable toxicity. Select participants could continue treatment beyond radiographic progression at investigator discretion if clinical benefit was observed. Data cutoff was 25 June 2025.

### Endpoints

For cohort A, the co-primary endpoints were ORR assessed by iRECIST among participants with measurable disease and 6-month PFS rate assessed by RECIST version 1.1 in all treated participants. For cohorts B and C, the primary endpoint was exploratory ORR.

Prespecified secondary endpoints for all cohorts included safety (graded by Common Terminology Criteria for Adverse Events version 5.0), DCR, DoR, PFS and OS. Prespecified exploratory endpoints included ctDNA dynamics, genomic and neoantigen features and immune infiltration metrics.

### Sample size determination and power

Sample size and power were determined separately by cohort. Cohort A employed a prespecified two-stage phase 2 design. An initial stage enrolled 20 participants; if six or more objective responses were observed or if 14 or more participants were alive and progression free at 6 months, an additional 13 participants were accrued (total *n* = 33). The regimen was considered sufficiently active if 12 or more objective responses were observed or if at least 23 of 33 participants were alive and progression free at 6 months.

The design was based on historical maintenance benchmarks in HRD PC (6-month PFS 53%; ORR 23%)^5^. This design provided 81% power to detect a true 6-month PFS rate of ≥77% and 81% power to detect a true ORR of ≥43%. The type I error rates were 0.028 (PFS) and 0.02 (ORR), with overall type I error bounded at 0.05; the composite type II error rate was 0.09. The probability of early termination under the null hypothesis was 0.73, assuming independence between ORR and PFS. Participants without documented response or 6-month PFS status due to loss to follow-up were counted as non-responders/progressors for the composite endpoint.

For cohorts B and C, 15 participants per cohort were enrolled for exploratory purposes to provide descriptive estimates of efficacy and time-to-event outcomes. ORR and DCR were estimated using exact binomial CIs; with 15 participants, DCR could be estimated with an approximate ±25% margin of error at the 95% confidence level. These cohorts were not powered for formal hypothesis testing.

### Radiographic response assessment

Baseline computed tomography imaging was performed within 28 days of enrollment. Imaging occurred every 9 weeks (±7 days) through cycle 10 and then every 12 weeks. Responses were assessed by central blinded radiology review using RECIST version 1.1 (Mint Lesion). ORR analyses included only participants with measurable disease at study entry. An exploratory analysis including participants with non-measurable but evaluable disease (for example, near-complete responses to prior platinum therapy) was also conducted to contextualize clinical benefit.

### Biospecimen collection

Image-guided tumor biopsies were obtained at baseline (T1), on-treatment (cycles 2–3 (T2)) and at progression (T3), when feasible. Samples were formalin-fixed, paraffin-embedded (FFPE) or snap frozen for downstream analyses. Peripheral blood was collected longitudinally for peripheral blood mononuclear cell (PBMC) and ctDNA analyses.

### Research biospecimen collection

Image-guided tumor biopsies were obtained at three timepoints: baseline (T1), on-treatment (cycles 2−3 (T2)) and at progression (T3), when feasible. Samples were FFPE or snap frozen for WES and mIF. Peripheral blood was collected at six timepoints for PBMC and ctDNA analysis (baseline, on-treatment, at cycle 6 (C6), cycle 12 (C12), cycle 18 (C18) and progression).

### Genomic analyses

Tumor and matched normal DNA underwent WES using the institutional pipeline (TEMPO; https://github.com/mskcc/tempo); somatic variants were called with MuTect2. Genomic instability was quantified using the IMPACT-HRD score, derived from allele-specific copy number alterations (FACETS version 0.5.14) and computed with the IMPACT-HRD package. Metrics included TAI, LSTs and LOH, summed to generate an HRD score with consideration of whole-genome duplication.

### Genomic instability and IMPACT-HRD score

IMPACT-HRD quantifies genomic scars associated with HRD by analyzing allele-specific copy number alterations determined with the FACETS algorithm^46^ (version 0.5.14) and computing those genomic scars with the IMPACT-HRD package (https://github.com/danielmuldoon/impact-hrd/). All IMPACT-HRD assessments were completed using R version 4.1.2. In particular, three metrics are evaluated: number of telomeric allelic imbalances (NtAI), LSTs and losses of heterozygosity (HRD-LOH). The overall HRD phenotype is defined as the unweighted sum of these three metrics (HRD-sum), with additional consideration given to whole-genome duplication (WGD) status.

### Neoantigen prediction

Somatic mutations from WES or Memorial Sloan Kettering–Integrated Mutation Profiling of Actionable Cancer Targets (MSK-IMPACT) were analyzed using NetMHCpan version 4.0 to predict major histocompatibility complex (MHC) class I binding affinity. Peptides of 8 to 11 residues with predicted binding affinity of ≤500 nM were considered candidate neoantigens. To account for both quantity and binding strength, a weighted neoantigen burden was computed per sample by summing the inverse binding affinity (1 / IC_50_, where IC_50_ is the half-maximal inhibitory concentration) across all retained peptides. This score emphasizes the contribution of strong binders and was used for sample ranking and correlation analyses.

### Cell-free DNA: MSK-ACCESS

The MSK-ACCESS liquid biopsy test was employed to study ctDNA in the plasma. An in-depth description of this assay was previously published^47^. This test uses hybridization capture and deep sequencing to discover low-frequency somatic changes and pinpoint different kinds of genomic irregularities, such as single-nucleotide polymorphisms, base indels and copy number alterations. The test scrutinizes selected exons and introns from 147 genes, which were previously identified to be defective in human cancers. It is important to note that this assay can detect VAFs as low as 0.1%. The technique uses matched white blood cell sequencing to isolate and filter out germline results from the ctDNA findings and can distinguish mutations linked to clonal hematopoiesis^48^. The New York State Department of Health sanctioned this test for clinical application on 31 May 2019.

Fifty-six samples from 31 patients were studied with MSK-ACCESS, and the results were juxtaposed with those derived from tumors that underwent MSK-IMPACT (29 patients)^49^. The variants (SNVs and indels) resulting from the procedure were manually examined using the Integrated Genomics Viewer (version 0.4.0). For 29 patients, where samples were profiled by both MSK-ACCESS and MSK-IMPACT, variants were genotyped across the shared region between the two assays using GetBaseCounMultiSample (version 1.2.5). VAF was computed from somatic, non-clonal hematopoiesis mutations. Longitudinal VAF dynamics were analyzed when paired timepoints were available.

### Multiplex immunofluoresence

mIF was performed on FFPE tissue from 21 patients using a validated panel: CD3, CD8, CD68, PD-L1 and DAPI. Imaging was conducted on the Vectra Polaris platform. QuPath version 0.5.1 was used for automated quantification. Tumor and stromal compartments were annotated by a pathologist. Marker-positive cells were normalized to stromal cell count. Correlation among mIF markers, TMB and cohort was computed using ggpubr in R (version 4.2.2).

Automated mIF was done using a Leica Bond RX staining system. FFPE tissues from 21 patients were sectioned at 5 μm and baked at 58 °C for 1 hour before loading in the Leica Bond and stained using the following protocol. Samples were dewaxed at 72 °C and treated with ER2 epitope retrieval solution (Leica Biosystems, AR9640) for 20 minutes at 95 °C. A 5-plex PD-L1/CD8/CD68/CD20/CD3 panel was applied sequentially using the following antibodies: PD-L1 (Cell Signaling Technology, clone E1L3N, 1:2,000 dilution); CD8 (Roche Ventana, clone SP57, 1:200 dilution); CD20 (DAKO, clone L26, 1:2,000 dilution); CD68 (DAKO, clone KP1, 1:200 dilution); and CD3 (Roche Ventana, clone 2GV6, 1:2,000 dilution). After incubation with an antibody for 1 hour, Leica Bond Polymer anti-rabbit HRP was applied, followed by the relevant fluorophore (tyramide-conjugated Alexa Fluor dye 488 or 647 (Life Technologies, B40953 and B40958) or tyramide-conjugated CF dye 430, 543 or 594 (Biotium, 96053, 92172 and 92174)) for signal amplification. Epitope retrieval was repeated between rounds of staining to denature antibodies before addition of the next primary antibody. After immunofluorescence staining, samples were counterstained with 5 μg ml^−1^ DAPI (Sigma-Aldrich), rinsed briefly in PBS and mounted in Mowiol 4-88 (Calbiochem). Slides were scanned on a Pannoramic MIDI II whole-slide scanning fluorescence microscope (3DHISTECH) with a ×20/0.8 numerical aperture air objective and visualized in QuPath 0.4.2 (ref. ^50^).

### Statistical analysis

Baseline characteristics were summarized descriptively. OS and PFS were calculated from treatment initiation to death (OS) or first progression/death (PFS) and estimated using Kaplan–Meier methods; median follow-up was estimated using the reverse Kaplan–Meier method. DCR was defined as complete response, partial response or stable disease per RECIST version 1.1. DoR was estimated among responders using Kaplan–Meier methods.

Univariable Cox models (stratified by cohort) assessed associations with OS and PFS; multivariable models included available baseline covariates without variable selection and were similarly stratified. Changes in mean VAF from baseline to on-treatment were compared between participants with PFS ≤ 6 months and participants with PFS > 6 months using the Wilcoxon rank-sum test (no censoring occurred before 6 months). Exploratory analyses used descriptive and non-parametric methods as appropriate. Correlations among immune infiltration, IMPACT-HRD score and neoantigen burden were assessed using Pearson correlation coefficients. Analyses were performed in R version 4.3.2, and tests were two-sided with *P* < 0.05 considered nominally significant.

### Reporting summary

Further information on research design is available in the Nature Portfolio Reporting Summary linked to this article.

## Online content

Any methods, additional references, Nature Portfolio reporting summaries, source data, extended data, supplementary information, acknowledgements, peer review information; details of author contributions and competing interests; and statements of data and code availability are available at 10.1038/s41591-026-04299-5.

## Supplementary information

Supplementary information Supplementary Table 1 and Supplementary Figs. 2 and 3. Supplementary Fig. 2. Cohort A subgroup analysis of PFS and OS by HRD mutations. Supplementary Fig. 3. Cohort B subgroup analysis of PFS and OS by *ATM* mutation status.

Reporting Summary

Supplementary Data Protocol IRB 20-481

Supplementary Table Zygosity calls for cohorts A and B

## Source data

Source Data Figs. 2 and 3 and Source Data Extended Data Figs. 1−3 Statistical source data for Figs. 2 and 3b−d and Extended Data Figs. 1–3

## Data Availability

Genomic and associated clinical data generated in this study are provided in the supplementary information and have been deposited via the Nature Research data deposition system (figshare) and will be publicly accessible upon publication. Raw sequencing data are considered protected health information and are available under controlled access subject to institutional review and execution of a data transfer agreement. Requests for access may be directed to the corresponding authors and will be reviewed within 4 weeks. Source data are provided with this paper.

## Code availability

Processed analysis datasets, annotated mutation files and analysis scripts used for figure generation and statistical analyses are available at https://github.com/WilliamPolar/POLAR-trial-genomic-analysis. A versioned release of the repository is archived with 10.5281/zenodo.18643676
