# Supplementary material for: Pembrolizumab and olaparib in homologous-recombination-deficient metastatic pancreatic cancer: the phase 2 POLAR trial
Source: Nat Med. 2026 Mar 25;32(5):1783–93. doi: 10.1038/s41591-026-04299-5 (PMC13190252; doi:10.1038/s41591-026-04299-5)
Supplement: Supplementary file 1 — Supplementary Table 1 and Supplementary Figs. 2 and 3. Supplementary Fig. 2. Cohort A subgroup analysis of PFS and OS by HRD mutations. Supplementary Fig. 3. Cohort B subgroup analysis of PFS and OS by ATM mutation status. [file 41591_2026_4299_MOESM1_ESM.pdf]

nature medicine

Supplementary information https://doi.org/10.1038/s41591-026-04299-5

Pembrolizumab and olaparib in homologous-recombination-deficient metastatic pancreatic cancer: the phase 2 POLAR trial

In the format provided by the authors and unedited

Supplementary File 1. Protocol IRB 20-481 file (raw file attached)
Supplementary File 2. POLAR_Stat.html file (raw file attached)
Supplementary File 3. Cohort A and B zygosity file (raw file attached)

Supplementary Table 1. Cohort A and B zygosity (file 3)
Supplementary Figure 2. Cohort A subgroup analysis of PFS and OS by HRD mutations

Cohort	POLAR_ID	DDR_gene	Hugo_Symbol	Germline_Somatic_ACCESS	Zygosity	Reversion_T3	note	Tumor_Puri	t_var_freq	t_ref_count	t_alt_count	n_ref_count	[illegible]
A	A01	gBRCA2m	BRCA2	GS	Bilallelic	N	Compound somatic + germline	0.4061	0.7164	171	432	.	.
A	A02	gBRCA2m	BRCA2	GS	Bilallelic	Y	Reversion post-treatment	0.3798	0.6366	149	261	.	.
A	A03	gBRCA2m	BRCA2	cfDNA only	NA	N	ACCESS only						
A	A04	gBRCA2m	BRCA2	cfDNA only	NA	N	ACCESS only						
A	A05	gBRCA2m	BRCA2	GS	Bilallelic	N	Compound somatic + germline	0.2755	0.6625	162	318	.	.
A	A06	gPALB2m	PALB2	cfDNA only	NA	N	ACCESS only						
A	A07	gBRCA2m	BRCA2	GS	Monoallelic	N	Manually reviewed in IGV; tumor VAF 51% (196 fs del, 191 ref)	0.2745	NA	0	0	.	.
A	A08	gBRCA2m	BRCA2	GS	Bilallelic	N	Manually reviewed in IGV; tumor VAF 66% (186 DEL, 95 ref)	0.4105	NA	0	0	.	.
A	A09	gBRCA1m	BRCA1	cfDNA only	NA	N	ACCESS only						
A	A10	gPALB2m	PALB2	GS	Bilallelic	N	Compound somatic + germline	0.7932	0.8005	80	321	.	.
A	A11	gPALB2m	PALB2	cfDNA only	NA	N	ACCESS only						
A	A12	gBRCA2m	BRCA2	cfDNA only	NA	N	ACCESS only						
A	A13	gBRCA2m	BRCA2	Somatic_only	Monoallelic	N	Low purity (pathology estimate 20%) but likely monoallelic (tumor VAF = ~1/2 purity)	0.1800	0.0903	272	27	249	[illegible]
A	A14	gBRCA1m	BRCA1	cfDNA only	NA	N	ACCESS only						
A	A15	gPALB2m	PALB2	GS	Monoallelic	N	Manually reviewed in IGV; tumor VAF 50% (240 DEL, 240 ref)	0.2521	NA	0	0	.	.
A	A16	gBRCA2m	BRCA2	cfDNA only	NA	N	ACCESS only						
A	A17	gBRCA1m	BRCA1	cfDNA only	NA	N	ACCESS only						
A	A18	gBRCA1m	BRCA1	cfDNA only	NA	N	ACCESS only						
A	A19	gPALB2m	PALB2	GS	Bilallelic	N	Compound somatic + germline	0.5556	0.3814	146	90	304	[illegible]
A	A20	gBRCA2m	BRCA2	cfDNA only	NA	N	ACCESS only						
A	A21	gBRCA2m	BRCA2	GS	Bilallelic	Y	Potential reversion mutation in later ACCESS sample	0.2300	0.5170	227	243	.	.
A	A22	gBRCA1m	BRCA1	GS	Indeterminate	N	Low purity (pathology estimate 10%)	0.1910	0.5474	191	231	.	.
A	A23	gBRCA2m	BRCA2	GS	Bilallelic	N	Manually reviewed in IGV; tumor VAF 80% (total count 277, 224 INS)	0.8700	NA				
A	A24	gBRCA2m	BRCA2	GS	Indeterminate	N	Low purity (pathology estimate 10%)	0.3000	0.4914	178	172	.	.
A	A25	gBRCA2m	BRCA2	cfDNA only	NA	N	ACCESS only						
A	A26	gBRCA1m	BRCA1	cfDNA only	NA	N	ACCESS only						
A	A27	gBRCA2m	BRCA2	GS	Bilallelic	N	Compound somatic	0.3000	0.2720	273	102	430	[illegible]
A	A28	gPALB2m	PALB2	cfDNA only	NA	N	ACCESS only						
A	A29	gBRCA1m	BRCA1	cfDNA only	NA	N	ACCESS only						
A	A30	gBRCA1m	BRCA1	cfDNA only	NA	N	ACCESS only						
A	A31	gBRCA1m	BRCA1	GS	Bilallelic	N	Compound somatic + germline	0.3749	0.2056	143	37	465	[illegible]
A	A32	gBRCA2m	BRCA2	cfDNA only	NA	N	ACCESS only						
A	A33	gBRCA2m	BRCA2	cfDNA only	NA	N	ACCESS only						
B	B01	gATMm	ATM	cfDNA only	NA	N	ACCESS only						
B	B02	gATMm	ATM	GS	Bilallelic	N	Compound somatic + germline	0.6676	0.4475	279	226	.	.
B	B03	gATMm	ATM	GS	Bilallelic	N	Compound somatic + germline	0.3907	0.5147	99	105	.	.
B	B04	gCHEK2m	CHEK2	GS	Indeterminate	N	Low purity (pathology estimate 10%)	NA	0.4777	363	332	.	.
B	B05	gATMm	ATM	cfDNA only	NA								
B	B06	gBLMm	BLM	GS	Indeterminate	N	Indeterminate; low normal VAF (41%)	0.4507	0.5088	222	230	.	.
B	B07	gCHEK2m	CHEK2	GS	Bilallelic	N	Manually reviewed in IGV; tumor VAF 100% (166/166)	0.7421	NA	0	0	.	.
B	B08	gFANCCm	FANCC	GS	Bilallelic	N	Compound somatic + germline	0.6283	0.6830	97	209	.	.
B	B09	gATMm	ATM	cfDNA only	NA	N	ACCESS only						
B	B10_1	gATMm_gMU	ATM	GS	Monoallelic	N	Gain of WT	0.3579	0.4085	278	192	.	.
B	B10_2	gATMm_gMU	MUTYH	GS	Indeterminate	N	Low purity (pathology estimate 15%)	0.3579	0.5186	465	501	.	.
B	B11	gCHEK2m	CHEK2	GS	Monoallelic	N	Manually reviewed in IGV; tumor VAF 52% (37 DEL, 34 ref) - low coverage?	0.4796	NA	0	0	.	.
B	B12	gATMm	ATM	GS	Bilallelic	N	Compound somatic + germline	0.8795	0.9032	36	336	.	.
B	B13	gMUTYHm	MUTYH	GS	Indeterminate	N	Low purity (pathology estimate 10%)	0.2000	0.5229	250	274	.	.
B	B14	gATMm	ATM	GS	Indeterminate	N	Low purity	0.1525	NA	0	0	.	.
B	B15	gATMm_gMUT	MUTYH	GS	Indeterminate	N	Low purity	0.1655	NA	0	0	.	.

Supplementary File 3. Cohort A and B zygosity (raw file also attached)

		UV PFS[1]			MV PFS[1]		
**Characteristic**	**N**	**HR**	**95% CI**	**p-value**	**HR**	**95% CI**	**p-value**
Age	63	1.00	0.97, 1.03	0.91	1.00	0.97, 1.03	0.81
SEX	63			0.22			0.12
F		—	—		—	—	
M		1.45	0.80, 2.62		1.77	0.87, 3.61	
Stage at diagnosis	63			0.61			0.79
de novo		—	—		—	—	
METS		1.23	0.55, 2.77		1.13	0.46, 2.76	
Tumor Location	63			0.96			0.14
Body		—	—		—	—	
Head		0.90	0.42, 1.89		0.70	0.29, 1.69	
Tail		0.93	0.42, 2.07		0.36	0.13, 1.01	
ECOG	63			0.35			0.79
ECOG 0		—	—		—	—	
ECOG 1		1.31	0.74, 2.34		1.09	0.58, 2.04	
Months of prior platinum therapy	63	0.93	0.83, 1.05	0.24	0.88	0.72, 1.07	0.20
ZYGOSITY	27			0.64			
Biallelic		—	—				
Indeterminate		0.58	0.16, 2.17				
Monoallelic		1.12	0.34, 3.67				
Baseline CA 19-9 (per 100 unit)	58	1.02	1.01, 1.02	<0.001	1.02	1.01, 1.03	<0.001

[1]Stratified Cox regression model was used

		UV OS[1]			MV OS[1]		
**Characteristic**	**N**	**HR**	**95% CI**	**p-value**	**HR**	**95% CI**	**p-value**
Age	63	1.00	0.97, 1.03	0.89	1.00	0.97, 1.04	0.88
SEX	63			0.061			0.13
F		—	—		—	—	
M		1.88	0.97, 3.62		1.85	0.84, 4.08	
Stage at diagnosis	63			0.90			0.79
de novo		—	—		—	—	
METS		1.06	0.43, 2.63		1.15	0.41, 3.21	
Tumor Location	63			0.23			0.054
Body		—	—		—	—	
Head		1.83	0.82, 4.11		1.54	0.60, 3.97	
Tail		1.10	0.45, 2.69		0.43	0.13, 1.37	
ECOG	63			0.13			0.31
ECOG 0		—	—		—	—	
ECOG 1		1.63	0.87, 3.06		1.42	0.72, 2.77	
Months of prior platinum therapy	63	0.95	0.85, 1.06	0.39	0.89	0.73, 1.09	0.27
ZYGOSITY	27			0.14			
Biallelic		—	—				
Indeterminate		0.23	0.05, 1.02				
Monoallelic		0.80	0.23, 2.77				
Baseline CA 19-9 (per 100 unit)	58	1.01	1.00, 1.02	0.020	1.02	1.01, 1.03	0.002

[1]Stratified Cox regression model was used

Abbreviations: CI = Confidence Interval, HR = Hazard Ratio; UV, univariable; MV, multivariable

Supplementary Table 1. Univariable and multivariable analysis with PFS[1] and OS[1]

a.

Characteristic	N = 33[1]
MUTATION	
BRCA1	9 (27%)
BRCA2	18 (55%)
PALB2	6 (18%)

[1] n (%)

b.

Characteristic	Median PFS months (95%CI)
MUTATION	
BRCA1	6.1 (4.1, —)
BRCA2	9.9 (3.6, —)
PALB2	12 (6.2, —)

c.

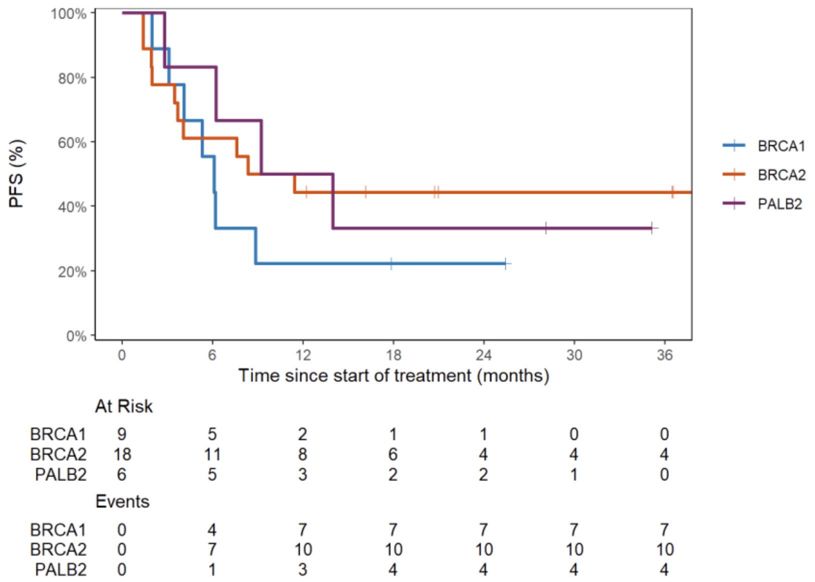

d.

Characteristic	Median Survival, months (95%CI)
MUTATION	
BRCA1	18 (12, —)
BRCA2	28 (9.9, —)
PALB2	27 (11, —)

Characteristic	Time 12	Time 24
MUTATION		
BRCA1	67% (42%, 100%)	42% (18%, 94%)
BRCA2	59% (40%, 88%)	59% (40%, 88%)
PALB2	67% (38%, 100%)	67% (38%, 100%)

e.

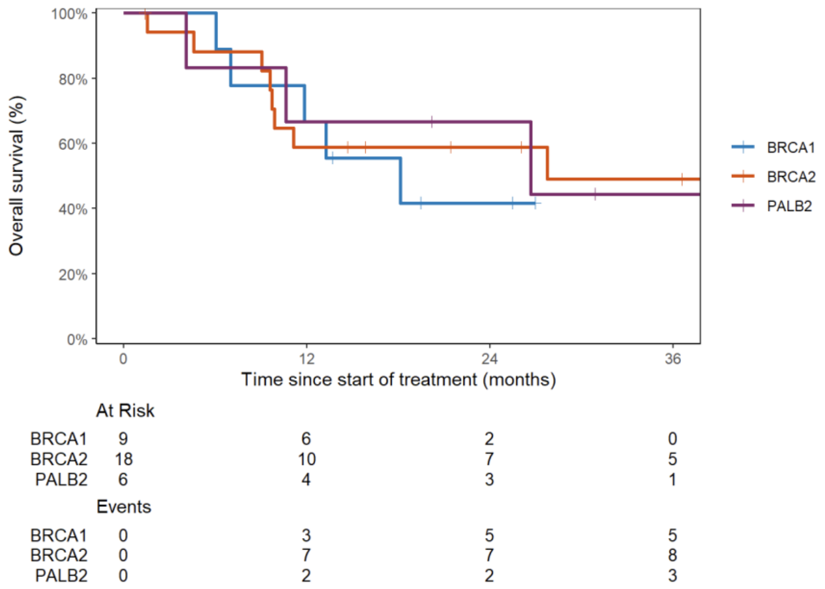

Supplementary Figure 2. Cohort A subgroup analysis of PFS and OS by HRD mutations

(a) Distribution of HRD mutations in Cohort A (N = 33), showing the proportion of *BRCA1*, *BRCA2*, and *PALB2* mutations.
(b) Median PFS in months by mutation type with 95% confidence intervals (CI).
(c) Kaplan–Meier curves of PFS stratified by *BRCA1* (blue), *BRCA2* (red), and *PALB2* (purple).
(d) Median OS in months and landmark survival rates at 12 and 24 months by mutation type.
(e) Kaplan–Meier curves of OS stratified by *BRCA1* (blue), *BRCA2* (red), and *PALB2* (purple).
Censoring is indicated by tick marks. Survival estimates are based on investigator-assessed outcomes. Numbers at risk and number of events are displayed as tick in each Kaplan–Meier plot.

Abbreviations: OS, overall survival; PFS, progression-free survival

a.

Characteristic	N = 15[1]
ATM	9 (60%)

[1] n (%)

b.

Characteristic	Median PFS months (95%CI)
ATM	
0	6.5 (4.0, —)
1	4.8 (2.0, —)

c.

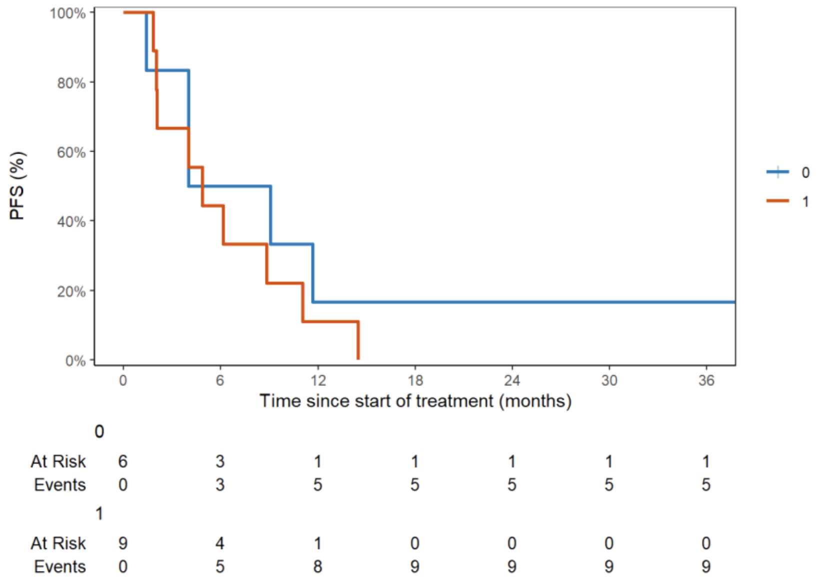

d.

Characteristic	Median Survival, months (95%CI)
ATM	
0	14 (12, —)
1	18 (15, —)

e.

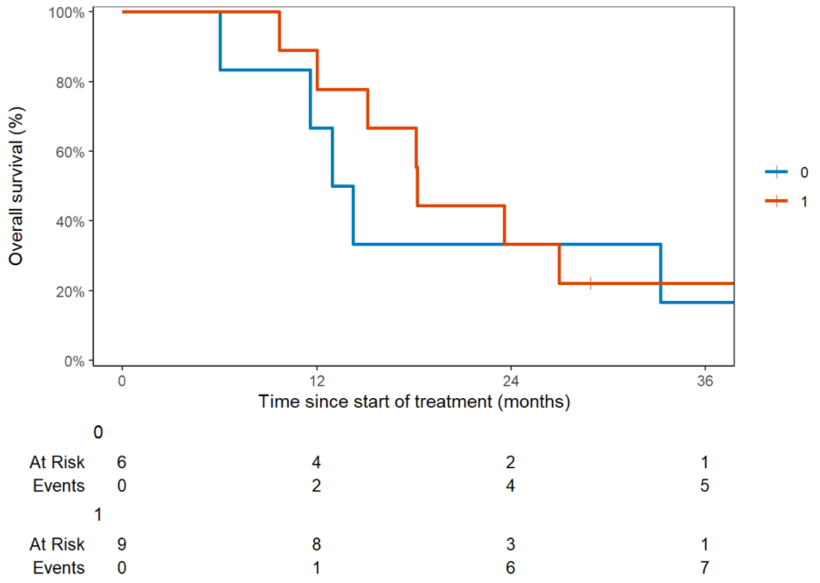

Supplementary Figure 3. Cohort B subgroup analysis of PFS and OS by *ATM* mutation status.
(a) Distribution of ATM or other ncHRD mutations in Cohort B (N = 15), showing 60% (9/15) of patients harbored ATM alterations.
(b) Median PFS by *ATM* mutation status with 95% confidence intervals (CI).
(c) Kaplan–Meier curve of PFS of participants stratified by *ATM* (1, red) or other ncHRD (0, blue).
(d) Median OS by *ATM* mutation status with 95% CI.
(e) Kaplan–Meier curve of OS of participants stratified by *ATM* (1, red) or other ncHRD (0, blue).
Numbers at risk and number of events are shown below each curve. Survival outcomes were investigator-assessed. No statistically significant differences were observed, but trends are noted.

Abbreviations: OS, overall survival; PFS, progression-free survival
