# Supplementary material for: Pembrolizumab and olaparib in homologous-recombination-deficient metastatic pancreatic cancer: the phase 2 POLAR trial
Source: Nat Med. 2026 Mar 25;32(5):1783–93. doi: 10.1038/s41591-026-04299-5 (PMC13190252; doi:10.1038/s41591-026-04299-5)
Supplement: Supplementary file 2 — Reporting Summary [file 41591_2026_4299_MOESM2_ESM.pdf]

nature portfolio

Corresponding author(s): Wungki Park, MD, MS and Eileen M. O'Reilly, MD, FASCO

Last updated by author(s): Jan 19, 2026

Reporting Summary

Nature Portfolio wishes to improve the reproducibility of the work that we publish. This form provides structure for consistency and transparency in reporting. For further information on Nature Portfolio policies, see our Editorial Policies and the Editorial Policy Checklist.

Statistics

For all statistical analyses, confirm that the following items are present in the figure legend, table legend, main text, or Methods section.

n/a	Confirmed	
☐	☒	The exact sample size (*n*) for each experimental group/condition, given as a discrete number and unit of measurement
☐	☒	A statement on whether measurements were taken from distinct samples or whether the same sample was measured repeatedly
☐	☒	The statistical test(s) used AND whether they are one- or two-sided<br>*Only common tests should be described solely by name; describe more complex techniques in the Methods section.*
☐	☒	A description of all covariates tested
☐	☒	A description of any assumptions or corrections, such as tests of normality and adjustment for multiple comparisons
☐	☒	A full description of the statistical parameters including central tendency (e.g. means) or other basic estimates (e.g. regression coefficient) AND variation (e.g. standard deviation) or associated estimates of uncertainty (e.g. confidence intervals)
☐	☒	For null hypothesis testing, the test statistic (e.g. *F*, *t*, *r*) with confidence intervals, effect sizes, degrees of freedom and *P* value noted<br>*Give P values as exact values whenever suitable.*
☒	☐	For Bayesian analysis, information on the choice of priors and Markov chain Monte Carlo settings
☒	☐	For hierarchical and complex designs, identification of the appropriate level for tests and full reporting of outcomes
☐	☒	Estimates of effect sizes (e.g. Cohen's *d*, Pearson's *r*), indicating how they were calculated

Our web collection on statistics for biologists contains articles on many of the points above.

Software and code

Policy information about availability of computer code

Data collection	Clinical data were collected using institutional electronic medical record systems.
Data analysis	Analyses were performed in R (v4.2.2), using packages including survival, survminer, statsmodels, and custom scripts.

For manuscripts utilizing custom algorithms or software that are central to the research but not yet described in published literature, software must be made available to editors and reviewers. We strongly encourage code deposition in a community repository (e.g. GitHub). See the Nature Portfolio guidelines for submitting code & software for further information.

Data

Policy information about availability of data

All manuscripts must include a data availability statement. This statement should provide the following information, where applicable:

- Accession codes, unique identifiers, or web links for publicly available datasets
- A description of any restrictions on data availability
- For clinical datasets or third party data, please ensure that the statement adheres to our policy

All other data supporting the findings are available within the article and its supplementary information.

Research involving human participants, their data, or biological material

Policy information about studies with human participants or human data. See also policy information about sex, gender (identity/presentation), and sexual orientation and race, ethnicity and racism.

Reporting on sex and gender	Both male and female participants were enrolled in the POLAR trial. Sex was recorded as part of the demographic data collection and is reported in the baseline characteristics table. Analyses were not stratified by sex because the study was not powered for sex-based comparisons, and there was no a priori hypothesis regarding sex as an effect modifier for the primary or secondary endpoints.
Reporting on race, ethnicity, or other socially relevant groupings	Race and ethnicity were collected as part of standard clinical and demographic data. These variables are reported descriptively in the baseline characteristics table. Analyses were not stratified by race or ethnicity because the study was not powered for subgroup analyses, and there was no pre-specified hypothesis related to these variables.
Population characteristics	Participants had metastatic pancreatic ductal adenocarcinoma with or without homologous recombination deficiency (HRD), enrolled between December 2020 to February 2024
Recruitment	Patients were enrolled at Memorial Sloan Kettering Cancer Center and collaborating institutions. Eligible patients were identified through clinic visits and institutional databases; no financial incentives were provided.
Ethics oversight	The study was approved by the Memorial Sloan Kettering Cancer Center Institutional Review Board 20-481

Note that full information on the approval of the study protocol must also be provided in the manuscript.

Field-specific reporting

Please select the one below that is the best fit for your research. If you are not sure, read the appropriate sections before making your selection.

☒ Life sciences ☐ Behavioural & social sciences ☐ Ecological, evolutionary & environmental sciences

For a reference copy of the document with all sections, see nature.com/documents/nr-reporting-summary-flat.pdf

Life sciences study design

All studies must disclose on these points even when the disclosure is negative.

Sample size	The POLAR trial used a two-stage design for Cohort A with co-primary endpoints of objective response rate (ORR) by iRECIST and 6-month progression-free survival (PFS) rate by RECIST v1.1. In stage 1, 20 patients were accrued; if ≥14 patients were progression-free at 6 months or ≥6 responses were observed, an additional 13 patients would be enrolled (total n=33). This design provided 81% power to detect a true 6-month PFS rate of 77% (vs. 50% historical) and 81% power to detect a true ORR of 43% (vs. 20% historical), with an overall type I error bounded at 0.05 and an early termination probability of 0.73. Cohorts B and C were exploratory, each enrolling 15 patients.
Data exclusions	One patient (C04) consented but did not start treatment due to rapid disease progression; this patient was excluded from all efficacy and safety analyses. As a result, patient C16 is the last participant in the dataset rather than C15. For molecular analyses (WES, RNA-seq, mIF), samples failing pre-specified quality control metrics were excluded; these QC criteria are detailed in the Methods section.
Replication	All laboratory and computational analyses were performed once per sample using validated protocols and pipelines. No experimental replication was required, but independent validation of key findings was performed where possible using complementary datasets or orthogonal assays, as described in the Methods.
Randomization	This was not a randomized trial.
Blinding	There was no blinding. This is not a randomized trial.

Reporting for specific materials, systems and methods

We require information from authors about some types of materials, experimental systems and methods used in many studies. Here, indicate whether each material, system or method listed is relevant to your study. If you are not sure if a list item applies to your research, read the appropriate section before selecting a response.

Materials & experimental systems

n/a	Involved in the study
☐	☒ Antibodies
☒	☐ Eukaryotic cell lines
☒	☐ Palaeontology and archaeology
☒	☐ Animals and other organisms
☐	☒ Clinical data
☒	☐ Dual use research of concern
☒	☐ Plants

Methods

n/a	Involved in the study
☒	☐ ChIP-seq
☒	☐ Flow cytometry
☒	☐ MRI-based neuroimaging

Antibodies

Antibodies used

Multiplex immunofluorescence was performed using the following primary antibodies: anti–PD-L1 rabbit monoclonal antibody (clone E1L3N, Cell Signaling Technology, lot 7, catalog #13684), anti–CD8 rabbit monoclonal antibody (clone SP57, Ventana, multiple lots, catalog #5937248001), anti–CD20 mouse monoclonal antibody (clone L26, DAKO, lot 64779, catalog #M075501-2), anti–CD68 mouse monoclonal antibody (clone KP1, DAKO, lot 60443, catalog #IR60961-2), and anti–CD3 rabbit monoclonal antibody (clone 2GV6, Ventana, multiple lots, catalog #5278422001).

Validation

All antibodies were validated first by the commercial vendor through IHC, then by the Molecular Cytology Core Facility through IHC on a series of internal control tissues. IF staining was validated on positive control tissues first in single-plex staining, then in multiplex, to ensure specificity and consistency before being run on experimental tissue samples. In cases where multiple lots were used, lot-lot variation was tested for on control tissues prior to staining of experimental samples.

Clinical data

Policy information about clinical studies

All manuscripts should comply with the ICMJE guidelines for publication of clinical research and a completed CONSORT checklist must be included with all submissions.

Clinical trial registration

ClinicalTrials.gov identifier: NCT04666740

Study protocol

IRB 20-481 protocol is attached as the Supplementary 1

Data collection

The POLAR trial was conducted at Memorial Sloan Kettering Cancer Center and collaborating sites. Eligible patients with metastatic pancreatic ductal adenocarcinoma were recruited between December 2020 and February 2024. Clinical data were collected prospectively at scheduled trial visits using institutional electronic medical record systems and trial-specific case report forms. Translational biospecimens (tumor tissue, blood/plasma) were collected at baseline and, when applicable, at progression, and were processed for whole-exome sequencing (WES), RNA-seq, single-nucleus RNA-seq (snRNA-seq), circulating tumor DNA (cfDNA) sequencing, and multiplex immunofluorescence (mIF).

Outcomes

The co-primary endpoints for Cohort A were objective response rate (ORR) by RECIST v1.1 and 6-month progression-free survival (PFS) rate. Secondary endpoints included PFS from consent date, disease control rate (DCR), overall survival (OS), safety/tolerability, and exploratory translational endpoints evaluating genomic instability, homologous recombination deficiency (HRD) status, neoantigen burden, and tumor immune microenvironment features. ORR and DCR were calculated using the binomial distribution with exact 95% confidence intervals; time-to-event endpoints were estimated using Kaplan–Meier methodology.

Plants

Seed stocks

Report on the source of all seed stocks or other plant material used. If applicable, state the seed stock centre and catalogue number. If plant specimens were collected from the field, describe the collection location, date and sampling procedures.

Novel plant genotypes

Describe the methods by which all novel plant genotypes were produced. This includes those generated by transgenic approaches, gene editing, chemical/radiation-based mutagenesis and hybridization. For transgenic lines, describe the transformation method, the number of independent lines analyzed and the generation upon which experiments were performed. For gene-edited lines, describe the editor used, the endogenous sequence targeted for editing, the targeting guide RNA sequence (if applicable) and how the editor was applied.

Authentication

Describe any authentication procedures for each seed stock used or novel genotype generated. Describe any experiments used to assess the effect of a mutation and, where applicable, how potential secondary effects (e.g. second site T-DNA insertions, mosiacism, off-target gene editing) were examined.
