# Supplementary material for: Pembrolizumab and olaparib in homologous-recombination-deficient metastatic pancreatic cancer: the phase 2 POLAR trial
Source: Nat Med. 2026 Mar 25;32(5):1783–93. doi: 10.1038/s41591-026-04299-5 (PMC13190252; doi:10.1038/s41591-026-04299-5)
Supplement: Supplementary file 3 — Protocol IRB 20-481 [file 41591_2026_4299_MOESM3_ESM.pdf]

MSK PROTOCOL COVER SHEET

A Phase 2 Trial to Evaluate the Safety and Antitumor Activity of Pembrolizumab and OLApaRib (POLAR) Maintenance for Patients with Metastatic Pancreatic Ductal Adenocarcinoma and Homologous Recombination Deficiency and/or Exceptional Treatment Response to Platinum-Based Therapy

Principal Investigator/Department: Wungki Park, MD/Department Of Medicine/Gastrointestinal Onc

Memorial Sloan Kettering Cancer Center
1275 York Avenue
New York, New York 10065

Table of Contents

1.0 PROTOCOL SUMMARY AND/OR SCHEMA 4

Figure 1. Schema for genetic and response eligibility criteria

2.0 OBJECTIVES AND SCIENTIFIC AIMS 6

3.0 BACKGROUND AND RATIONALE 7

3.1 Background 7

3.2 Pharmaceutical and Therapeutic Background 8

3.3 Rationale for the Trial and Selected Population 9

4.0 OVERVIEW OF STUDY DESIGN/INTERVENTION 12

4.1 Design 13

Figure 2. Oncoprint for HRD from MSK
Figure 3. Treatment and Biospecimen Schema

4.2 Intervention 15

4.3 Biomarker Assemssments 16

5.0 THERAPEUTIC/DIAGNOSTIC AGENTS & NON-THERAPEUTIC ASSESSMENTS 18

5.1 Pembrolizumab

5.2 Olaparib

6.0 CRITERIA FOR PARTICIPANT ELIGIBILITY 19

6.1 Participant Inclusion Criteria 19

6.2 Participant Exclusion Criteria 21

7.0 RECRUITMENT PLAN 23

7.1 Research Participant Registration 23

7.2 Randomization 24

8.0 INFORMED CONSENT PROCEDURES 24

9.0 PRE-TREATMENT/INTERVENTION 24

Table 1. Standard-of-care and research lood draws

10.0 TREATMENT/INTERVENTION PLAN 26

10.1 Administration

Table 2. Trial Treatment (POLAR)

10.2 Timing of Dose Administration 27

10.3 Dose Modification and toxicity management for immune-related AEs associated with pembrolizumab 28

Table 3 Dose modification and toxicity management guidelines for immune-related AEs associated with pembrolizumab

Table 4. Pembrolizumab infusion reaction dose modification and treatment guidelines

10.4 Rescue Medications & Supportive Care for pembrolizumab 33

10.5 Dose Modification and toxicity management for AEs associated with olaparib **....................33**

Table 5. Dose reductions for olaparib to manage adverse events

Table 6. Dose reduction for olaparib if patient develops moderate renal impairment

10.6 Management of adverse events for <u>AEs associated with olaparib</u>

10.6.1 Management of hematological toxicity

10.6.1.1 Management of anemia

Table 8. Management of anemia

10.6.1.2 Management of neutropenia, leukopenia and thrombocytopenia

Table 9. Management of neutropenia, leukopenia and thrombocytopenia

10.6.1.1 Management of prolonged Hematological Toxicities while on olaparib

10.6.2 Management of Non-Hematological Toxicity

10.6.3 Management of new or worsening pulmonary symptom

10.6.4 Management of nausea and vomiting

10.6.5 Interruptions for intercurrent non-toxicity related events

10.6.6 Renal impairment

Management of adverse events....................34

11.0 EVALUATION DURING TREATMENT/INTERVENTION 38

12.0 CRITERIA FOR REMOVAL FROM STUDY 44

13.0 CRITERIA FOR OUTCOME ASSESSMENT AND ENDPOINT EVALUABILITY 45

14.0 BIOSTATISTICS 46

15.0 TOXICITIES/RISKS/SIDE EFFECTS 48

15.1 Serious Adverse Event (SAE) Reporting 52

16.0 PROTECTION OF HUMAN PARTICIPANTS 53

16.1 Privacy....53

16.2 Data Management 53

16.3 Quality Assurance 53

16.4 Data and Safety Monitoring 54

17.0 REFERENCES....54

18.0 APPENDICES....57

Appendix 1: ECOG Performance Status....57

Appendix 2: Common Terminology Criteria for Adverse Events V5.0 (CTCAE) 57

Appendix 3: Appendix 1: Biomarker Management Plan 58

1.0 PROTOCOL SUMMARY AND/OR SCHEMA

This is a phase 2, open-label, non-randomized study evaluating the novel combination of pembrolizumab and olaparib (POLAR) for maintenance therapy in three cohorts of metastatic PDAC patients with homologous recombination deficiency (HRD) in the first-line or second-line on-platinum setting; two cohorts are genomically enriched and one cohort is identified by platinum sensitivity. The primary endpoint of this study is to evaluate the co-primary endpoints using either 6-month progression-free survival (PFS) rate and objective response rate (ORR) of this combination. The secondary endpoints of this study are overall survival (OS), progression-free survival (PFS), disease control rate (DCR), CA 19-9, and CEA response. Statistically powered analysis of co-primary endpoints will be assessed in Cohort A only and other clinical endpoints will be descriptive in Cohort B and C. Exploratory scientific correlates include assessments of HRD genetic mutational signatures, neoantigens, immunogenic tumor microenvironment, and clonal evolutionary dynamics to study heterogeneity and resistance mechanism to POLAR.

Patients will be assigned to cohorts. Cohorts will be defined as following by CLIA-approved NGS or MSK-IMPACT Part A or C:

- **Cohort A**: Patients with either pathogenic germline or somatic alterations of 3 core homologous recombination-genes (HR-genes) - (*BRCA1/2, or PALB2*) who have stable or responding disease on first-line or second-line platinum therapy in two consecutive imaging assessments over at least 4 months or 16 weeks are eligible for inclusion in Cohort A.

- **Cohort B**: Patients with either pathogenic somatic or germline non-core 15 HR-gene alterations (*ATM, BAP1, BARD1, BLM, BRIP1, CHEK2, FAM175A, FANCA, FANCC, MUTYH, NBN, RAD50, RAD51, RAD51C, RTEL1*) who have stable or responding disease on first-line or second-line platinum therapy in two consecutive imaging assessments over at least 4 months are eligible for inclusion in Cohort B.

MSK IRB#: 20-481 A(11), Approval Date: 11-Jun-2024

- **Cohort C**: Patients without any of the above HR-gene alterations included in Cohort A and B who have platinum-sensitivity, which is defined as durable response of either a partial response (PR) or complete response (CR) during at least 6 months on platinum-based therapy. Variants of unknown significance of candidate HR-genes from Cohort A or B will be eligible for **Cohort C** if they meet the partial response to platinum criterion.

All the participating patients will receive the combination of pembrolizumab 200 mg IV every 3 week and olaparib 300 mg twice day orally daily continuously (POLAR) as a maintenance therapy. After the first 6 months (9 cycles), on C10D1 Pembrolizumab 400 mg IV every 6 week plus olaparib 300 mg twice a day orally will be continued. Per treating investigator's discretion, patients may remain at Pembrolizumab 200 mg IV every 3 weeks after the first 9 cycles. Additionally, to accomodate patients traveling far distances, if, per treating investigator, after the first or second imaging, there are no toxicities, patient's dosing of Pembrolizumab may change to 400 mg IV every 6 weeks. All cycles will be 21-days (3 weeks) in length.

Figure 1. Schema for Genetic and Response Eligibility Criteria:

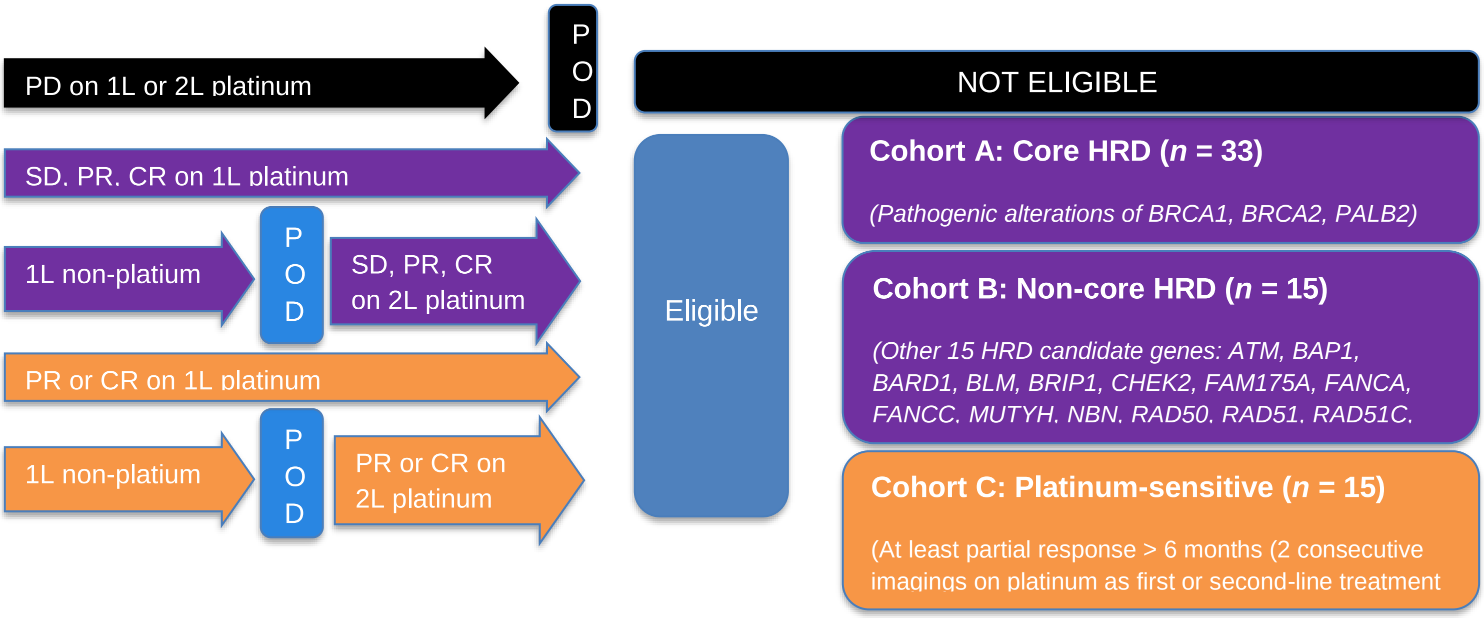

Number of trial participants	63 (Cohort A: 33, Cohort B: 15, Cohort C: 15)
Estimated enrolment period	3 years
Estimated duration of trial	7.5 years
Duration of Participation	4 years

Estimated average length of treatment per participant	4 months

2.0 OBJECTIVES AND SCIENTIFIC AIMS

Primary Objective:

- For Cohort A: To evaluate the activity of pembrolizumab and olaparib (POLAR) combination by co-primary endpoints using either radiographic 6-month PFS rate by RECIST v1.1 and ORR using iRECIST in metastatic pancreas adenocarcinoma (PDAC) patients with core HR-gene mutations.
- For Cohort B and C: To exploratively evaluate the activity of POLAR combination by radiographic ORR using RECIST 1.1.

Secondary Objectives:

- To assess the safety of POLAR by grading adverse events (AE) using CTCAE v. 5.0.
- To assess activity of POLAR by measuring disease control rate (DCR) by both RECIST v1.1 and iRECIST, best overall response rate (BOR), progression-free survival (PFS) by both RECIST v1.1 and iRECIST, overall survival (OS), CEA, and CA 19-9 response. Progression-free survival (PFS) will be analyzed from both the consent date and treatment start date (C1D1) (Reference study POLO defined PFS from the time of randomization not from C1D1, Golan 2019 NEJM).

Exploratory Objectives:

- To explore genetic and transcriptomic signatures for POLAR sensitivity and resistance from HRD Cohort A and B as well as from the platinum-sensitivity Cohort C.
- To describe neoantigen induction, the immunogenic tumor microenvironment (TME), and describe putative immunomodulatory targets from each cohort.
- To explore resistance mechanisms and the role of spatiotemporal heterogeneity by evaluating the tumor samples and circulating tumor DNA (MSK-ACCESS)
- To develop a biorepository of organoids and prospectively evaluate organoids concurrently with the trial in select patients.
- To assess second progression-free survival (PFS2) from time of subsequent systemic treatment start post-POLAR to progression on that treatment.
- To assess duration of the first-line or second-line treatment prior to POLAR treatment.
- To assess genetic signatures and compare clinical outcomes (ORR, PFS, OS) for patients with PDAC harboring specific HRD-gene mutations such as BRCA1, BRCA2, PALB2, ATM, and no HRD-gene mutation.
- To assess the association of other non-HRD gene mutations (such as KRAS allelic imbalance, TP53, SMAD4, CDKN2A homozygous deletion) with the clinical outcomes.
- Evaluate reversion mutation, zygosity, plasticity, and aneuploidy from tumor and circulating tumor DNA.

- To assess the role of maintenance treatment with minimally residual disease by the presence of circulating tumor DNA and CA 19-9 and its trend prior to treatment at time points prior to consent and between the consent and start date.

3.0 BACKGROUND AND RATIONALE

3.1 Background

Pembrolizumab is a potent humanized immunoglobulin G4 (IgG4) monoclonal antibody (mAb) with high specificity of binding to the programmed cell death 1 (PD-1) receptor, thus inhibiting its interaction with programmed cell death ligand 1 (PD-L1) and programmed cell death ligand 2 (PD-L2). Pembrolizumab has an acceptable safety profile and is in clinical development as an intravenous (IV) immunotherapy for advanced malignancies. Keytruda® (pembrolizumab) is Food Drug Administration (FDA)-approved for treatment of patients across a number of indications because of its mechanism of action to bind the PD-1 receptor on the T cell. However, the efficacy of a single-agent pembrolizumab and ipilimumab as well as combination PD-L1 and cytotoxic T-lymphocyte associated protein-4 (CTLA-4) inhibitors in all-comers pancreatic ductal adenocarcinoma has been disappointing [1-3].

Olaparib is a potent polyadenosine 5'diphosphoribose [poly-(ADP) ribose)] polymerization (PARP) inhibitor (PARP-1, -2 and -3) that has been tested as an oral therapy both as a monotherapy (including maintenance) or a combination [4-7]. PARP inhibition is a novel approach to targeting tumors with deficiencies in DNA repair mechanisms. PARP enzymes are essential for repairing DNA single strand breaks (SSBs). Inhibiting PARPs leads to the persistence of SSBs, which are then converted to DNA double strand breaks (DSBs) during the process of DNA replication.

During the process of cell division, DSBs can be efficiently repaired in normal cells by homologous recombination repair. Tumors with homologous recombination deficiencies (HRD), such as ovarian, breast, and pancreatic cancers in patients with germline *BRCA*1/2 (*gBRCA*1/2) mutations, cannot accurately repair the DNA damage, which may become lethal to cells as it accumulates. In such tumor types, Olaparib has shown the efficacy at less toxicity compared with currently available chemotherapy regimens.[8]

Germline *BRCA1* and *BRCA2* defective tumors are intrinsically sensitive to PARP inhibitors, both in tumor models in vivo and in the clinic [9,10]. The mechanism of action for olaparib results from the trapping of inactive PARP onto the single-strand breaks preventing their repair [11,12]. Persistence of SSBs during DNA replication results in their conversion into the more serious DNA DSBs that would normally be repaired by homologous repair. Olaparib has been shown to inhibit selected tumor cell lines in vitro and in xenograft and primary explant models as well as in genetic *BRCA* knockout models, either as a stand-alone treatment or in combination with established chemotherapies. In a phase II pancancer study, PDAC tumors associated with germline *BRCA1/2* mutations showed response to olaparib [8].

Identifying molecular subgroups has provided significant insights into genomic drivers for PDAC.[13-16] Real-time genomic profiling of germline and somatic alterations of *BRCA1, BRCA2, PALB2,* and other significant genes in PDAC patients has been well-demonstrated clinically using FDA-approved MSK-IMPACT profiling [13,17]. At a larger scale, our group has also shown the lineage dependence of carcinogenesis from *BRCA1* and *BRCA2* genes in certain cancers and their preferential response to DNA-damage-response (DDR)-targeted therapies including platinum and PARPi [18]. Beyond a single-agent PARPi and platinum-based chemotherapies, our group have successfully evaluated a combination of veliparib, gemcitabine, and cisplatin in a prospective randomized phase II trial at MSK, where *BRCA1/2* and *PALB2* mutated patients all had very durable response and favored PFS and OS compared to historical PDAC population [19-21]. The patients from the study arm who received gemcitabine, cisplatin, and veliparib had median overall survival of 16.4 months (95% CI, 11.7 to 23.4 months; P =0.6) which was not significantly different compared to the control arm: 15.5 months (95% CI, 12.2 to 24.3 months). However, this outcome is notable compared to the historically the best median overall survival available from published prospective randomized clinical trial 11.1 (95% CI, 9.0 to 13.1) months from FOLFIRINOX[22].

In a recent international, multi-center, randomized first-line maintenance trial entitled POLO for which MSK played a major role, metastatic PDAC patients with a germline *BRCA*1/2 mutation who were sensitive to platinum-based treatment (>= 4 months) were treated either with placebo or with olaparib 300 mg BID maintenance [7]. Olaparib-treated patients had a significantly longer progression-free survival in this pivotal trial. Based on these data olaparib was FDA approved in late 2019 in the maintenance setting for germline *BRCA1/2* pancreas cancer in the maintenance setting. This is the first positive biomarker-selected phase 3 study in metastatic PDAC and offers a new paradigm beyond cytotoxic therapy for patients with PDAC.

However, the POLO trial did not evaluate somatic *BRCA* genes or other HR-genes. Recently, our group has performed a comprehensive clinical outcome and genomic analysis for patients (n=262) with advanced stage PDAC for a broader spectrum beyond *BRCA* genes [23]. In this analysis, we demonstrated that germline or somatic pathogenic alterations of 17 HR-genes, their mutational status, zygosity, and HRD gene signatures that detect signature 3 and genomic instability - large-scale state transition (LST) were associated with superior clinical outcome on platinum-based therapy, warranting a further investigation of DDR-targeted therapy and immunotherapy in these groups [24].

3.2 Pharmaceutical and Therapeutic Background

The importance of intact immune surveillance function in controlling outgrowth of neoplastic transformations has been known for decades [25]. Accumulating evidence shows a correlation between tumor-infiltrating lymphocytes, particularly, the presence of CD8+ T-cells and the ratio of CD8+ effector T-cells/FoxP3+ regulatory T-cells (T-regs) correlates with improved prognosis and long-term survival in solid malignancies including pancreatic cancer. Recent studies show that *BRCA*-deficient tumors are likely to have higher infiltration of effector T lymphocyte [26,27].

The PD-1 receptor-ligand interaction is a major pathway hijacked by tumors to suppress immune control. The normal function of PD-1, expressed on the cell surface of activated T-cells under healthy conditions, is to down-modulate unwanted or excessive immune responses, including autoimmune reactions. PD-1 (encoded by the gene Pdcd1) is an immunoglobulin (Ig) superfamily member related to cluster of differentiation 28 (CD28) and cytotoxic T-lymphocyte-associated protein 4 (CTLA-4) that has been shown to negatively regulate antigen receptor signaling upon engagement of its ligands (PD-L1 and/or PD-L2) [28,29].

The structure of murine PD-1 has been resolved [30]. PD-1 and its family members are type I transmembrane glycoproteins containing an Ig-variable–type (IgV-type) domain responsible for ligand binding and a cytoplasmic tail responsible for the binding of signaling molecules. The cytoplasmic tail of PD-1 contains 2 tyrosine-based signaling motifs, an immunoreceptor tyrosine-based inhibition motif, and an immunoreceptor tyrosine-based switch motif. Following T-cell stimulation, PD-1 recruits the tyrosine phosphatases, SHP-1 and SHP-2, to the immunoreceptor tyrosine-based switch motif within its cytoplasmic tail, leading to the dephosphorylation of effector molecules such as CD3 zeta (CD3ζ), protein kinase C-theta (PKCθ), and zeta-chain-associated protein kinase (ZAP70), which are involved in the CD3 T-cell signaling cascade [29,31,32]. The mechanism by which PD-1 down-modulates T-cell responses is similar to, but distinct from, that of CTLA-4, because both molecules regulate an overlapping set of signaling proteins [33,34].

Despite disappointing results from recent clinical trials of single-agent or combination of PD-1 inhibition and CTLA-4 inhibition in PDAC, a number of preclinical literatures suggest that tumors with *BRCA*1/2 mutations are more immunogenic and the PD-1/PD-L1 pathway is an attractive target for therapeutic intervention in PDAC with *BRCA* mutations [1-3,26,27,35,36]. In a recent report of phase 2 study (TOPACIO) of PARPi (rucaparib) and pembrolizumab in advanced or metastatic triple negative breast cancer patients, "tBRCAmut" patients and "HRRmut" patients had higher objective response rate (ORR) at 47% and 40%, respectively, whereas PD-L1 positive patients had 32% of ORR [37]. Although it is a small phase 2 study and HRD signature was not available for many patients, this result is encouraging and support that primarily immunoresistant cancer can benefit from this combination of PARPi and PD-1 inhibition. Expanding on the concept of synthetic lethality in genes related to HRD, testing this hypothesis in PDAC with not only in germline *BRCA*1/2 mutations but also other candidates of germline and somatic HR-gene alterations will be crucial to improve patient outcomes of PDAC with HRD [23].

3.3 Rationale for the Trial and Selected Population

Pancreatic cancer (pancreatic ductal adenocarcinoma, PDAC) is one of the deadliest cancers with 5-year survival rate of 9% and will become the second leading cause of cancer-related deaths in the U.S. by 2030 [38,39]. Most patients diagnosed with PDAC have already advanced stage at presentation and developing effective treatment for these patients is the key to improve survival. Unfortunately, response rate is limited even with the most effective treatment and patient have to continue on cytotoxic chemotherapy and the treatments are continued for

extended time at a cost of different toxicities [22]. Thus, patients with stable disease or responding metastatic PDAC may benefit from less toxic maintenance strategy [40].

Unfortunately, PDAC is not responsive to single-agent immune checkpoint blockade (ICB) such as nivolumab or pembrolizumab unlike most other solid cancers [1-3]. Interestingly, the pattern of superior outcome of chemotherapy arm observed in the first 4 months in comparison to single-agent ICB arm may suggest that induction chemotherapy or combination is necessary in resistant solid cancers like PDAC [1-3,41]. Also, one possible explanation is that previous ICB trials have only evaluated PDAC as one disease without considering the impact of genomic subgroups [15]. Mismatch repair deficiency (dMMR or MSI-H) has recently been identified as a pan-cancer biomarker for ICB because these cancers have many mutations which produce neoantigens and allow them to be immunologically unique and better recognized by the patients' own immune system [42]. Similarly, high tumor mutation burden (TMB) also confers more neoantigens, therefore a sensitivity to ICB [43]. In PDAC, our group has previously shown that long-term survivors have high-quality tumor neoantigens and their immune system have a long-term memory against these neoantigens in their blood, equipping them for effective host immune surveillance and clearance of their cancer [44].

Although aforementioned MSI-H subgroup is rare (1%) in PDAC, we recently identified another important subgroup (19% of PDAC, *n*=262) with homologous recombination deficiency (HRD) [23,24]. (Figure 2) From unpublished data (4/25/2020 from cBioportal) at MSK, the distribution of HRD remains at 17% and HRD/DDR at 25% among n=2,175 PDAC patients which is similar to external cohort reported by Pishvaian et al. in national Know Your Tumor (KYT) program [45,46]. Additionally, we have observed an interesting population with exceptional platinum sensitivity without HRD/DDR-gene mutations. The HRD/DDR subgroup ranges 20-25% and the response rate of 33-50% observed in modified FOLFIRINOX treated metastatic PDAC supports the observation that an additional 15-20% derive major benefit from platinum therapy in the absence of HRD/DDR gene mutations [22]. We hypothesize that there may be other novel DDR-genes or factors beyond known genomic determinants that are not identifiable by targeted-gene sequencing assays that account for this population.

Figure 2. Oncoprint for HRD from MSK (*n*=262)

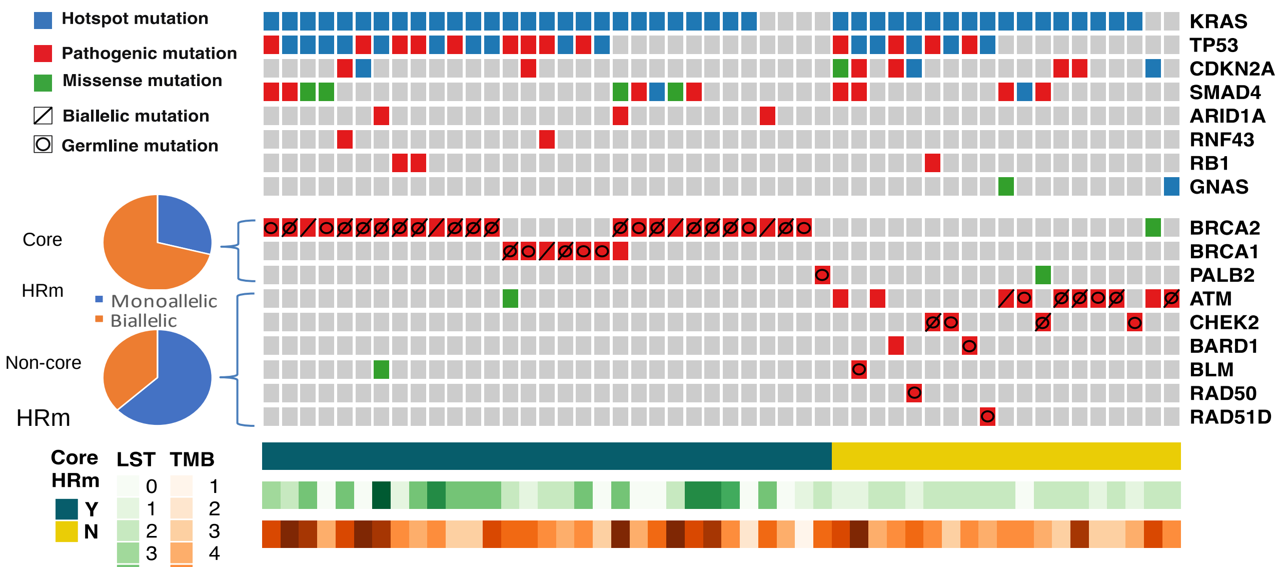

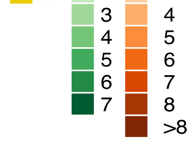

Oncoprint: pathogenic alterations of homologous recombination-genes and recurrent gene alterations from 50 patients with HRD are depicted (One patient had both germline and somatic BRCA variants). For each sample, mutation profile is shown in a column including 8 recurrently mutated genes (upper panel), 9 candidate HR-genes (lower panel). Pathogenic alterations included frameshift, truncating, splice site, or known pathogenic missense mutations, annotated by OncoKB. The labeling priority was in the order of hotspot alteration, pathogenic alteration, and missense variants of unknown significance (VUS). Core HRm (*BRCA1, BRCA2, and PALB2*) is shown as a track with green and yellow color bar. The level of genomic instability of each sample is depicted by LST score and TMB (mutation/MB) in the bars below. Biallelic HRm is annotated with a diagonal line whereas germline mutations are annotated with circles inside the square.

In our recent work (Figure 2, Park et al.), we demonstrated that these patients with HRD have higher TMB and large-scale state transition (LST) scores compared to patients without HRD indicating a higher level of genomic instability and have improved clinical outcomes [23]. Similar to higher immunogenicity and response to immunotherapy seen in cancer patients with other genomic instability conferred by dMMR or high TMB, tumors with HRD may respond better to novel combination immunotherapy with PARPi [42,47] Whether it is related to neoantigen quantity vs. quality, or other mechanisms need to be evaluated [48,49].

Synthetic lethality refers to an innovative mechanism by which sublethal DNA damage that can be repaired in a normal cell becomes lethal to a cancer that has a DNA damage repair defect, such as HRD [7,19,26]. This concept recently yielded a proof of principle result in the POLO trial, where germline *BRCA*-mutated PDAC patients were treated with olaparib, a poly ADP-ribose polymerase inhibitor (PARPi), for the maintenance after platinum-based first-line therapy and had a superior median progression-free survival (mPFS) compared to the placebo group (7.4 vs. 3.8 months; hazard ratio (HR), 0.53; 95% confidence interval [CI], 0.35 to 0.82; *p*=0.004), which led to an FDA approval [7]. Knowing that platinum-refractory PDAC patients despite somatic or germline BRCA mutations demonstrated resistance to PARPi in priorstudies, a maintenance strategy in platinum-sensitive PDAC is justified [19,50].

We hypothesize that the novel combination of pembrolizumab and olaparib, so-called POLAR, in HRD can be synergistic as preclinical models have shown that synthetic lethality can facilitate immunogenic cell death and can increase the number of CD8+ T cells and natural killer (NK) cells, as well as their production of IFN-γ and TNF-α, ultimately resulting in an improved response to ICB [51,52]. To test this hypothesis in three different HRD subgroups, this trial is designed as a phase II trial to enroll metastatic PDAC patients for genotype by HRD and for phenotype of platinum sensitivity. Currently, the same combination immunotherapy using pembrolizumab and olaparib is being tested in a large pan-cancer phase II trial in any advanced solid tumors; however, the focus of proposed POLAR trial is to study the translational science in addition to clinical activity dedicated for PDAC only (NCT04123366).

We will determine the immunologic role of synthetic lethality in metastatic PDAC with HRD and evaluate for improvement in clinical outcome with different degrees of synthetic lethality anticipated in different cohorts when treated with POLAR. The three cohorts are as following. Cohort A will include patients with either pathogenic germline or somatic alterations of 3 core homologous recombination-genes (HR-genes) - (*BRCA1/2, or PALB2*) and have stable or responding disease on first-line or second-line platinum therapy in two consecutive imaging assessments over at least 4 months or 16 weeks. Cohort B will include patients with non-core 15 HR-gene alterations (*ATM, BAP1, BARD1, BLM, BRIP1, CHEK2, FAM175A, FANCA, FANCC, MUTYH , NBN, RAD50, RAD51, RAD51C, RTEL1*) and have stable or responding disease on first-line or second-line platinum therapy in two consecutive imaging assessments over at least 4 months. Cohort C will include patients without any of the above HR-gene alterations included in Cohort A and B who have platinum-sensitivity, which is defined as a partial response (PR) or complete response (CR) for the best overall response (BOR) during at least 6 months on platinum-based therapy. Variants of unknown significance of candidate HR-genes from Cohort A or B will be eligible for Cohort C if they meet the partial response to platinum criterion. Of note, patients will be assigned and not randomized to Cohorts A, B and C.

All participating patients in the three cohorts will receive the combination of pembrolizumab 200 mg IV every 3 week and olaparib 300 mg twice day orally daily (POLAR) as maintenance therapy. After the first 6 months (9 cycles), on C10D1 Pembrolizumab 400 mg IV every 6 week plus olaparib 300 mg twice a day orally will be continued. Per treating investigator’s discretion, patients may remain at Pembrolizumab 200 mg IV every 3 weeks after the first 9 cycles. Additionally, to accomodate patients traveling far distances, if, per treating investigator, after the first or second imaging, there are no toxicities, patient’s dosing of Pembrolizumab may change to 400 mg IV every 6 weeks. All cycles will be 21-days (3 weeks) in length. The correlation of the immunogenomic landscape and spatiotemporal heterogeneity with clinical response to POLAR will provide an opportunity to develop genomic biomarkers and gather insights into resistance mechanism to POLAR.

4.0 OVERVIEW OF STUDY DESIGN/INTERVENTION

4.1 Design

This is a phase 2 open-label, non-randomized 3-cohort study to evaluate the safety and clinical efficacy of a novel combination - pembrolizumab and olaparib (POLAR) in genomically enriched three cohorts of metastatic PDAC patients with homologous recombination deficiency (HRD) as below.

Patients will be assigned to cohorts. Cohorts will be defined as following by CLIA-approved NGS or MSK-IMPACT Part A or C:

- **Cohort A**: Patients with either pathogenic germline or somatic alterations of 3 core homologous recombination-genes (HR-genes) - (*BRCA1/2, or PALB2*) who have stable or responding disease on first-line or second-line platinum therapy in two consecutive imaging assessments over at least 4 months or 16 weeks are eligible for inclusion in Cohort A.

- **Cohort B**: Patients with either pathogenic somatic or germline non-core 15 HR-gene alterations (*ATM, BAP1, BARD1, BLM, BRIP1, CHEK2, FAM175A, FANCA, FANCC, MUTYH, NBN, RAD50, RAD51, RAD51C, RTEL1*) who have stable or responding disease on first-line or second-line platinum therapy in two consecutive imaging assessments over at least 4 months are eligible for inclusion in Cohort B.

- **Cohort C**: Patients without any of the above HR-gene alterations included in Cohort A and B who have platinum-sensitivity, which is defined as a partial response (PR) or complete response (CR) for the best overall response (BOR) during at least 6 months on platinum-based therapy. Variants of unknown significance of candidate HR-genes from Cohort A or B will be eligible for **Cohort C** if they meet the partial response to platinum criterion.

All the participating patients will receive the combination of pembrolizumab 200 mg IV every 3 week and olaparib 300 mg twice day orally daily continuously (POLAR) as a maintenance therapy. After the first 6 months (9 cycles), on C10D1 Pembrolizumab 400 mg IV every 6 week plus olaparib 300 mg twice a day orally will be continued. Per treating investigator's discretion, patients may remain at Pembrolizumab 200 mg IV every 3 weeks after the first 9 cycles. Additionally, to accomodate patients traveling far distances, if, per treating investigator, after the first or second imaging, there are no toxicities, patient's dosing of Pembrolizumab may change to 400 mg IV every 6 weeks. All cycles will be 21-days (3 weeks) in length.

All the participants will be enrolled on #12-245 and #06-107 outside of this trial which will allow identification of which Cohort the patient will be assigned to and to permit future biospecimen use for research. Patients have a germline or somatic HR-gene alterations identified in an external CLIA or FDA-approved NGS panel including but not limited to, FoundationOne, Myriad, or Tempus, will be eligible. After enrollment, we will retrospectively confirm their HR-gene alteration status from both germline and somatic MSK-IMPACT profiling.

Study Endpoints:

- Primary endpoint: co-primary endpoints using either 6-month PFS rate by RECIST v1.1 and ORR by iRECIST as Figure 1. Schema.

- Secondary endpoints: disease control rate (DCR) by both RECIST v1.1 and iRECIST, best overall response (BOR), duration of response (DOR), PFS by both RECIST v1.1 and iRECIST, and OS. Biochemical markers (CEA, and CA 19-9 response). The safety of pembrolizumab and olaparib, toxicity grading by grading adverse events (AE) using CTCAE v. 5.0. To note, progression-free survival (PFS) will be analyzed from both the consent date and treatment start date (C1D1) (Reference study POLO defined PFS from the time of randomization not from C1D1, Golan 2019 NEJM).

- Exploratory endpoints: circulating tumor DNA by MSK-ACCESS, second progression-free survival (PFS2) from time of subsequent systemic treatment start post-POLAR to progression on that treatment, and duration of the first-line or second-line treatment prior to POLAR treatment. To also assess genetic signatures and compare clinical outcomes, evaluate reversion mutation, zygosity, plasticity and aneuploidy from tumor and ctDNA, and to assess the role of maintenance treatment with minimally residual disease by the presence of ctDNA and CA 19-9 and its trend prior to treatment.

- Correlative endpoints: *Detailed objectives are in Section 4.3 Biomarker Assessments*

- Explore HRD genetic signatures.
- Neoantigen surrogates (different mutation types, MHC I and II types, microbiome signatures). Peripheral blood and tumor immunoprofiling, T-cell receptor sequencing, transcriptomic, and proteomic analysis. Differential expression of immunologic gene and tissue protein.
- Intra/intertumoral heterogeneity and correlation of ctDNA to study early detection of resistance emergence.
- Prospective real-time organoids correlation will be recommended for all the participants if feasible.

Figure 3. Treatment and biospecimen Schema

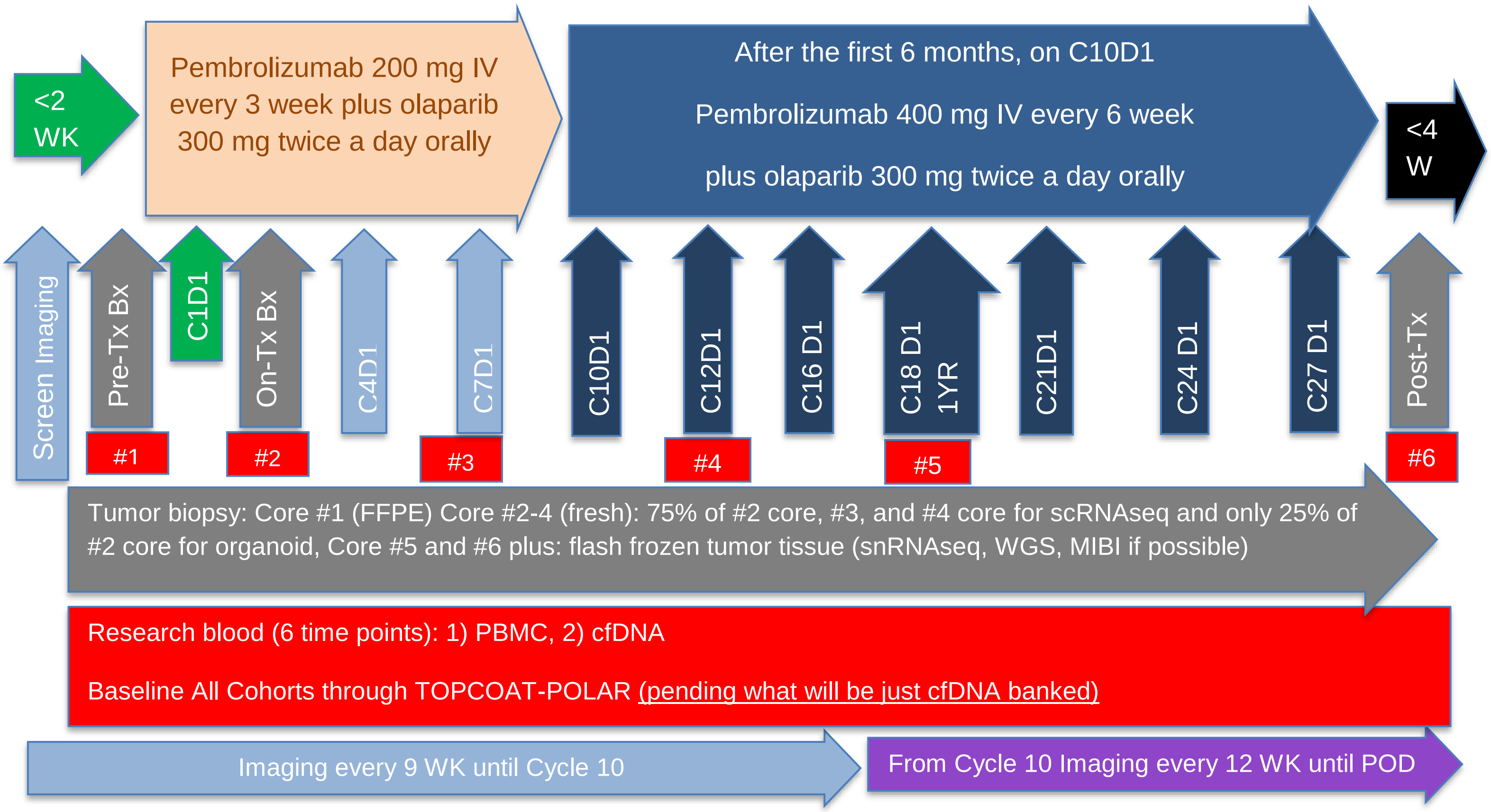

Tumor Response Assessments

Cross-sectional imaging will be obtained at baseline (within 28 days of treatment commencement) and following initiation of maintenance treatment every 9 weeks (+/- 7 days) until Cycle 10; thereafter imaging will be performed every 12 weeks. If a participant has remained on study for greater than 2 years, imaging can be transitioned to every 3-6 months per MD discretion. Permitted imaging includes: CT chest/abdomen/pelvis with IV and oral contrast or MRI abdomen/pelvis and non-contrast CT chest if patient cannot receive CT IV contrast.

4.2 Intervention

A variety of factors that could potentially predict clinical response to the POLAR regimen will be investigated in peripheral blood and in tumor specimens taken from all participants pre-treatment (screening phase), on-treatment, and at the time of disease progression, as outlined in the Treatment and Biospecimen Schema in Figure 1. Data from these investigations will be evaluated for associations with treatment response to POLAR, survival (OS, PFS), and safety (AE).

Research Tissue Collection

All participants will undergo a pre-treatment research biopsy and cfDNA at baseline and during treatment. (Figure 1.) All patients will receive the combination of pembrolizumab 200 mg IV

every 3 week and olaparib 300 mg twice day orally daily (POLAR) as maintenance therapy. After the first 6 months (9 cycles), on C10D1 Pembrolizumab 400 mg IV every 6 week plus olaparib 300 mg twice a day orally will be continued. At the discretion of the treating investigator, Pembrolizumab 400 mg IV every 6 weeks may begin earlier or Pembrolizumab 200 mg IV every 3 weeks may be continued after C10D1. An on-treatment biopsy (on the 6th- 9th week) and post-progression (before initiation of further therapy) will be obtained where feasible. Biopsies are mandated by protocol baseline and on treatment and at the time of progression except in the setting that there is no disease site that can be safely assessed for biopsy or other clinically relevant safety reason. If tissue acquisition is limited due to availability, available tissues will be prioritized in the following order.

1) FFPE (WES, multiplex immunohistochemistry)
2) Fresh tissue for single cell analysis with 10-20% cut for organoid culture
3) Fresh tissue for single cell analysis
4) Fresh frozen for archival

Patients will be presented at multidisciplinary tumor board for discussion related to biopsy safety and site choice were needed. Selected patients with disconcordant and heterogenous response can include up to three different sites for tissue sampling as long as deemed safe.

Research Blood Collection
Peripheral blood mononuclear cells, buffy coat, and plasma will be collected. Circulating tumor DNA on these patients identified as responders and resistant patients will be compared.
Please refer to Section 9 PRE-TREATMENT/INTERVENTION PLAN and 10 TREATMENT/INTERVENTION PLAN.

Samples collected at MSK will be processed and subsequently stored in liquid nitrogen at the Rubenstein M. David Center for Pancreatic Cancer Research at MSK, located on the 6th floor of the MSKCC Mortimer B. Zuckerman Research Center, 417 East 68th Street, New York, NY.

All samples will be labeled with the unique subject study identification number provided at registration, sample time point, and the date of the specimen.

Please see Appendix 3. Biomarker Management Plan (BMP) for further details on correlative sample collection details, timepoints, and processing locations.

4.3 Biomarker Assessments

The goal of correlative research: To describe the immunogenomic changes in PDAC when treated with POLAR. Patients' tumor biopsies and peripheral blood from baseline, on-treatment, and at disease progression, will be collected. Novel HRD signature and platinum-sensitivity signatures will be developed and additional immunotherapy targets will be evaluated using single-cell RNAseq technique and single-cell TCRseq, protein expression in bulk as well as single-cell analysis in select cases. Biopsies will be multiple core biopsies (where feasible) and normal adjacent tissue for comparison and biopsies from different lesions at progression will be

highly encouraged in appropriate clinical setting to generate organoids and intertumoral heterogeneity will be described.

Exploratory Objective 1: To describe different genomic and transcriptomic signatures among different HRD and platinum sensitivity cohorts which associates with sensitivity and resistant to POLAR regimen. Several HRD genetic mutational signatures (zygosity, genomic loss of heterozygosity [gLOH], large-scale state transition [LST], telomeric allelic imbalance [TAI], and Signature 3) will be analyzed at baseline in each subgroup (Cohort A, B, and C) will be evaluated. Also, novel genetic signature development will be explored. Iacobuzio lab and Riaz lab have expertise in genome analysis and will undertake this plan collaboratively.

Exploratory Objective 2: To describe and compare immunologic significance of POLAR treatment in each cohort. The neoantigen induction and the correlation of immunogenic tumor microenvironment (TME) and putative immunomodulatory targets from each cohort will be described. Different neoantigen algorithmic methods, TME and HRD signatures will be described. Innate and adaptive immunity via Stimulator of Interferon Genes (STING, STING/TBK1/IRF3) pathway, immunogenic cell death (ICD), DNA damage as well as the functional assay of PAR, r-H2AX, and RAD51C will be described [49]. Different immune cell profiling and cytokine analyses from tumor and peripheral blood will be described. Their subtype proportions and T cell receptor clonality change will be explored before and after the treatment and among subgroups. Balachandran lab has the expertise in neoantigen and immunologic analysis and will undertake this plan collaboratively.

> **Immunogenic cell death (ICD), innate and adaptive immunity landscape:** DNA damage, STING pathway (cGAS, STING, p-TBK1, IRF3), damage-associated molecular pattern (DAMP), immunogenic cell death, CARL, ATP, HMGB1, profiling and functional assays of immune cells (NK cell, memory, effector T cells, dendritic cells, macrophages, myeloid-derived suppressor cells, regulatory T cells) will be evaluated from tumor infiltrates and associated with peripheral blood tumor. TCR Vb clonality at baseline, on-treatment, and on-progression will be described [53].
>
> **Tumor microenvironment (TME) evaluation**: Cytokines (IFN-r, TNF-a, TGF-b, IL-6, 10), transcriptomic signatures, multispectral immunohistochemistry (e.g. coreceptors including PD-L1, A2AR, CD40, CD73, CD11b, CD68, CD103, TIGIT, VISTA) and immunofluorescent staining for coreceptors and different tumor immune infiltrates, stroma, and normal adjacent tissue will be evaluated. In limited exceptional clinical cases, single cell RNA sequencing will be performed.

Exploratory Objective 3: To evaluate the role of clonal evolution in spatiotemporal heterogeneity influencing the clinical outcome of HRD in patients with metastatic PDAC when treated with POLAR, circulating tumor DNA (ctDNA) and multiple longitudinal tumor biopsies of selected patients will be described to for potential resistance mechanism. Pe'er lab has the expertise in single-cell analysis technique and will undertake this plan collaboratively.

Exploratory Objective 4: To bank a large biorepository of organoids for patients with HRD. In select patients with viable organoids available from IRB# 15-149 prior to enrollment to POLAR trial, we will concurrently evaluate the response of organoid to POLAR in real time as participants are treated in parallel. Iacobuzio lab at Rubenstein Center for Pancreatic Cancer Research has extensive expertise in this field and will undertake this plan. We will collect fresh tissue for organoid development if available for biomarker and resistance studies.

5.0 THERAPEUTIC/DIAGNOSTIC AGENTS & NON-THERAPEUTIC ASSESSMENTS

Both pembrolizumab and olaparib will be supplied for all patients in the trial.

5.1 PEMBROLIZUMAB:

Active Ingredients, pharmacological class, dose formulation, storage:

Pembrolizumab is a potent humanized immunoglobulin G4 (IgG4) monoclonal antibody (mAb) with high specificity of binding to the programmed cell death 1 (PD-1) receptor, thus inhibiting its interaction with programmed cell death ligand 1 (PD-L1) and programmed cell death ligand 2 (PD-L2).

Pembrolizumab Solution for Infusion 100 mg/vial is a liquid DP (manufactured using the fully formulated DS with L-histidine as a buffering agent, polysorbate 80 as a surfactant, and sucrose as a stabilizer/tonicity modifier), and Water for Injection as Solvent). The DP is stored under refrigerated conditions (2°C to 8°C). The liquid is intended for IV administration. The DP is diluted with 0.9% sodium chloride injection, USP (normal saline) or 5% dextrose injection, USP (5% dextrose) to 1-10 mg/mL before intravenous (IV) administration through an infusion filter. Liquid DP is compatible with the IV bag and infusion-line materials. Reconstituted vials should be used immediately to prepare the infusion solution in the IV bag, and the infusion solution should be administered immediately. Do not freeze the diluted solution. If the diluted pembrolizumab solution is not used immediately, it may be stored for no more than 24 hours at 2°C to 8°C. This 24-hour total hold from reconstitution may include up to 6 hours at room temperature (at or below 25°C). Any additional hold time must be at 2°C to 8°C. If refrigerated, the vials and/or intravenous bags must be allowed to come to room temperature before use. The recommended dose of pembrolizumab in adults is either 200 mg Q3W or 400 mg Q6W administered as intravenous infusion over 30 minutes. The recommended dose of pembrolizumab in pediatric patients is 2 mg/kg (up to a maximum of 200 mg), administered as an IV infusion over 30 minutes Q3W. See prescribing information for specific dosing approved in each country.

The planned dose of pembrolizumab for this study is 200 mg every 3 weeks (Q3W). After the first 6 months (9 cycles), on C10D1 Pembrolizumab 400 mg IV every 6 week will be continued. Based on the totality of data generated in the Keytruda development program, 200 mg Q3W is

the appropriate dose of pembrolizumab for adults across all indications and regardless of tumor type.

For pharmacological class, *please refer to Investigator's Brochure.*

5.2 OLAPARIB:

Olaparib (AZD2281, KU-0059436) is a potent inhibitor of poly ADP-ribose polymerase (PARP) developed as a monotherapy as well as for combination with chemotherapy, ionizing radiation and other anti-cancer agents including novel agents and immunotherapy. The approved tradename for olaparib is LYNPARZA. Since 2012, the tablet formulation was designed to deliver the therapeutic dose of olaparib in fewer dose units than the capsule.

Active Ingredients and pharmacological class:

Chemical Name: 4-[(3-{[4-(cyclopropylcarbonyl)piperazin-1-yl]carbonyl}-4-fluorophenyl)methyl]phthalazin-1(2H)-one.

Dose formulation and storage:

Olaparib is presented for po administration as a green, film-coated tablet containing 25 mg, 100 mg or 150 mg of drug substance. The 100 mg strength is also available as a yellow, film-coated tablet. Olaparib tablets are supplied in high-density polyethylene (HDPE) bottles containing desiccant.. Bottles are secured with a child-resistant closure; induction-sealed membranes provide tamper evidence.

The planned dose of olaparib for this study is at a dose of 300 mg orally twice daily (bid). Olaparib is available as a green film-coated tablet containing 150 mg or 100 mg. The planned dose of 300 mg bid will be made up of two x 150 mg tablets bid with 100 mg tablets used to manage dose reductions.

Olaparib will be supplied by Merck. Co. LTD. through their collaboration with AstraZeneca.

Refer to the Investigator's Brochure for Preclinical and Clinical data.

6.0 CRITERIA FOR PARTICIPANT ELIGIBILITY

6.1 Participant Inclusion Criteria

1. This is an on-platinum maintenance trial either in a first-line or second-line setting. Participants must have either stable disease or responding disease on current first-line or second-line platinum treatment for metastatic disease.

2. Male or female patients with cytologically or histologically confirmed metastatic pancreas adenocarcinoma or acinar cell carcinoma with homologous recombination gene alterations or platinum sensitivity as per following cohort inclusion criteria below.

Patients will be assigned to cohorts based on their genetic alterations and clinical response. Patient stratification to different Cohorts will be in the order of more canonical homologous recombination-gene (HR-gene) order. For example, patients who meet criteria for both A and B Cohorts, they will be assigned to Cohort A, not B. Cohorts will be defined as following by CLIA-approved NGS or MSK-IMPACT Part A or C:

- **Cohort A**: Patients with either pathogenic germline or somatic alterations of 3 core HR-genes - (*BRCA1/2, or PALB2*) who have stable or responding disease on first-line or second-line platinum therapy in two consecutive imaging assessments over at least 4 months or 16 weeks are eligible for inclusion in Cohort A.

- **Cohort B**: Patients with either pathogenic somatic or germline non-core 15 HR-gene alterations (*ATM, BAP1, BARD1, BLM, BRIP1, CHEK2, FAM175A, FANCA, FANCC, MUTYH, NBN, RAD50, RAD51, RAD51C, RTEL1*) who have stable or responding disease on first-line or second-line platinum therapy in two consecutive imaging assessments over at least 4 months are eligible for inclusion in Cohort B.

- **Cohort C**: Patients without any of the above HR-gene alterations included in Cohort A and B who have platinum-sensitivity, which is defined as a partial response (PR) or complete response (CR) for the best overall response (BOR) during at least 6 months on platinum-based therapy. Variants of unknown significance of candidate HR-genes from Cohort A or B will be eligible for **Cohort C** if they meet the partial response to platinum criterion.

- Variants of unknown significance (VUS) or benign polymorphisms of above 17 HR-genes from Cohort A and B are considered non-pathogenic and will be excluded from cohort A or B. However, if the participant demonstrates platinum sensitivity, patient can be considered eligible for Cohort C.

3. A recurrence after curative surgery is eligible if the recurrence is > 6 months after the last date of adjuvant therapy and the participant has at least stable or responding disease on platinum therapy and meet the above genomic or platinum sensitivity criteria.

4. Eastern Cooperative Oncology Group (ECOG) performance status 0-2 (Appendix 1: ECOG)

5. Patients must have normal organ and bone marrow function measured within 28 days prior to administration of study treatment as defined as below:
   - Absolute neutrophil count (ANC) ≥1500/µL
   - Hemoglobin ≥9.0 g/dL or ≥5.6 mmol/La
   - Platelets ≥100 000/µL

- Total bilirubin ≤ 2 × ULN
- AST (SGOT) and ALT (SGPT) ≤2.5 × ULN (≤5 × ULN for if liver metastases)
- Creatinine ≤2 × ULN

6. Patients with measurable disease and/or non-measurable or no evidence of disease assessed at baseline by CT (or MRI where CT is contraindicated) are eligible.

7. Postmenopausal or evidence of non-childbearing status for women of childbearing potential: negative urine or serum pregnancy test. Postmenopausal is defined as:

- Amenorrhoeic for 1 year or more following cessation of exogenous hormonal treatments
- Luteinizing hormone (LH) and Follicle stimulating hormone (FSH) levels in the post-menopausal range for women under 50
- Radiation-induced oophorectomy with last menses >1 year ago
- Chemotherapy-induced menopause with >1-year interval since last menses
- Surgical sterilization (bilateral oophorectomy or hysterectomy)

6.2 Participant Exclusion Criteria

1. Disease progression on either a first-line or the second-line platinum for metastatic PDAC or acinar cell carcinoma.

2. Patients with a second (or more) primary cancer, EXCEPTIONS: adequately treated nonmelanoma skin cancer, curatively treated in-situ cancer of the cervix, Ductal Carcinoma in Situ (DCIS), stage 1 cancers or low grade lymphomas curatively treated, without evidence of disease, and not requiring any active treatment prior to study entry, are eligible.

3. Resting EKG with QTC ≥ 450 msec detected on 2 or more time points within a 24-hour period or family history of long QT syndrome. If EKG demonstrates QTC ≥ 450 msec, patient will only be eligible if repeat EKG demonstrates QTC ≤ 450 msec. Fridericia QTc is acceptable for eligibility.

4. Patients with myelodysplastic syndrome/acute myeloid leukaemia or with features suggestive of MDS/AML.

5. Previous allogeneic bone marrow transplant.

6. Patients with symptomatic uncontrolled brain metastases. A scan to confirm the absence of brain metastases is not required for eligibility. The patient can receive a stable dose of corticosteroids before and during the study as long as the steroids were started at least 4 weeks prior to treatment and the steroid dose is ≤ 10 mg/day. Patients with spinal cord

compression unless considered to have received definitive treatment for this and evidence of clinically stable disease for 28 days.

7. Patients unable to swallow orally administered medication and patients with gastrointestinal disorders likely to interfere with absorption of the study medication.

8. A diagnosis of immunodeficiency or is receiving chronic systemic steroid therapy (in dosing exceeding 10 mg daily of prednisone equivalent) or any other form of immunosuppressive therapy within 7 days prior to the first dose of study drug.

9. Immunocompromised patients, e.g., patients who are known to be serologically positive for human immunodeficiency virus (HIV).

10. Patients with known active hepatitis (i.e. Hepatitis B or C).
    a. Active hepatitis B virus (HBV) is defined by a known positive HBV surface antigen (HBsAg) result. Patients with a past or resolved HBV infection (defined as the presence of hepatitis B core antibody and absence of HBsAg) are eligible.

    b. Patients positive for hepatitis C virus (HCV) antibody are eligible only if polymerase chain reaction is negative for HCV RNA.

    c. Has a known history of active TB (Bacillus tuberculosis).

11. Any prior treatment with any PARP inhibitor, including olaparib.

12. Any previous treatment with any anti-PD-1, anti-PD-L1, or anti-PD-L2 agent.

13. Has received systemic therapy 2 weeks prior to starting treatment

14. Patients with any recent investigational agents are not eligible unless at least 2 weeks or 5 half-lives of investigational agent prior to the first dose of study treatment have passed.

15. Live vaccines within 30 days prior to the first dose of study treatment and while participating in the study are not permitted. Examples of live vaccines include, but are not limited to, the following: measles, mumps, rubella, varicella/zoster, yellow fever, rabies, BCG, and typhoid vaccine. Seasonal influenza vaccines for injection are generally killed virus vaccines and are allowed; however, intranasal influenza vaccines (e.g., FluMist®) are live attenuated vaccines and are not allowed.

16. Active autoimmune disease that has required systemic treatment in the past 2 years is not permitted (i.e. with use of disease modifying agents, corticosteroids or immunosuppressive drugs). Replacement therapy (e.g., thyroxine, insulin, or physiologic corticosteroid replacement therapy for adrenal or pituitary insufficiency, etc.) is not considered a form of systemic treatment.

17. Palliative radiotherapy must have been completed 14 or more days before Cycle 1 Day 1.

18. If participant received major surgery within 4 weeks before screening, they must have recovered adequately from the toxicity and/or complications from the intervention prior to starting study treatment.

19. Participants must have recovered from all AEs due to previous therapies to ≤Grade 1 or baseline. Participants with ≤Grade 2 neuropathy may be eligible. Participants with ≤Grade 2 alopecia may be eligible as well.

20. Concomitant use of known strong CYP3A inhibitors (e.g. itraconazole, telithromycin, clarithromycin, protease inhibitors boosted with ritonavir or cobicistat, indinavir, saquinavir, nelfinavir, boceprevir, telaprevir) or moderate CYP3A inhibitors (e.g. ciprofloxacin, erythromycin, diltiazem, fluconazole, verapamil). The required washout period prior to starting is 2 weeks.

21. Concomitant use of known strong (e.g. phenobarbital, enzalutamide, phenytoin, rifampicin, rifabutin, rifapentine, carbamazepine, nevirapine and St John's Wort) or moderate CYP3A inducers (e.g. bosentan, efavirenz, modafinil). The required washout period prior to starting is 5 weeks for enzalutamide or phenobarbital and 3 weeks for other agents.

7.0 RECRUITMENT PLAN

All participants meeting the eligibility requirements will be considered for enrollment regardless of sex, race, or religion. Subjects will be accrued from the Solid Tumor Gastrointestinal Oncology Service (STGI). Eligibility criteria may not be waived by the investigator. Discussions regarding protocol enrollment and participant eligibility will begin with any of the investigators named on the consenting professionals list. Participants will be made aware of the protocol, its specific aims and objectives, and the potential risks and benefits the subjects may incur. Participants will be required to read, agree to, and sign an institutional review board (IRB)-approved informed consent form prior to registration for this trial. Participants will be consented prior to screening activities and treatment initiation. Our target accrual is minimum 47 (Cohort A: 18, B: 15, C: 15) to maximum 63 eligible patients. Approximately 800 patients with PDAC are seen at MSK annually. About 125 patients have HRD who are expected to meet the genetic eligibility criteria for Cohort A and B and about 50-75 for Cohort C each year. Based on our experience with previous trials of this type, we expect an accrual time of approximately 2.5-3 years.

7.1 Research Participant Registration

Confirm eligibility as defined in the section entitled Inclusion/Exclusion Criteria. Obtain informed consent, by following procedures defined in section entitled Informed Consent Procedures. During the registration process registering patients will be required to complete a protocol specific Eligibility Checklist. The patients signing the Eligibility Checklist is confirming whether the participant is eligible to enroll in the study. Study staff are responsible for ensuring that all institutional requirements necessary to enroll a participant to the study have been

completed. See related Clinical Research Policy and Procedure #401 (Protocol Participant Registration).

7.2 Randomization

This study is an open-label, non-randomized, genomic biomarker-enriched study. Patients are allocated to the 3 cohorts based on genomic profile and platinum sensitivity.

8.0 INFORMED CONSENT PROCEDURES

Before protocol-specified procedures are carried out, consenting professionals will explain full details of the protocol and study procedures as well as the risks involved to participants prior to their inclusion in the study. Participants will also be informed that they are free to withdraw from the study at any time. All participants must sign an IRB/PB-approved consent form indicating their consent to participate. This consent form meets the requirements of the Code of Federal Regulations and the Institutional Review Board/Privacy Board of this Center. The consent form will include the following:

1. The nature and objectives, potential risks and benefits of the intended study.
2. The length of study and the likely follow-up required.
3. Alternatives to the proposed study. (This will include available standard and investigational therapies. In addition, patients will be offered an option of supportive care for therapeutic studies.)
4. The name of the investigator(s) responsible for the protocol.
5. The right of the participant to accept or refuse study interventions/interactions and to withdraw from participation at any time.
6. The voluntary consent for #12-245 and #06-107 outside of this trial which will allow identification of which group the patient was treated under and future biospecimen use for research. The consent will also allow adjacent normal tissue near cancerous tissue to be collected for research analysis.

Before any protocol-specific procedures can be carried out, the consenting professional will fully explain the aspects of patient privacy concerning research specific information. In addition to signing the IRB Informed Consent, all patients must agree to the Research Authorization component of the informed consent form.

Each participant and consenting professional will sign the consent form. The participant must receive a copy of the signed informed consent form.

9.0 PRE-TREATMENT/INTERVENTION

- Prior to treatment initiation, all subjects will undergo the following procedures: Separate cross-sectional imaging: CT chest/abdomen/pelvis with contrast or MRI abdomen/pelvis and chest CT without contrast (if IV contrast is contraindicated) within 4 weeks of planned first

date of POLAR treatment. Separate CT scans of the chest, abdomen, and pelvis are acceptable, provided they are done 4 weeks of the first treatment.

- **Research Blood Draws**

All collected blood samples at MSK will be processed by the following protocol:

In addition, the following evaluations will be required at the times indicated in Section 11.1 Adverse Event (AE) Monitoring Table 10.

Table 1. Standard-of-care and research blood draws

Standard-of-care labs	• Hematology: complete blood count (CBC), including red blood cell (RBC) count, Hgb, hematocrit, platelet count, and WBC count with differential (neutrophils, eosinophils, lymphocytes, monocytes, basophils, and other cells)<br>• Chemistry panel (serum or plasma): sodium, potassium, chloride, bicarbonate, blood urea nitrogen (BUN) or urea, creatinine, glucose, calcium, magnesium, phosphorus, total bilirubin, ALT, AST, alkaline phosphatase, lactate dehydrogenase (LDH), total protein, albumin, amylase, and lipase<br>• Coagulation: prothrombin time (PT), activated partial thromboplastin time (aPTT), and international normalized ratio (INR)<br>• Pregnancy test*:* All women of childbearing potential *(*including those who have had a tubal ligation). If a urine pregnancy test result is positive, dosing will be delayed until the patient's status is determined by a serum pregnancy test and it is confirmed that the result is not a false positive due to HCG tumor secretion. The pregnancy test should be during screening within 28 days prior to the start of study treatment.<br>• Urinalysis: dipstick (pH, specific gravity, glucose, protein, ketones, blood) and microscopic examination (sediment, RBCs, WBCs, casts, crystals, epithelial cells, bacteria) if warranted by dipstick results<br>• Thyroid function testing: thyroid-stimulating hormone (TSH), free T3, and free T4<br>• C-reactive protein (CRP)<br><br>• Serology: (Only if these were not performed at MSK)<br>○ Hepatitis B surface antigen (HBsAg), antibodies against HBsAg, and hepatitis B core antigen. Hepatitis B virus (HBV) DNA test is to be obtained prior to Cycle 1, Day 1 if the patient has positive serology for total hepatitis B core antibody (anti-HBc)<br>○ Anti-hepatitis C virus antibody (HCV). HCV RNA test is required prior to Cycle 1, Day 1 for consideration of eligibility if the patient has positive serology for anti-HCV<br>○ HIV antibodies
Research blood	• Blood is stored at 20-25°C up to 4 hours before processing<br>• Phosphate-buffered saline (PBS) is added to blood samples to achieve a total volume of 50 mL

	• 15 mL of Ficoll-Paque PLUS is added to the blood samples<br>• Samples are centrifuged at 1000g for 20 minutes at 21ºC<br>• The layer containing PBMCs is harvested<br>• BEAD solution containing 500 mL of PBS and 5 mL of fetal bovine serum is added to the PBMCs to achieve a final volume of 50 mL<br>• Samples are centrifuged at 650g for 5 minutes at 4ºC, and the supernatant is discarded<br>• 1-2 mL of BEAD is added to the remaining PBMCs, and samples are centrifuged at 250g for 10 minutes at 4ºC<br>• The supernatant is discarded, and cells are resuspended in freezing media containing 90% fetal bovine serum and 10% dimethyl sulfoxide<br>○ Cells are frozen in a -80ºC freezer for 24 hours and then transferred to liquid nitrogen for long-term storage<br>○ Epstein-Barr nuclear antigen (EBNA) IgG<br>Cytomegalovirus (CMV) IgG

10.0 TREATMENT/INTERVENTION PLAN

10.1 Administration

Within 4 weeks of screening date, eligible patients will receive combination pembrolizumab and olaparib as maintenance therapy until clinical or radiographic progression or discontinuation due to -limiting toxicity. If a patient requires discontinuation of either olaparib or pembrolizumab for reasons of toxicity and if in the opinion of the treating MD the patient is continuing to derive benefit, the patient is able to continue on study. Additionally, in the absence of unacceptable toxicity, participants who meet criteria for disease progression per RECIST 1.1 while receiving treatment will be permitted to continue treatment, if in the opinion of the treating MD the patient is continuing to derive benefit from the treatment. iRECIST will be used to evaluate the objective response rate and the co-primary endpoint of overall response rate will be assessed by RECIST v1.1. (Section 12.0 CRITERIA FOR REMOVAL FROM STUDY).

The POLAR treatment will be administered to subjects as outlined in Figure 1 - Schematic treatment and biospecimen collection schedule. Approximately within 4 weeks of screen visit, patient will get baseline biopsy and will start the first dose pembrolizumab 200 mg IV every 3 weeks and olaparib 300 mg twice daily orally. After the first 6 months (9 cycles), on C10D1 Pembrolizumab 400 mg IV every 6 week plus olaparib 300 mg twice a day orally will be continued. Per treating investigator's discretion, patients may remain at Pembrolizumab 200 mg IV every 3 weeks after the first 9 cycles. Additionally, to accomodate patients traveling far distances, if, per treating investigator, after the first or second imaging, there are no toxicities, patient's dosing of Pembrolizumab may change to 400 mg IV every 6 weeks. All cycles will be 21 days (3 weeks) in length. Subjects will be monitored for toxicity every three weeks for the first 9 cycles, and every six weeks thereafter, unless clinically indicated to be monitored every 3 weeks.

Table 2. Trial treatment (POLAR)

Drug	Dose	Dose Frequency	Route of Administration	Treatment Period	Use
Pembrolizumab	200 mg	Q3W	IV infusion	Day 1 of each 3-week cycle for cycle 1-9	experimental
Pembrolizumab	400mg	Q6W	IV infusion	Day 1 of every even cycle beginning with Cycle 10	experimental
Olaparib	300 mg (two 150 mg)	BID	Orally	Daily continuously	experimental

The following parameters must be acceptable prior to initiation of protocol treatment:

- Absolute neutrophil count (ANC) ≥1500/μL
- Hemoglobin ≥9.0 g/dL or ≥5.6 mmol/La
- Platelets ≥100 000/μL
- Total bilirubin ≤ 2 × ULN
- AST (SGOT) and ALT (SGPT) ≤2.5 × ULN (≤5 × ULN for if liver metastases)
- Creatinine ≤2 × ULN

10.2 Timing of Dose Administration

Trial treatment should be administered on Day 1 of each cycle after all procedures or assessments have been completed as detailed on the Trial Flow Chart (Section 6.0). Trial treatment may be administered up to 3 days before or after the scheduled Day 1 of each cycle due to administrative reasons. On the day of the treatment, participants can receive oral olaparib AM dose at the clinic visit followed by pembrolizumab.

All trial treatments will be administered on an outpatient basis.

Pembrolizumab will be infused per MSK guidelines. Pembrolizumab 200 mg will be administered as a 30-minute IV infusion every 3 week. After the first 6 months (9 cycles), on C10D1 Pembrolizumab 400 mg IV every 6 week plus olaparib 300 mg twice a day orally will be continued. Pembrolizumab 200 mg IV every 3 weeks may continue after Cycle 9, and Pembrolizumab 400 mg IV every 6 weeks may begin earlier than Cycle 9 per treating physician's discretion. Given the variability of infusion pumps a window of -5 minutes and +10 minutes is permitted (i.e., infusion time is 30 minutes: -5 min/+10 min).

10.3 Dose Modification and Toxicity Management for Immune-Related AEs Associated with Pembrolizumab

AEs associated with pembrolizumab exposure may represent an immunologic etiology. These immune-related AEs (irAEs) may occur shortly after the first dose or several months after the last dose of pembrolizumab treatment and may affect more than on body system simultaneously. Therefore, early recognition and initiation of treatment is critical to reduce complications. Based on existing clinical study data, most irAEs were reversible and could be managed with interruptions of pembrolizumab, administration of corticosteroids and/or other supportive care. For suspected irAEs, ensure adequate evaluation to confirm etiology or exclude other causes. Additional procedures or tests such as bronchoscopy, endoscopy, skin biopsy may be included as part of the evaluation. Based on the severity of irAEs, withhold or permanently discontinue pembrolizumab and administer corticosteroids. Dose modification and toxicity management guidelines for irAEs associated with pembrolizumab are provided in Table 3.

Table 3 Dose modification and toxicity management guidelines for immune-related AEs associated with pembrolizumab

General instructions:

1. Severe and life-threatening irAEs should be treated with IV corticosteroids followed by oral steroids. Other immunosuppressive treatment should begin if the irAEs are not controlled by corticosteroids.
2. Pembrolizumab must be permanently discontinued if the irAE does not resolve or the corticosteroid dose is not ≤10 mg/day within 12 weeks of the last pembrolizumab treatment.
3. The corticosteroid taper should begin when the irAE is ≤ Grade 1 and continue at least 4 weeks.
4. If pembrolizumab has been withheld, pembrolizumab may resume after the irAE decreased to ≤ Grade 1 after corticosteroid taper.

irAEs	Toxicity grade (CTCAE V5.0)	Action with pembrolizumab	Corticosteroid and/or other therapies	Monitoring and follow-up
Pneumonitis	Grade 2	Withhold	• Administer corticosteroids (initial dose of 1 - 2 mg/kg prednisone or equivalent) followed by taper<br>• Add prophylactic antibiotics for opportunistic infections	• Monitor participants for signs and symptoms of pneumonitis<br>• Evaluate participants with suspected pneumonitis with radiographic imaging and initiate corticosteroid treatment
	Grade 3 or 4, or recurrent Grade 2	Permanently discontinue		
Diarrhea / Colitis	Grade 2 or 3	Withhold	• Administer corticosteroids (initial dose of 1 - 2 mg/kg prednisone or equivalent) followed by taper	• Monitor participants for signs and symptoms of enterocolitis (i.e., diarrhea, abdominal pain, blood or mucus in stool with or without fever) and of bowel perforation (i.e., peritoneal signs and ileus)<br>• Participants with ≥Grade 2 diarrhea suspecting colitis should consider GI consultation and performing endoscopy to rule out colitis
	Grade 4 or recurrent Grade 3	Permanently discontinue		

				• Participants with diarrhea/colitis should be advised to drink liberal quantities of clear fluids. If sufficient oral fluid intake is not feasible, fluid and electrolytes should be substituted via IV infusion
AST or ALT elevation or Increased Bilirubin	Grade 2 [a]	Withhold	• Administer corticosteroids (initial dose of 0.5 - 1 mg/kg prednisone or equivalent) followed by taper	• Monitor with liver function tests (consider weekly or more frequently until liver enzyme value returned to baseline or is stable)
	Grade 3 [b] or 4 [c]	Permanently discontinue	• Administer corticosteroids (initial dose of 1 - 2 mg/kg prednisone or equivalent) followed by taper	
Type 1 diabetes mellitus (T1DM) or Hyperglycemia	New onset T1DM or Grade 3 or 4 hyperglycemia associated with evidence of β-cell failure	Withhold [d]	• Initiate insulin replacement therapy for participants with T1DM<br>• Administer anti-hyperglycemic in participants with hyperglycemia	• Monitor participants for hyperglycemia or other signs and symptoms of diabetes
Hypophysitis	Grade 2	Withhold	• Administer corticosteroids and initiate hormonal replacements as clinically indicated	• Monitor for signs and symptoms of hypophysitis (including hypopituitarism and adrenal insufficiency)
	Grade 3 or 4	Withhold or permanently discontinue [d]		
Hyperthyroidism	Grade 2	Continue	• Treat with non-selective beta-blockers (e.g., propranolol) or thionamides as appropriate	• Monitor for signs and symptoms of thyroid disorders
	Grade 3 or 4	Withhold or permanently discontinue [d]		

Hypothyroidism	Grade 2, 3, or 4	Continue	• Initiate thyroid replacement hormones (e.g., levothyroxine or liothyronine) per standard of care	• Monitor for signs and symptoms of thyroid disorders
Nephritis and renal dysfunction: grading according to increased creatinine or acute kidney injury	Grade 2	Withhold	• Administer corticosteroids (prednisone 1 – 2 mg/kg or equivalent) followed by taper	• Monitor changes of renal function
	Grade 3 or 4	Permanently discontinue		
Myocarditis	Grade 1 or 2	Withhold	• Based on severity of AE administer corticosteroids	• Ensure adequate evaluation to confirm etiology and/or exclude other causes
	Grade 3 or 4	Permanently discontinue		
All Other immune-related AEs	Intolerable/persistent Grade 2	Withhold	• Based on severity of AE administer corticosteroids	• Ensure adequate evaluation to confirm etiology or exclude other causes
	Grade 3	Withhold or discontinue based on the event [e].		
	Grade 4 or recurrent Grade 3	Permanently discontinue		

[a] AST/ALT: >3.0 - 5.0 x ULN if baseline normal; >3.0 - 5.0 x baseline, if baseline abnormal; bilirubin:>1.5 - 3.0 x ULN if baseline normal; >1.5 - 3.0 x baseline if baseline abnormal

[b] AST/ALT: >5.0 to 20.0 x ULN, if baseline normal; >5.0 - 20.0 x baseline, if baseline abnormal; bilirubin:>3.0 - 10.0 x ULN if baseline normal; >3.0 - 10.0 x baseline if baseline abnormal

[c] AST/ALT: >20.0 x ULN, if baseline normal; >20.0 x baseline, if baseline abnormal; bilirubin: >10.0 x ULN if baseline normal; >10.0 x baseline if baseline abnormal

[d] The decision to withhold or permanently discontinue pembrolizumab is at the discretion of the investigator or treating physician. For participants with Grade 3 or 4 immune-related endocrinopathy where withhold of pembrolizumab is required, pembrolizumab may be resumed when AE resolves to ≤ Grade 2 and is controlled with hormonal replacement therapy or achieved metabolic control (in case of T1DM)

[e] Events that require discontinuation include but are not limited to: Guillain-Barre Syndrome, encephalitis, Stevens-Johnson Syndrome and toxic epidermal necrolysis.

Pembrolizumab may cause severe or life-threatening infusion-reactions including severe hypersensitivity or anaphylaxis. Signs and symptoms usually develop during or shortly after drug infusion and generally resolve completely within 24 hours of completion of infusion. Dose modification and toxicity management guidelines on pembrolizumab associated infusion reaction are provided in Table 4. If the patient has grade 3 LFT's not attibruted to drug, patient may continue treatment, per treating MD's discretion.

Table 4. Pembrolizumab infusion reaction dose modification and treatment guidelines

NCI CTCAE Grade	Treatment	Premedication at Subsequent Dosing
**Grade 1**<br>Mild reaction; infusion interruption not indicated; intervention not indicated	Increase monitoring of vital signs as medically indicated until the participant is deemed medically stable in the opinion of the investigator.	None
**Grade 2**<br>Requires therapy or infusion interruption but responds promptly to symptomatic treatment (e.g., antihistamines, NSAIDs, narcotics, IV fluids); prophylactic medications indicated for ≤24 hrs	**Stop Infusion.**<br>Additional appropriate medical therapy may include but is not limited to:<br>IV fluids<br>Antihistamines<br>NSAIDs<br>Acetaminophen<br>Narcotics<br>Increase monitoring of vital signs as medically indicated until the participant is deemed medically stable in the opinion of the investigator.<br>If symptoms resolve within 1 hour of stopping drug infusion, the infusion may be restarted at 50% of the original infusion rate (e.g. from 100 mL/hr to 50 mL/hr). Otherwise dosing will be held until symptoms resolve and the participant should be premedicated for the next scheduled dose.<br>**Participants who develop Grade 2 toxicity despite adequate premedication should be permanently discontinued from further study drug intervention**	Participant may be premedicated 1.5h (± 30 minutes) prior to infusion of _____ with:<br>Diphenhydramine 50 mg po (or equivalent dose of antihistamine).<br>Acetaminophen 500-1000 mg po (or equivalent dose of analgesic).
**Grades 3 or 4**<br>Grade 3:<br>Prolonged (i.e., not rapidly responsive to symptomatic medication and/or brief interruption of infusion); recurrence of symptoms following initial improvement; hospitalization indicated for other clinical sequelae (e.g., renal impairment, pulmonary infiltrates)<br>Grade 4:	**Stop Infusion.**<br>Additional appropriate medical therapy may include but is not limited to:<br>Epinephrine**<br>IV fluids<br>Antihistamines<br>NSAIDs<br>Acetaminophen<br>Narcotics<br>Oxygen<br>Pressors<br>Corticosteroids<br>Increase monitoring of vital signs as medically indicated until the participant is deemed medically stable in the opinion of the investigator.<br>Hospitalization may be indicated.<br>**In cases of anaphylaxis, epinephrine should be used immediately.	No subsequent dosing

Life-threatening; pressor or ventilatory support indicated	**Participant is permanently discontinued from further study drug intervention.**	
Appropriate resuscitation equipment should be available at the bedside and a physician readily available during the period of drug administration.<br>For further information, please refer to the Common Terminology Criteria for Adverse Events v5.0 (CTCAE) at http://ctep.cancer.gov		

Other allowed dose interruption for pembrolizumab

Pembrolizumab may be interrupted for situations other than treatment-related AEs such as medical/ surgical events or logistical reasons not related to study therapy. Participants should be placed back on study therapy within 3 weeks of the scheduled interruption, unless otherwise discussed with the Sponsor. The reason for interruption should be documented in the patient's study record.

10.4 Rescue Medications & Supportive Care for AEs Associated with Pembroizumab

Participants should receive appropriate supportive care measures as deemed necessary by the treating investigator. Suggested supportive care measures for the management of AEs with potential immunologic etiology are outlined along with the dose modification guidelines in Section 5.2.2, [Table 3]. Where appropriate, these guidelines include the use of oral or IV treatment with corticosteroids, as well as additional anti-inflammatory agents if symptoms do not improve with administration of corticosteroids. Note that several courses of steroid tapering may be necessary as symptoms may worsen when the steroid dose is decreased. For each disorder, attempts should be made to rule out other causes such as metastatic disease or bacterial or viral infection, which might require additional supportive care. The treatment guidelines are intended to be applied when the Investigator determines the events to be related to pembrolizumab.

It may be necessary to perform conditional procedures such as bronchoscopy, endoscopy, or skin photography as part of evaluation of the event.

10.7 Dose modification and toxicity management for AEs associated with olaparib

Dose Reductions

For guidance on dose reductions when concomitant strong or moderate CYP3A inhibitors cannot be avoided see below Table 6 and Table 7.

When dose reduction is necessary patients will take one 150 mg tablet and one 100 mg tablet twice daily or two x 100 mg tablet twice daily, or one 150 mg tablet twice daily or one 100 mg tablet twice daily. If further reduction is required, treating investigators should follow the SOC use for Olaparib.

In case a dose reduction is necessary, the Study treatment will be administered as follows:

Table 5. Dose reductions for study treatment to manage adverse events

Initial Dose	Following re-challenge post interruption: Dose reduction 1	Dose reduction 2*
300 mg twice daily<br>(Two 150 mg tablets)	250 mg twice daily<br>(150 mg tablet and 100 mg tablet)	200 mg twice daily<br>(Two 100 mg tablets)

*If further reduction is required, treating investigators should follow the SOC use for Olaparib.

Table 6. Dose reduction for study treatment if patient develops moderate renal impairment

Initial Dose	Moderate renal impairment (calculated creatinine clearance by Cockcroft -Gault equation or based on a 24-hour urine test between 31 and 50 ml/min): Dose reduction
300 mg twice daily	CrCl 51 to 80 mL/minute: No dosage adjustment necessary.<br>CrCl 31 to 50 mL/minute: Reduce dose to 200 mg twice daily.<br>CrCl ≤30 mL/minute: There are no dosage adjustments provided in the manufacturer's labeling (has not been studied).<br>End-stage renal disease: There are no dosage adjustments provided in the manufacturer's labeling (has not been studied).

10.6 Management of adverse events for <u>AEs associated with olaparib</u>

Any toxicity observed during the course of the study could be managed by interruption of the dose of study treatment or dose reductions. Repeat dose interruptions are allowed as required, for a maximum of 4 weeks on each occasion. If the interruption is any longer, the study team must be informed. Study treatment can be dose reduced to 250 mg twice daily as a first step and to 200 mg twice daily as a second step. If further reduction is required, treating invesigators should follow the SOC use for Olaparib. If patient is clinically benefiting from prior POLAR treatment, patient can continue to receive pembrolizumab alone or olaparib alone depending on the specific toxicity related to one of these agents. Once dose is reduced, escalation is not permitted (except following concomitant treatment with CYP3A4 inhibitors – see Section 6.5

10.6.1 Management of hematological toxicity associated with olaparib

10.6.1.1 Management of anemia associated with olaparib

Table 7. Management of anemia

**Hemoglobin**	**Action to be taken**
**Hb < 10** ***but*** **≥ 8 g/dl (CTCAE Grade 2)**	**First occurrence:**<br>Give appropriate supportive treatment and investigate causality.<br>Investigator judgement to continue olaparib or combination treatment with supportive treatment (e.g. transfusion) *or* interrupt dose for a maximum of 4 weeks. Study treatment can be restarted if Hb has recovered to > 8g/dl.<br><br>**Subsequent occurrences:**<br>If Hb**< 9** ***but*** **≥ 8 g/dl**, dose interrupt (for max of 4 weeks) olaparib or combination until Hb ≥ 9 g/dl and upon recovery dose reduction may be considered (to **250 mg twice daily** as a first step and to **200 mg twice daily** as a second step).
**Hb < 8 g/dl**<br>**(CTCAE Grade 3)**	Give appropriate supportive treatment (e.g. transfusion) and investigate causality.<br>Interrupt olaparib or combination treatment for a maximum of 4 weeks until improved to Hb ≥ 8 g/dl.<br>Upon recovery dose reduce to **250 mg twice daily** as a first step and to **200 mg twice daily** as a second step in the case of repeat Hb decrease.

**Please note, if Hb < 8 g/dl, Hb needs to recover to ≥ 8 g/dl to resume with a reduced dose.

Common treatable causes of anemia (e.g., iron, vitamin B12 or folate deficiencies and hypothyroidism) should be investigated and appropriately managed. In some cases, management of anemia may require blood transfusions. For cases where patients develop prolonged hematological toxicity (≥2-week interruption/delay in study treatment due to CTC grade 3 or worse anemia and/or development of blood transfusion dependence), refer to guidance later in this section for the management of this.

10.6.1.2 Management of neutropenia, leukopenia and thrombocytopenia associated with olaparib

Table 8. Management of neutropenia, leukopenia and thrombocytopenia

**Toxicity**	**Study treatment dose adjustment**
CTCAE Grade 1-2	Investigator judgement to continue treatment or if dose interruption, this should be for a maximum of 4 weeks; appropriate supportive treatment and causality investigation
CTCAE Grade 3-4	Dose interruption until recovered to CTCAE G 1 or better for a maximum of 4 weeks. If repeat CTCAE G 3-4 occurrence, dose reduce olaparib to **250 mg twice daily** as a first step and **200 mg twice daily** as a second step

Adverse event of neutropenia and leukopenia should be managed as deemed appropriate by the investigator with close follow up and interruption of study drug if CTC grade 3 or worse neutropenia occurs.

Primary prophylaxis with granulocyte colony-stimulating factor (G-CSF) is not recommended, however, if a patient develops febrile neutropenia, study treatment should be stopped and appropriate management including G-CSF should be given according to local hospital guidelines. Please note that G-CSF should not be used within at least 24 h (7 days for pegylated G-CSF) of the last dose of study treatment unless absolutely necessary.

Platelet transfusions, if indicated, should be done according to local hospital guidelines.

For cases where patients develop prolonged hematological toxicity (≥2-week interruption/delay in study treatment due to CTC grade 3 or worse), refer to guidance later in this section for the management of this.

10.6.1.1 Management of prolonged hematological toxicities while on POLAR treatment

If a patient develops prolonged hematological toxicity such as:

- ≥2 week interruption/delay in study treatment due to CTC grade 3 or worse anemia and/or development of blood transfusion dependence
- ≥2 week interruption/delay in study treatment due to CTC grade 3 or worse neutropenia (ANC < 1 x 10^9/L)
- ≥2 week interruption/delay in study treatment due to CTC grade 3 or worse thrombocytopenia and/or development of platelet transfusion dependence (Platelets < 50 x 10^9/L)

Check weekly differential blood counts including reticulocytes and peripheral blood smear. If any blood parameters remain clinically abnormal after 4 weeks of dose interruption, the patient should be referred to hematologist for further investigations. Bone marrow analysis and/or blood cytogenetic analysis should be considered at this stage according to standard hematological practice. Study treatment should be discontinued if blood counts do not recover to CTC G 1 or better within 4 weeks of dose interruption.

Development of a confirmed myelodysplastic syndrome or other clonal blood disorder should be reported as an SAE and full reports must be provided by the investigator to Merck Patient Safety. Olaparib treatment should be discontinued if patient’s diagnosis of MDS and/or AML is confirmed.

10.6.2 Management of non-hematological toxicity associated with olaparib

Repeat dose interruptions are allowed as required, for a maximum of 4 weeks on each occasion. If the interruption is any longer than this the study monitor must be informed. Where toxicity reoccurs following re-challenge with study treatment, and where further dose interruptions are considered inadequate for management of toxicity, then the patient should be considered for dose reduction or must permanently discontinue study treatment.

Study treatment can be dose reduced to 250 mg twice daily as a first step and to 200 mg twice daily as a second step. If further reduction is required, treating invesigators should follow the SOC use for Olaparib. Treatment must be interrupted if any NCI-CTCAE grade 3 or 4 adverse event occurs which the investigator considers to be related to administration of study treatment.

10.6.3 Management of new or worsening pulmonary symptom associated with olaparib

If new or worsening pulmonary symptoms (e.g., dyspnea) or radiological abnormalities occur in the absence of a clear diagnosis, an interruption in study treatment dosing is recommended and further diagnostic workup (including a high-resolution CT scan) should be performed to exclude pneumonitis.

Following investigation, if no evidence of abnormality is observed on CT imaging and symptoms resolve, then study treatment can be restarted, if deemed appropriate by the investigator. If significant pulmonary abnormalities are identified, these need to be discussed with the Study Physician.

10.6.4 Management of nausea and vomiting associated with olaparib

Events of nausea and vomiting are known to be associated with olaparib treatment. These events are generally mild to moderate (CTCAE grade 1 or 2) severity, intermittent and manageable on continued treatment. The first onset generally occurs in the first month of treatment for nausea and within the first 6 months of treatment for vomiting. For nausea, the incidence generally plateaus at around 9 months, and for vomiting at around 6 to 7 months.

No routine prophylactic anti-emetic treatment is required at the start of study treatment; however, patients should receive appropriate anti-emetic treatment at the first onset of nausea or vomiting and as required thereafter, in accordance with local treatment practice guidelines. Alternatively, olaparib tablets can be taken with a light meal/snack (i.e. 2 pieces of toast or a couple of biscuits).

As per international guidance on antiemetic use in cancer patients (NCCN), generally a single agent antiemetic should be considered e.g. dopamine receptor antagonist, antihistamines or dexamethasone.

10.6.5 Interruptions for intercurrent non-toxicity related events associated with olaparib

Study treatment dose interruption for conditions other than toxicity resolution should be kept as short as possible. If a patient cannot restart study treatment within 4 weeks for resolution of intercurrent conditions not related to disease progression or toxicity, the case should be discussed with study physician.

All dose reductions and interruptions (including any missed doses), and the reasons for the reductions/interruptions are to be recorded in the eCRF.

Study treatment should be stopped at least 3 days prior to planned surgery. After surgery study treatment can be restarted when the wound has healed. No stoppage of study treatment is required for any needle biopsy procedure.

Study treatment should be discontinued for a minimum of 3 days before a patient undergoes radiation treatment. Study treatment should be restarted within 4 weeks as long as any bone marrow toxicity has recovered.

Because the AEs related to olaparib may include asthenia, fatigue and dizziness, patients should be advised to use caution while driving or using machinery if these symptoms occur.

10.6.6 Management of renal impairment associated with olaparib

If subsequent to study entry and while still on study therapy, a patient's estimated CrCl falls below the threshold for study inclusion (≥51 ml/min), retesting should be performed promptly.

A dose reduction is recommended for patients who develop moderate renal impairment (calculated creatinine clearance by Cockcroft-Gault equation or based on a 24-hour urine test of between 31 and 50 ml/min) for any reason during the course of the study: the dose of olaparib should be reduced to 200 mg twice daily.

Because the CrCl determination is only an estimate of renal function, in instances where the CrCl falls to between 31 and 50 mL/min, the investigator should use his or her discretion in determining whether a dose change, or discontinuation of therapy is warranted.

Olaparib has not been studied in patients with severe renal impairment (creatinine clearance ≤ 30 ml/min) or end-stage renal disease; if patients develop severe impairment or end stage disease is it recommended that olaparib be discontinued.

11.0 EVALUATION DURING TREATMENT/INTERVENTION

11.1 Adverse Event (AE) Monitoring

The investigator or qualified designee will assess each participant to evaluate for potential new or worsening AEs as specified in the Trial Flow Chart and more frequently if clinically indicated. Adverse experiences will be graded and recorded throughout the study and during the follow-up period according to NCI CTCAE Version 5.0 (see Appendix 2). Toxicities will be characterized in terms regarding seriousness, causality, toxicity grading, and action taken with regard to trial treatment.

All assessments will be performed on the day of the scheduled visit date unless a time window is specified. Assessments scheduled on the days of study treatment should be performed before the infusion of study drug(s) unless otherwise noted. If the timing of a study visit coincides with a holiday, weekend, or other administrative disruption that precludes the visit, the visit should be scheduled on the nearest following feasible date, with subsequent visits rescheduled accordingly.

Table 9. Schema for treatment and evaluations.

Trial Period:	Screening Phase	Treatment Cycles (21 Day Cycles)									Pembro Q6 Wk		End of Treatment	Post-Treatment	
Treatment Cycle/Title:	Pre-screening (Visit 1)	1	2	3	4	5	6	7	8	9	Even Cycles (ex. 10, 12, 14 etc.)	Odd Cycles (ex. 11, 13, 15, etc.)	Discontinuation	Safety Follow-up (in clinic)	Survival Follow-Up (Clinic or phone)
Scheduling Window (Days):	-28		± 3	± 3	± 3	± 3	± 3	± 3	± 3	± 3	± 3	± 3	At time of Discontinuation	30 days and 60 days post discontinuation	Every 12 weeks for 5 years
**Administrative Procedures**															
Informed Consent	X														
Inclusion/Exclusion Criteria	X														
Demographics and Medical History	X														
Prior and Concomitant Medication Review	X	X	X	X	X	X	X	X	X	X	X	X**	X	X	X
Pembrolizumab** Administration		X	X	X	X	X	X	X	X	X	X	X**			
Survival Status	X	X	X	X	X	X	X	X	X	X	X	X**	X	X	X
**Clinical Procedures/Assessments**															
Review Adverse Events		X	X	X	X	X	X	X	X	X	X	X**	X	X	X
Full Physical Examination	X	X	X	X	X	X	X	X	X	X	X		X		

Trial Period:	Screening Phase	Treatment Cycles (21 Day Cycles)									Pembro Q6 Wk		End of Treatment	Post-Treatment	
Treatment Cycle/Title:	Pre-screening (Visit 1)	1	2	3	4	5	6	7	8	9	Even Cycles (ex. 10, 12, 14 etc.)	Odd Cycles (ex. 11, 13, 15, etc.)	Discontinuation	Safety Follow-up (in clinic)	Survival Follow-Up (Clinic or phone)
Scheduling Window (Days):	-28		± 3	± 3	± 3	± 3	± 3	± 3	± 3	± 3	± 3	± 3	At time of Discontinuation	30 days and 60 days post discontinuation	Every 12 weeks for 5 years
Directed Physical Examination	X	X	X	X	X	X	X	X	X	X	X		X		
Vital Signs and Weight	X	X	X	X	X	X	X	X	X	X	X		X	X	
ECOG Performance Status	X	X	X	X	X	X	X	X	X	X	X		X		
**Laboratory Procedures/Assessments: analysis performed by LOCAL laboratory**															
Pregnancy Test – Urine or Serum □-HCG	X	X													
PT/INR and aPTT	X												X		
CBC with Differential	X	X	X	X	X	X	X	X	X	X	X		X		
Comprehensive Serum Chemistry Panel	X	X	X	X	X	X	X	X	X	X	X		X		
CA 19-9, CEA	X	X	X	X	X	X	X	X	X	X	X		X		
HBsAg, IgG anti-HBs, Anti-HCV, HIV screen	X														

**Trial Period:**	**Screening Phase**	**Treatment Cycles (21 Day Cycles)**									**Pembro Q6 Wk**		**End of Treatment**	**Post-Treatment**	
Treatment Cycle/Title:	Pre-screening (Visit 1)	1	2	3	4	5	6	7	8	9	Even Cycles (ex. 10, 12, 14 etc.)	Odd Cycles (ex. 11, 13, 15, etc.)	Discontinuation	Safety Follow-up (in clinic)	Survival Follow-Up (Clinic or phone)
Scheduling Window (Days):	-28		± 3	± 3	± 3	± 3	± 3	± 3	± 3	± 3	± 3	± 3	At time of Discontinuation	30 days and 60 days post discontinuation	Every 12 weeks for 5 years
Urinalysis	X			X			X			X	X				
FT3, FT4 and TSH	X			X			X			X	X				
EKG	X			X			X			X	X				
**Efficacy Measurements**															
Tumor Imaging*	X				X			X			X*		X		
**Tumor Biopsies/Archival Tissue Collection/Correlative Studies Blood**															
Biopsy [1, 2, 3]	X			X									X		
Correlative Studies Blood Collection [4]	X			X			X				X[4]		X		
Blood draw to generate plasma for cell free (cf)DNA analysis [5]	X			X			X				X[5]		X		

**Trial Period:**	**Screening Phase**	**Treatment Cycles (21 Day Cycles)**									**Pembro Q6 Wk**		**End of Treatment**	**Post-Treatment**	
Treatment Cycle/Title:	Pre-screening (Visit 1)	1	2	3	4	5	6	7	8	9	Even Cycles (ex. 10, 12, 14 etc.)	Odd Cycles (ex. 11, 13, 15, etc.)	Discontinuation	Safety Follow-up (in clinic)	Survival Follow-Up (Clinic or phone)
Scheduling Window (Days):	-28		± 3	± 3	± 3	± 3	± 3	± 3	± 3	± 3	± 3	± 3	At time of Discontinuation	30 days and 60 days post discontinuation	Every 12 weeks for 5 years

* Computed Tomography (CT) Chest/abdomen/pelvis with oral and IV contrast or CT chest without contrast AND Magnetic Resonance Imaging (MRI) abdomen/pelvis with contrast if IV contrast/iodine allergy. Imaging frequency every 9-weeks from treatment initiation until Cycle 10, then every 12-week thereafter. If a participant has remained on study for greater than 2 years, imaging can be transitioned to every 3-6 months per MD discretion. Imaging evaluation must be done within +/- 7 days of the planned schedule. Per the treating MD's discretion, imaging can be done before the on-treatment biopsy.

** Pembrolizumab 400 mg every 6 weeks will be started on Cycle 10 Day 1. Patients will need to come to clinic during odd cycles post Cycle 10, only if clinically indicated. Per treating investigator's discretion, patients may remain at Pembrolizumab 200 mg IV every 3 weeks after the first 9 cycles. Additionally, to accomodate patients traveling far distances, if, per treating investigator, after the first or second imaging, there are no toxicities, patient's dosing of Pembrolizumab may change to 400 mg IV every 6 weeks.Telehealth visits are allowed.

1. **Pre-treatment (baseline) Tumor Tissue:** All patients must submit FFPE tumor tissue (#1 core), fresh tissue (75% of #2 core, #3, and #4 core for scRNAseq and only 25% of #2 core for organoid), and flash frozen tumor tissue (#5, 6, and any extra cores for snRNAseq, whole genome sequencing, and MIBI if possible) as per detailed biomarker management plan (BMP). The goal is to obtain 4-6 cores where clinically safe and feasible at baseline within 2-3 weeks before starting the treatment. In patients participating with major PR or CR may not have enough disease at baseline for multiple biopsies.
2. **Tumor Biopsy during Treatment (on-treatment) Period:** Repeat biopsy for on-treatment tumor tissue is requested for study purpose for patients between the 6th to 9th week (+/- 1 week) before the first repeat imaging in the 9th week. Per the treating MD's discretion, imaging can be done before the on-treatment biopsy.
3. **Tumor Biopsy at End of Treatment (on progression):** Every effort should be made to obtain EOT (+/- 28 days) biopsies in cases of RECIST v1.1 confirmed disease progression if a patient discontinues study treatment due to disease progression, except in instances where the procedure, as performed in the clinical research setting, poses an unacceptable risk to the patient.
4. **Blood draw to analyze circulating immune cell profiling:** 4 x 10ml Green-top sodium heparin tubes at 6 time points will be collected: #1 at baseline lab, #2 +/- 7 days from on-treatment biopsy date (for patients who are unable to get an on-treatment biopsy, this timepoint can be collected at C3 or C4), #3 with C6D1 lab, #4 with C12D1 lab, #5 with C18D1 lab, and #6 with EOT (+/-21 days).

5. Blood draw to generate plasma for cfDNA: TOPCOAT-POLAR will handle for MSK ACCESS #1-#6 time points for All Cohorts, we will bank them as cfDNA.

12.0 CRITERIA FOR REMOVAL FROM STUDY

12.1. Disease Progression: Patients with confirmed progressive disease following POLAR treatment will be removed from treatment, their disease pathologically documented, and the patient will be followed for survival and the immunologic endpoints. However, in the absence of unacceptable toxicity, participants who meet criteria for disease progression per RECIST 1.1 while receiving treatment will be permitted to continue treatment if in the opinion of the treating MD the patient is continuing to derive benefit from the treatment. iRECIST will be used to evaluate the response and the co-primary endpoint of overall response rate will be assessed both by RECIST and iRECIST.

12.2 Extraordinary Medical Circumstances: If at any time the constraints of this protocol are detrimental to the patient's health, the patient shall be withdrawn from treatment. In this event:

12.2.1. Document reasons for withdrawal and record whether this action was patient or physician directed.
12.2.2. The patient will remain technically on-study although treatment has been discontinued. The patients will be followed for relapse and survival.

12.3. Unexpected or Life-Threatening Toxicity (CTCAE v5.0 Table):
12.3.1. Direct questions regarding drug therapy to the Principal Investigator.
12.3.2. Reporting requirements and procedures depend upon:
(1) whether agents are suspected of causing toxicity, (2) whether possibility of such toxicity was reported in the consent form, or manufacturer's literature (Published Toxicity), (3) the severity or grade of the toxicity.

12.3.3. Expected toxicities:
12.3.3.1. Grade 4 myelosuppression: Report only as part of regular data submission.
12.3.3.2. All other toxicities, Grades 4: Written Report to Principal and Co-Investigators, within 10 working days.
12.3.3.3. All other toxicities, Grades 1 to 3: Report only as part of regular data submission.

12.3.4. Unexpected Toxicities: Unexpected toxicities are toxicities that are not listed in the toxicity management section of the protocol, the consent form, or the manufacturer's package inserts. All unexpected toxicities will be reported to Principal and Co-Investigators, within 10 working days.

12.4. Patient noncompliance with the protocol requirements

12.5. Patient request to withdraw

12.6. Development of serious intercurrent illness during treatment

12.7. Patient removed at the discretion of the PI

13.0 CRITERIA FOR OUTCOME ASSESSMENT AND ENDPOINT EVALUABILITY

13.1. Tumor Measurements

Response Evaluation Criteria in Solid Tumors (RECIST) version 1.1 will be used to determine treatment response. Baseline tumor measurements will be performed prior to initiation of therapy. These measurements should be done according to iRECIST (see Appendix 2) [54]. An estimate of overall objective and subjective response will be made and recorded at the end of treatment at 8 weeks after the last infusion. Since it is expected that immune activity would lead to tumor flare, the restaging CT scans may be repeated 8 weeks later based on irRECIST and the designation of iCR, iPR, iSD, iUPD, or iCPD will be made at that time. Best overall response will be used.

13.1.2. Disease control rate (DCR): DCR is defined as the percentage of patients who have achieved complete response, partial response, and stable disease to the study treatment.

13.1.3. Overall response rate (ORR): ORR is defined as the percentage of patients who have achieved complete response and partial response to the study treatment.

13.1.4. Best Overall Response (BOR): BOR is defined as the best response recorded from the start of the study treatment until the disease progression/recurrence.

13.1.5. Durable response rate (DRR): DRR is defined as the percentage of patients who had achieved continued disease responses (CR and PR) for more than 6 months.

13.1.6. Durable disease control rate (DDCR): DDCR is defined as the percentage of patients who had achieved continued disease controls (CR, PR, and SD) for more than 6 months from the time from the initiation of study treatment.

13.1.7. Additional Assessments. All patients will have a baseline tumor marker assessment and testing of tumor markers at the designated follow-up time points. Changes in serial tumor marker measurements, in those patients who have baseline elevations, will be utilized as a surrogate marker of clinical response, stable disease or disease progression and reported as such.

13.1.8. Patients without residual disease: For patients whose primary tumor or metastatic disease has been removed or successfully treated, respectively, prior to entry into this trial, progression free survival will be evaluated based on duration of relapse-free survival.

13.1.9. Progression free survival (PFS): PFS is defined as the time from the initiation of study treatment to radiographic progression or death.

13.1.10. Overall survival (OS): OS is defined as the time from the initiation of study treatment to the date of death from any cause.

13.2 Criteria for Study Endpoint Evaluability

- All patients who receive at least one dose of olaparib and pembrolizumab are considered to be evaluable for toxicity.

- All patients who receive at least olaparib for 14 days of 21-day course and one dose of pembrolizumab are considered to be evaluable for efficacy evaluation.

- Patients who enroll with a complete response on platinum-based treatment are evaluable and a radiographical disease recurrence by RECIST v 1.1 will be considered a progression.

- Patients who are screened eligible but subsequently withdraw from the study prior to receiving pembrolizumab with olaparib are considered inevaluable and will be replaced on the study.

14.0 BIOSTATISTICS

This is a phase 2, open-label, non-randomized study evaluating the novel combination of pembrolizumab and olaparib (POLAR) in three cohorts of metastatic PDAC s with homologous recombination deficiency (HRD) in the first-line or second-line on-platinum maintenance setting, two of which are genomically enriched and one is identified by platinum sensitivity. The primary objective of the study is to determine both the 6-month PFS rate by RECIST v1.1 as well as the objective response rate (ORR, CR+PR) by iRECIST for this combination. Both factors will be considered for purposes of sample size determination as follows and a two-stage design will be employed. The secondary endpoints of this study are overall survival (OS), progression-free survival (PFS) by both RECIST v1.1 and iRECIST, disease control rate (DCR) by both RECIST v1.1 and iRECIST, CA 19-9, and CEA response. Progression-free survival (PFS) will be analyzed from both the consent date and treatment start date (C1D1), separately, due to the maintenance trial design. Exploratory scientific correlates include assessments of HRD genetic mutational signatures, neoantigens, immunogenic tumor microenvironment,clonal evolutionary dynamics to study heterogeneity, andresistance mechanism to POLAR. Exploratory endpoints will also include second progression-free survival (PFS2) from time of subsequent systemic treatment start post-POLAR to progression on that treatment as well as assess duration of the first-line or second-line treatment prior to POLAR treatment. Exploratory endpoints will also assess genetic signatures and compare clinical outcomes, evaluate reversion mutation, zygosity, plasticity and aneuploidy from tumor and ctDNA, and to assess the role of maintenance treatment with minimally residual disease by the presence of ctDNA and CA 19-9 and its trend prior to treatment.

A two stage design will be employed only in Cohort A with co-primary endpoints ORR by iRECIST and 6-month PFS rate[7] by RECIST v1.1. An initial cohort of 20 patients will be treated. If 14 or more patients are alive and progression free at the 6 months follow-up or if 6 or more objective responses are observed, an additional 13 patients will be accrued for a total of 33 patients. If 23 or more of the 33 patients are alive and progression free at 6 months from start of treatment or if 12 or more objective responses are observed, we can conclude that the regimen is sufficiently active to warrant further testing in this group of patients. We will also evaluate the progression-free survival from the consent date secondarily to the treatment start date (C1D1). We used the reference from historical data for these co-primary endpoints. 6-months PFS rate was 50% and ORR was 20% in single-agent olaparib study arm compared to placebo control arm in maintenance setting[7].This design yields 81% power to detect a true 6-month PFS rate of 77% or more and it yields 81% power to detect a true ORR of at least 43%. It yields approximately 0.95 probability of a negative result if the true 6-month PFS rate is no more than 53% and the true ORR is no more than 20%. The type I error for the ORR endpoint is 0.02 and the type I error for the PFS endpoint is 0.028 and so the overall type I error of the composite design is bounded at 0.05 (the sum of the two type I error rates for PFS and RR) while the type II error rate for the composite design is 0.09 (each design has a type II error rate of 0.09). The probability of early termination was calculated as the product of the probability of early termination for the PFS endpoint (0.904) and the probability of early termination for the RR endpoint (0.804), resulting in a probability of early termination of 0.73 for the composite endpoint. These calculations assume that ORR and PFS are independent, , the type 1 error slightly decreases if ORR and PFS are positively correlated, which is very likely. The calculations also assume that the 6 month PFS endpoint is binary. Patients who are lost to follow up before their 6 months PFS

evaluation and who did not respond or do not have documented response before their being lost to follow up will be counted as events (nonresponders/progressors) for the composite primary endpoint. However this will be uncommon. If after the first stage we meet the rule of observing 6 or more responses out of the 20 patients enrolled in the first stage, then no waiting is required and we can proceed with enrollment to the second stage. However, if we do not observe 6 out of 20 responses after the first stage, we will halt accrual after the first 20 patients until we obtain the 6-month PFS status for these 20 patients. The interim look for Cohort A will not affect accrual to Cohorts B and C.

Cohorts B and C will be exploratory and will enroll 15 patients each. ORR will be estimated using the binomial distribution along with exact 95% confidence intervals. With 15 accrued patients, we can estimate the DCR to within +/- 25% margins of error with 95% confidence.

Secondary objectives

Adverse events will be summarized descriptively and tabulated separately for each cohort. DCR will be estimated using binomial proportions along with exact 95% confidence intervals while PFS and OS will be estimated using the Kaplan-Meier method separately for each of the 3 cohorts. CEA and CA 19-9 will be summarized using medians and interquartile range.

Exploratory objectives

Wilcoxon rank sum test will be used to assess associations between genetic and transcriptomic signatures at baseline with response while Cox regression will be used to assess associations with PFS and OS. These analyses will be conducted separately for each cohort acknowledging the exploratory nature of these analyses particularly for Cohorts B and C which have limited sample size. Changes between baseline and after treatment in neoantigen induction, TME, and immunomodulatory targets will be summarized descriptively using medians and interquartile range for each cohort separately.To understand the resistance mechanisms, post-treatment changes (4-6 weeks and at progression) of tumor samples and circulating tumor DNA (MSK-ACCESS) from baseline will be associated with response using Wilcoxon rank sum test while Cox regression will be used for PFS and OS outcomes.Finally, in selected patients with viable organoids available from IRB# 15-149 prior to enrollment to POLAR trial, we will concurrently evaluate the response of organoid to POLAR in real time as participants are treated in parallel. Dr. Iacobuzio will oversee this part. PFS2 will be estimated using the Kaplan-Meier method. Associations between genetic mutations (HRD and non-HRD) and outcome will be assessed using Fisher's exact test for response and log-rank test for survival outcomes. Biomarkers (reversion mutation, zygosity, plasticity, aneuploidy from tumor, and circulating tumor DNA) will be summarized using proportions. Presence of circulating tumor DNA and levels of CA 19-9 prior to treatment at time points prior to consent and between the consent and start date will be graphically summarized at each timepoint.

14.1 Populations for Analyses

- **Safety Analysis Dataset:** Safety analyses will be conducted for all evaluable participants who took at least one dose of study intervention.

- **Primary Efficacy Dataset**: participants who receive at least olaparib for 14 days of 21-day course and one dose of pembrolizumab are considered to be evaluable for efficacy evaluation.

15.0 TOXICITIES/RISKS/SIDE EFFECTS

The investigators will assess each participant to evaluate for potential new or worsening AEs as specified in the Trial Flow Chart and more frequently if clinically indicated. Adverse experiences will be graded and recorded throughout the study and during the follow-up period according to NCI CTCAE Version 5 (see Appendix 2). Toxicities will be characterized in terms regarding seriousness, causality, toxicity grading, and action taken with regard to trial treatment.

An adverse event is defined as any untoward medical occurrence in a patient or clinical investigation participant administered a pharmaceutical product and which does not necessarily have to have a causal relationship with this treatment. An adverse event can therefore be any unfavorable and unintended sign (including an abnormal laboratory finding, for example), symptom, or disease temporally associated with the use of a medicinal product or protocol-specified procedure, whether or not considered related to the medicinal product or protocol-specified procedure. Any worsening (i.e., any clinically significant adverse change in frequency and/or intensity) of a preexisting condition that is temporally associated with the use of the Merck's product, is also an adverse event.

Changes resulting from normal growth and development that do not vary significantly in frequency or severity from expected levels are not to be considered adverse events. Examples of this may include, but are not limited to, teething, typical crying in infants and children and onset of menses or menopause occurring at a physiologically appropriate time.

Adverse events may occur during the course of the use of POLAR (Merck product of pembrolizumab and olaparib) in clinical trials, or as prescribed in clinical practice, from overdose (whether accidental or intentional), from abuse and from withdrawal.

All AEs, SAEs and other reportable safety events that occur after the consent form is signed but before treatment allocation/randomization must be reported by the investigator if the participant is receiving placebo run-in or other run-in treatment, if the event cause the participant to be excluded from the study, or is the result of a protocol-specified intervention, including but not limited to washout or discontinuation of usual therapy, diet, or a procedure.

If a patient requires discontinuation of either olaparib or pembrolizumab for reasons of toxicity and if in the opinion of the treating MD the patient is continuing to derive benefit, the patient is able to continue on study.

- All AEs from the time of treatment allocation/randomization through 30 days following cessation of study treatment must be reported by the investigator.

- All AEs meeting serious criteria, from the time of treatment allocation/randomization through 90 days following cessation of study treatment, or 30 days following cessation of study treatment if the participant initiates new anticancer therapy, whichever is earlier must be reported by the investigator.

- All pregnancies and exposure during breastfeeding, from the time of treatment allocation/randomization through 120 days following cessation of study treatment, or 30 days

following cessation of study treatment if the participant initiates new anticancer therapy must be reported by the investigator.

● Additionally, any SAE brought to the attention of an investigator at any time outside of the time period specified above must be reported immediately by the investigator if the event is considered to be drug-related.

Investigators are not obligated to actively seek AE or SAE or other reportable safety events in former study participants. However, if the investigator learns of any SAE, including a death, at any time after a participant has been discharged from the study, and he/she considers the event to be reasonably related to the study treatment or study participation, the investigator must promptly notify Merck.

Possible Side Effects of pembrolizumab

**COMMON, SOME MAY BE SERIOUS**<br>In 100 people receiving pembrolizumab more than 20 and up to 100 may have:
• Itching of the skin<br>• Loose or watery stools<br>• Cough

**OCCASIONAL, SOME MAY BE SERIOUS**<br>In 100 people receiving pembrolizumab from 4 to 20 may have:
• Joint pain<br>• Rash<br>• Fever<br>• Back pain<br>• Pain in the belly<br>• Loss of skin color<br>• Not enough thyroid hormone (hypothyroidism), which may make you feel tired, gain weight, feel cold, or have infrequent or hard stools<br>• Low levels of salt in the blood (hyponatremia), which may make you feel tired, feel confused, have a headache, have muscle cramps, and/or feel sick to your stomach<br>• Inflammation of the lungs (pneumonitis), which may make you feel short of breath and cough<br>• Too much thyroid hormone (hyperthyroidism), which may make you feel anxious, feel angry, have trouble sleeping, feel weak, tremble, sweat, feel tired, or have loose and watery stools<br>• Infusion-related reaction (IRR; like an allergic reaction), which may make you feel dizzy or faint, feel flushed, get a rash, have a fever, feel short of breath, experience a decrease in your blood pressure, or have pain at the infusion site, either while you are receiving the infusion or just after<br>• Inflammation of the bowels/gut (colitis), which may cause severe pain in your belly, with loose or watery stools, and black, tarry, sticky stools, or stools with blood or mucus

• Severe inflammation of the skin (Stevens-Johnson syndrome and/or toxic epidermal necrolysis), which may cause peeling of the skin, itchiness, and/or skin redness. The skin inflammation could be widespread throughout your body. More severe skin reactions may involve the inside of your mouth, the surface of your eyes and genital areas, and/or may cause the top layer of skin to peel from all over your body, which can cause a severe infection.

**RARE, AND SERIOUS**<br>In 100 people receiving pembrolizumab 3 or fewer may have:
• Inflammation of the nerves (Guillain-Barré syndrome), which may cause pain, weakness or tingling in your hands and feet, and may spread to your legs, arms, and upper body, leading to severe muscle weakness and possible temporary paralysis<br>• Inflammation of the muscles (myositis), which may make you feel weak or have pain in your muscles<br>• Inflammation of the pancreas (a gland in your abdomen that controls sugar levels; pancreatitis), which may cause severe pain in the top part of your belly, and the pain may move to your back; you may feel sick to your stomach, and experience vomiting that gets worse when you eat<br>• Inflammation of the eye (uveitis), which may cause eye redness, blurred vision, sensitivity to light, eye pain, headaches, or seeing “floaters”<br>• Inflammation of the liver (hepatitis), which may make you feel sick to your stomach and vomit, feel like not eating, feel tired, have a mild fever, have a pain in the right side of your belly, yellow eyes and skin, and dark urine<br>• Inflammation of the pituitary gland (a gland in the head; hypophysitis), which may make you feel sick to your stomach or have headaches, changes in your behavior, double vision, few to no menstrual cycles, weakness, vomiting, and dizziness or fainting<br>• Adrenal glands (glands on top of the kidneys) may not make enough hormone (adrenal insufficiency), which could cause tiredness; weight loss; muscle weakness; feeling faint; having joint, muscle, and belly aches; nausea; vomiting; and loose or watery stools; fever; salt craving; and sometimes darkening of the skin like a suntan<br>• Type 1 diabetes, a condition that can result in too much sugar in your blood, making you feel thirstier than usual, with frequent urination, and weight loss. You are likely to need regular insulin shots to treat this condition.<br>• Inflammation of the kidney (nephritis), which may make you pass less urine or have cloudy or bloody urine, swelling, and low back pain<br>• Inflammation of the middle layer of your heart wall (myocarditis), which may make it difficult for your heart to pump blood through your body, causing chest pain, shortness of breath, and swelling of the legs. You may experience a fast or irregular heartbeat that may cause dizziness or fainting.<br>• Inflammation of the thyroid gland, which may lead to changes in your heart rate, blood pressure, body temperature, and the rate at which food is converted into energy (thyroiditis)<br>• Myasthenic syndrome/myasthenia gravis including exacerbation, conditions that may make you feel weak and tired, and may cause drooping eyelids, blurred or double vision, difficulty swallowing, slurred speech, weakness in your arms and legs, or difficulty breathing

- Sarcoidosis, the formation of small clusters of immune cells (granulomas) in parts of your body, such as your lymph nodes, eyes, skin, or lungs
- Inflammation of the brain (encephalitis), with confusion and fever. Symptoms may include disorientation, memory problems, seizures (fits), changes in personality and behavior, difficulty speaking, weakness or loss of movement in some parts of your body, and loss of consciousness
- Inflammation of the spinal cord (myelitis), which may cause pain, numbness, tingling, or weakness in the arms or legs, bladder or bowel problems including needing to urinate more frequently, urinary incontinence, difficulty urinating, and constipation

Possible Side Effects of olaparib

**COMMON, SOME MAY BE SERIOUS**<br>In 100 people receiving olaparib more than 20 and up to 100 may have:
• Feeling sick to your stomach (nausea)<br>• Vomiting<br>• Tiredness/weakness (fatigue)<br>• Indigestion/heartburn (dyspepsia)<br>• Loss of appetite<br>• Headache<br>• Changes in the way food tastes (dysgeusia)<br>• Dizziness<br>• Loose or watery stools (diarrhea): Call the study doctor right away if you have continuous or severe diarrhea<br>• Cough<br>• Shortness of breath (dyspnea)<br>• Decrease in the number of red blood cells (anemia), which can be associated with shortness of breath, fatigue, pale skin, or fast heartbeat, and may require a transfusion for treatment<br>• Decrease in the total number of white blood cells (leukopenia) and in white blood cells called neutrophils (neutropenia) that protect you from infection, which can be associated with fever

**OCCASIONAL, SOME MAY BE SERIOUS**<br>In 100 people receiving olaparib from 4 to 20 may have:
• Sore mouth (stomatitis)<br>• Skin rash<br>• Increase in the level of creatinine in the blood. This test result can suggest a potential problem with your kidneys. Symptoms of kidney damage include decreased urine output, swelling of your legs, ankles, and feet from retaining fluids, unexplained shortness of breath, fatigue, nausea, confusion, and chest pain.<br>• Pain in the stomach area under the ribs (upper abdominal pain)

**RARE, AND SERIOUS**<br>In 100 people receiving olaparib 3 or fewer may have:

• Allergic reactions; symptoms include tingling or itching in the mouth, hives, swelling of the lips, face tongue, throat, and other parts of the body, wheezing, nasal congestion, trouble breathing, abdominal pain, diarrhea, nausea, or vomiting<br>• Itchy rash or swollen, reddened skin (dermatitis)<br>• Increase in the size of red blood cells (not associated with any symptoms); this condition is treatable but, in rare cases, a blood transfusion may be required

15.1 Serious Adverse Event (SAE) Reporting

- An adverse event is considered serious if it results in ANY of the following outcomes: Death
- A life-threatening adverse event
- An adverse event that results in inpatient hospitalization or prolongation of existing hospitalization
- A persistent or significant incapacity or substantial disruption of the ability to conduct normal life functions
- A congenital anomaly/birth defect
- Important Medical Events (IME) that may not result in death, be life threatening, or require hospitalization may be considered serious when, based upon medical judgment, they may jeopardize the patient or participant and may require medical or surgical intervention to prevent one of the outcomes listed in this definition
- An overdose

Note: Hospital admission for a planned procedure/disease treatment is not considered an SAE.

Refer to Table 3 for additional details regarding each of the above criteria.

SAE reporting is required as soon as the participant starts investigational treatment/intervention. SAE reporting is required for 30-days after the participant's last investigational treatment/intervention. Any event that occur after the 30-day period that is unexpected and at least possibly related to protocol treatment must be reported.

Please note: Any SAE that occurs prior to the start of investigational treatment/intervention and is related to a screening test or procedure (i.e., a screening biopsy) must be reported.

All SAEs must be submitted in PIMS. If an SAE requires submission to the HRPP office per IRB SOP RR-408 'Reporting of Serious Adverse Events', the SAE report must be submitted within 5 calendar days of the event. All other SAEs must be submitted within 30 calendar days of the event.

The report should contain the following information:

- The date the adverse event occurred
- The adverse event
- The grade of the event
- Relationship of the adverse event to the treatment(s)
- If the AE was expected

- Detailed text that includes the following
- An explanation of how the AE was handled
- A description of the participant's condition
- Indication if the participant remains on the study
- If an amendment will need to be made to the protocol and/or consent form
- If the SAE is an Unanticipated Problem

15.2 Evaluating Adverse Events

An investigator who is a qualified physician will evaluate all adverse events according to the NCI Common Terminology for Adverse Events (CTCAE), version 5.0. Any adverse event which changes CTCAE grade over the course of a given episode will have each change of grade recorded on the adverse event case report forms/worksheets.

All adverse events regardless of CTCAE grade must also be evaluated for seriousness.

An investigator who is a qualified physician, will evaluate all adverse events. Table 3. Evaluating Adverse Events

16.0 PROTECTION OF HUMAN PARTICIPANTS

16.1 Privacy

MSK's Privacy Office may allow the use and disclosure of protected health information pursuant to a completed and signed Research Authorization form. The use and disclosure of protected health information will be limited to the individual patients/entities described in the Research Authorization form. A Research Authorization form must be approved by the IRB and Privacy Board (IRB/PB).

The consent indicates that patients de identified information collected for the purposes of this study may be shared with other qualified researchers. Only researchers who have received approval from MSK will be allowed to access this information which will not include protected health information, such as the participant's name, except for dates. It is also stated in the Research Authorization that their research data may be shared with others at the time of study publication.

16.2 Data Management

The clinical trial data will be collected by data manager and through dedicated clinical research assistant (CRA). All the cases will be discussed in detail at our pancreatic cancer weekly clinical research meetings at MSK. A dedicated CRA will store the data using Medidata program with secured password at protected drive at pancreatic cancer center hard drive for which only personnel associated with the study will have the access. Final data sets for publication are required to be locked and stored centrally for potential future access requests from outside entities.

16.3 Quality Assurance

Quality assurance will be strictly managed and monitored at pancreatic cancer weekly clinical research meetings. At these meetings, pancreas-dedicated GI oncologists will be present and

protocol compliance, eligibility verification, informed consent procedure and data accuracy will be addressed.

16.4 Data and Safety Monitoring

The Data and Safety Monitoring Plan utilized for this study must align with the MSK DSM Plan, where applicable.

The Data and Safety Monitoring (DSM) Plans at Memorial Sloan Kettering were approved by the National Cancer Institute in August 2018. The plans address the new policies set forth by the NCI in the document entitled "Policy of the National Cancer Institute for Data and Safety Monitoring of Clinical Trials."

There are several different mechanisms by which clinical studies are monitored for data, safety and quality. At a departmental/PI level there exists procedures for quality control by the research team(s). Institutional processes in place for quality assurance include protocol monitoring, compliance and data verification audits, staff education on clinical research QA and two institutional committees that are responsible for monitoring the activities of our clinical trials programs. The committees: *Data and Safety Monitoring Committee (DSMC)* for Phase I and II clinical trials, and the *Data and Safety Monitoring Board (DSMB)* for Phase III clinical trials, report to the Deputy Physician-in-Chief, Clinical Research.

During the protocol development and review process, each protocol will be assessed for its level of risk and degree of monitoring required.

The MSK DSMB monitors phase III trials and the DSMC monitors non-phase III trials. The DSMB/C have oversight over the following trials:

- MSK Investigator Initiated Trials (IITs; MSK as sponsor)
- External studies where MSK is the data coordinating center
- Low risk studies identified as requiring DSMB/C review

The DSMC will initiate review following the enrollment of the first participant/or by the end of the year one if no accruals and will continue for the study lifecycle until there are no participants under active therapy and the protocol has closed to accrual. The DSMB will initiate review once the protocol is open to accrual.

17.0 REFERENCES

1. Ott PA, Bang YJ, Piha-Paul SA, et al. T-Cell-Inflamed Gene-Expression Profile, Programmed Death Ligand 1 Expression, and Tumor Mutational Burden Predict Efficacy in Patients Treated With Pembrolizumab Across 20 Cancers: KEYNOTE-028. J Clin Oncol 2019;37:318-27.
2. Royal RE, Levy C, Turner K, et al. Phase 2 trial of single agent Ipilimumab (anti-CTLA-4) for locally advanced or metastatic pancreatic adenocarcinoma. J Immunother 2010;33:828-33.
3. O'Reilly EM, Oh DY, Dhani N, et al. Durvalumab With or Without Tremelimumab for Patients With Metastatic Pancreatic Ductal Adenocarcinoma: A Phase 2 Randomized Clinical Trial. JAMA Oncol 2019.
4. Fong PC, Boss DS, Yap TA, et al. Inhibition of poly(ADP-ribose) polymerase in tumors from BRCA mutation carriers. N Engl J Med 2009;361:123-34.

5. Robson M, Im SA, Senkus E, et al. Olaparib for Metastatic Breast Cancer in Patients with a Germline BRCA Mutation. N Engl J Med 2017;377:523-33.
6. Moore K, Colombo N, Scambia G, et al. Maintenance Olaparib in Patients with Newly Diagnosed Advanced Ovarian Cancer. N Engl J Med 2018;379:2495-505.
7. Golan T, Hammel P, Reni M, et al. Maintenance Olaparib for Germline BRCA-Mutated Metastatic Pancreatic Cancer. N Engl J Med 2019.
8. Kaufman B, Shapira-Frommer R, Schmutzler RK, et al. Olaparib monotherapy in patients with advanced cancer and a germline BRCA1/2 mutation. J Clin Oncol 2015;33:244-50.
9. Lord CJ, Ashworth A. BRCAness revisited. Nat Rev Cancer 2016;16:110-20.
10. Turner N, Tutt A, Ashworth A. Hallmarks of 'BRCAness' in sporadic cancers. Nat Rev Cancer 2004;4:814-9.
11. Helleday T. The underlying mechanism for the PARP and BRCA synthetic lethality: clearing up the misunderstandings. Mol Oncol 2011;5:387-93.
12. Lord CJ, Tutt AN, Ashworth A. Synthetic lethality and cancer therapy: lessons learned from the development of PARP inhibitors. Annu Rev Med 2015;66:455-70.
13. Lowery MA, Jordan EJ, Basturk O, et al. Real-Time Genomic Profiling of Pancreatic Ductal Adenocarcinoma: Potential Actionability and Correlation with Clinical Phenotype. Clin Cancer Res 2017;23:6094-100.
14. Waddell N, Pajic M, Patch AM, et al. Whole genomes redefine the mutational landscape of pancreatic cancer. Nature 2015;518:495-501.
15. Bailey P, Chang DK, Nones K, et al. Genomic analyses identify molecular subtypes of pancreatic cancer. Nature 2016;531:47-52.
16. Moffitt RA, Marayati R, Flate EL, et al. Virtual microdissection identifies distinct tumor- and stroma-specific subtypes of pancreatic ductal adenocarcinoma. Nat Genet 2015;47:1168-78.
17. Lowery MA, Wong W, Jordan EJ, et al. Prospective Evaluation of Germline Alterations in Patients With Exocrine Pancreatic Neoplasms. J Natl Cancer Inst 2018;110:1067-74.
18. Jonsson P, Bandlamudi C, Cheng ML, et al. Tumour lineage shapes BRCA-mediated phenotypes. Nature 2019;571:576-9.
19. O'Reilly EM, Lee JW, Lowery MA, et al. Phase 1 trial evaluating cisplatin, gemcitabine, and veliparib in 2 patient cohorts: Germline BRCA mutation carriers and wild-type BRCA pancreatic ductal adenocarcinoma. Cancer 2018;124:1374-82.
20. Lowery MA, Kelsen DP, Capanu M, et al. Phase II trial of veliparib in patients with previously treated BRCA-mutated pancreas ductal adenocarcinoma. Eur J Cancer 2018;89:19-26.
21. O’Reilly EM, Lee JW, Zalupski M, et al. Randomized, Multicenter, Phase II Trial of Gemcitabine and Cisplatin With or Without Veliparib in Patients With Pancreas Adenocarcinoma and a Germline BRCA/PALB2 Mutation.0:JCO.19.02931.
22. Conroy T, Desseigne F, Ychou M, et al. FOLFIRINOX versus gemcitabine for metastatic pancreatic cancer. N Engl J Med 2011;364:1817-25.
23. Park W, Wong W, Yu KH, et al. Homologous recombination deficiency (HRD): A biomarker for first-line (1L) platinum in advanced pancreatic ductal adenocarcinoma (PDAC). 2019;37:4132-.
24. Park W, Chen J, Chou JF, et al. Genomic Methods Identify Homologous Recombination Deficiency in Pancreas Adenocarcinoma and Optimize Treatment Selection. Clinical cancer research : an official journal of the American Association for Cancer Research 2020;Accepted April 8, 2020.
25. Disis ML. Immune regulation of cancer. J Clin Oncol 2010;28:4531-8.
26. Riaz N, Blecua P, Lim RS, et al. Pan-cancer analysis of bi-allelic alterations in homologous recombination DNA repair genes. Nat Commun 2017;8:857.
27. Pantelidou C, Sonzogni O, de Oliveira Taveira M, et al. PARP inhibitor efficacy depends on CD8+ T cell recruitment via intratumoral STING pathway activation in BRCA-deficient models of triple-negative breast cancer. 2019:CD-18-1218.
28. Greenwald RJ, Freeman GJ, Sharpe AH. The B7 family revisited. Annu Rev Immunol 2005;23:515-48.

29. Okazaki T, Maeda A, Nishimura H, Kurosaki T, Honjo T. PD-1 immunoreceptor inhibits B cell receptor-mediated signaling by recruiting src homology 2-domain-containing tyrosine phosphatase 2 to phosphotyrosine. Proc Natl Acad Sci U S A 2001;98:13866-71.
30. Zhang X, Schwartz JC, Guo X, et al. Structural and functional analysis of the costimulatory receptor programmed death-1. Immunity 2004;20:337-47.
31. Sheppard KA, Fitz LJ, Lee JM, et al. PD-1 inhibits T-cell receptor induced phosphorylation of the ZAP70/CD3zeta signalosome and downstream signaling to PKCtheta. FEBS Lett 2004;574:37-41.
32. Chemnitz JM, Parry RV, Nichols KE, June CH, Riley JL. SHP-1 and SHP-2 associate with immunoreceptor tyrosine-based switch motif of programmed death 1 upon primary human T cell stimulation, but only receptor ligation prevents T cell activation. J Immunol 2004;173:945-54.
33. Francisco LM, Sage PT, Sharpe AH. The PD-1 pathway in tolerance and autoimmunity. Immunol Rev 2010;236:219-42.
34. Parry RV, Chemnitz JM, Frauwirth KA, et al. CTLA-4 and PD-1 receptors inhibit T-cell activation by distinct mechanisms. Mol Cell Biol 2005;25:9543-53.
35. Telli ML, Stover DG, Loi S, et al. Homologous recombination deficiency and host anti-tumor immunity in triple-negative breast cancer. Breast Cancer Res Treat 2018;171:21-31.
36. Vergote I, Sehouli J, Salutari V, et al. ENGOT-OV43/KEYLYNK-001: A phase III, randomized, double-blind, active- and placebo-controlled study of pembrolizumab plus chemotherapy with olaparib maintenance for first-line treatment of BRCA-nonmutated advanced epithelial ovarian cancer. 2019;37:TPS5603-TPS.
37. Konstantinopoulos PA, Waggoner S, Vidal GA, et al. Single-Arm Phases 1 and 2 Trial of Niraparib in Combination With Pembrolizumab in Patients With Recurrent Platinum-Resistant Ovarian Carcinoma. JAMA Oncology 2019;5:1141-9.
38. Cronin KA, Lake AJ, Scott S, et al. Annual Report to the Nation on the Status of Cancer, part I: National cancer statistics. Cancer 2018.
39. Rahib L, Smith BD, Aizenberg R, Rosenzweig AB, Fleshman JM, Matrisian LM. Projecting cancer incidence and deaths to 2030: the unexpected burden of thyroid, liver, and pancreas cancers in the United States. Cancer Res 2014;74:2913-21.
40. Dahan L, Phelip JM, Malicot KL, et al. FOLFIRINOX until progression, FOLFIRINOX with maintenance treatment, or sequential treatment with gemcitabine and FOLFIRI.3 for first-line treatment of metastatic pancreatic cancer: A randomized phase II trial (PRODIGE 35-PANOPTIMOX). 2018;36:4000-.
41. Hellmann MD, Ciuleanu TE, Pluzanski A, et al. Nivolumab plus Ipilimumab in Lung Cancer with a High Tumor Mutational Burden. N Engl J Med 2018;378:2093-104.
42. Le DT, Durham JN, Smith KN, et al. Mismatch repair deficiency predicts response of solid tumors to PD-1 blockade. Science 2017;357:409-13.
43. Samstein RM, Lee CH, Shoushtari AN, et al. Tumor mutational load predicts survival after immunotherapy across multiple cancer types. Nature genetics 2019;51:202-6.
44. Balachandran VP, Luksza M, Zhao JN, et al. Identification of unique neoantigen qualities in long-term survivors of pancreatic cancer. Nature 2017;551:512-6.
45. Pishvaian MJ, Blais EM, Brody JR, et al. Outcomes in Patients With Pancreatic Adenocarcinoma With Genetic Mutations in DNA Damage Response Pathways: Results From the Know Your Tumor Program. 2019:1-10.
46. Pishvaian MJ, Blais EM, Brody JR, et al. Overall survival in patients with pancreatic cancer receiving matched therapies following molecular profiling: a retrospective analysis of the Know Your Tumor registry trial. Lancet Oncol 2020;21:508-18.
47. Samstein RM, Lee CH, Shoushtari AN, et al. Tumor mutational load predicts survival after immunotherapy across multiple cancer types. Nat Genet 2019.
48. Terrero G, Pollack T, Sussman DA, Lockhart AC, Hosein PJ. Exceptional responses to ipilimumab/nivolumab (ipi/nivo) in patients (pts) with refractory pancreatic ductal adenocarcinoma (PDAC) and germline BRCA or RAD51 mutations. 2020;38:754-.

49. Pantelidou C, Sonzogni O, De Oliveria Taveira M, et al. PARP Inhibitor Efficacy Depends on CD8(+) T-cell Recruitment via Intratumoral STING Pathway Activation in BRCA-Deficient Models of Triple-Negative Breast Cancer. Cancer Discov 2019;9:722-37.
50. Shroff RT, Hendifar A, McWilliams RR, et al. Rucaparib Monotherapy in Patients With Pancreatic Cancer and a Known Deleterious BRCA Mutation. JCO Precis Oncol 2018;2018.
51. Sen T, Rodriguez BL, Chen L, et al. Targeting DNA Damage Response Promotes Antitumor Immunity through STING-Mediated T-cell Activation in Small Cell Lung Cancer. Cancer Discov 2019;9:646-61.
52. Huang J, Wang L, Cong Z, et al. The PARP1 inhibitor BMN 673 exhibits immunoregulatory effects in a Brca1(-/-) murine model of ovarian cancer. Biochem Biophys Res Commun 2015;463:551-6.
53. Riaz N, Havel JJ, Makarov V, et al. Tumor and Microenvironment Evolution during Immunotherapy with Nivolumab. Cell 2017;171:934-49.e16.
54. Seymour L, Bogaerts J, Perrone A, et al. iRECIST: guidelines for response criteria for use in trials testing immunotherapeutics. Lancet Oncol 2017;18:e143-e52.

18.0 APPENDICES

Appendix 1: ECOG Performance Status

Grade	Description
0	Normal activity. Fully active, able to carry on all pre-disease performance without restriction.
1	Symptoms, but ambulatory. Restricted in physically strenuous activity, but ambulatory and able to carry out work of a light or sedentary nature (e.g., light housework, office work).
2	In bed <50% of the time. Ambulatory and capable of all self-care, but unable to carry out any work activities. Up and about more than 50% of waking hours.
3	In bed >50% of the time. Capable of only limited self-care, confined to bed or chair more than 50% of waking hours.
4	100% bedridden. Completely disabled. Cannot carry on any self-care. Totally confined to bed or chair.
5	Dead.

* As published in Am. J. Clin. Oncol.: *Oken, M.M., Creech, R.H., Tormey, D.C., Horton, J., Davis, T.E., McFadden, E.T., Carbone, P.P.: Toxicity And Response Criteria Of The Eastern Cooperative Oncology Group. Am J Clin Oncol 5:649-655, 1982.* The Eastern Cooperative Oncology Group, Robert Comis M.D., Group Chair.

Appendix 2: Common Terminology Criteria for Adverse Events V5.0 (CTCAE)

The descriptions and grading scales found in the revised NCI Common Terminology Criteria for Adverse Events (CTCAE) version 5.0 will be utilized for adverse event reporting. (http://ctep.cancer.gov/reporting/ctc.html)

Appendix 3: Biomarker Management Plan (Attached)

The descriptions and grading scales found in the revised NCI Common Terminology Criteria for Adverse Events (CTCAE) version 5.0 will be utilized for adverse event reporting. (http://ctep.cancer.gov/reporting/ctc.html)
